# Reconstruction of Oxygen Evolution Precatalysts: Effects of Leaching, Electrolyte Concentration and Iron Incorporation

**DOI:** 10.1021/acsnano.6c08336

**Published:** 2026-10-12

**Authors:** Xinzhang Lin, J. Niklas Hausmann, Na An, Suptish Ghosh, Stefan Mebs, Zhe Xue, Holger Dau, Matthias Driess, Prashanth W. Menezes

**Affiliations:** † Department of Chemistry: Metalorganics and Inorganic Materials, 26524 Technische Universität Berlin, Straße Des 17. Juni 135, Sekr. C2, Berlin 10623, Germany; ‡ Department of Materials Chemistry for Catalysis, 74748 Helmholtz−Zentrum Berlin für Materialien und Energie GmbH, Hahn-Meitner-Platz 1, Berlin 14109, Germany; § Department of Physics, 9166 Freie Universität Berlin, Arnimallee 14, Berlin 14195, Germany; ∥ School of Materials Science and Engineering, 66291 North University of China, Xueyuan Road 3, Taiyuan 030051, China

**Keywords:** water electrolysis, oxygen evolution catalysts, structural reconstruction, element leaching, Fe incorporation effect

## Abstract

Fe-containing Ni oxyhydroxides are among the most active alkaline oxygen evolution catalysts, whose structures and performances strongly depend on precursor reconstruction and Fe location. However, achieving precise control over precursor design, reconstruction, and Fe incorporation remains highly challenging. Here, we report the low-temperature synthesis of intermetallic Ni_2_In_3_ nanoparticles as a precursor and uncover their *in situ* reconstruction behavior modulated by electrolyte concentration. Compared with similarly sized Ni nanoparticle-derived catalysts, the Ni_2_In_3_-derived catalysts exhibit significantly enhanced activity and stability. The optimal catalyst, after activation in 6 M KOH with Fe spiking, requires an overpotential of only 284 mV for 10 mA cm^–2^, along with a low Tafel slope of 31 mV dec^–1^. Combined electrochemical analyses, *in situ/ex situ* characterizations, and theoretical calculations reveal that In leaching induces electronic structure modulation and surface undercoordinated site enrichment, thereby facilitating interfacial Fe incorporation to form NiFe active centers. In an anion exchange membrane water electrolyzer, the catalyst demonstrates excellent stability of 260 h at 1 A cm^–2^ at 80 °C. These findings highlight the importance of electrolyte- and precursor-dependent reconstruction in modulating the electronic structure and promoting interfacial Fe incorporation, thereby enabling the design of oxygen evolution electrocatalysts.

## Graphical abstract

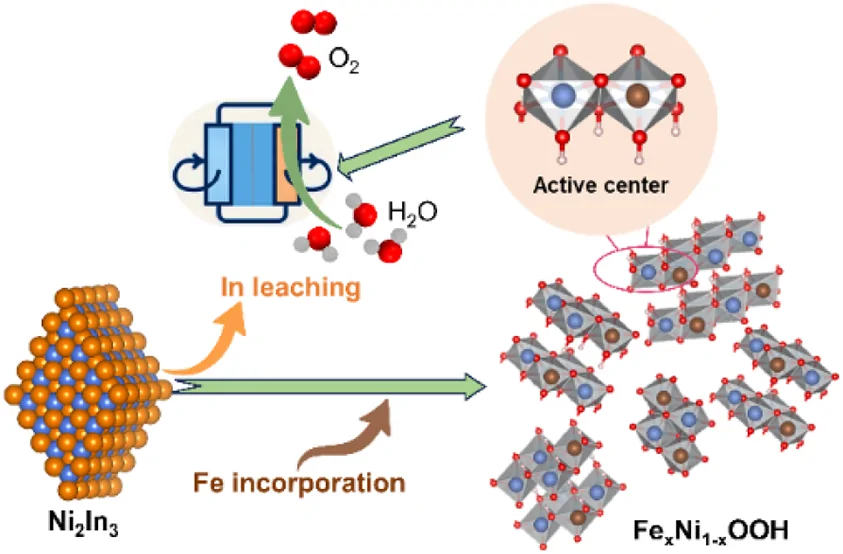

## Introduction

The development of highly active and cost-effective electrocatalysts to overcome the sluggish kinetics of the oxygen evolution reaction (OER) is critical for improving the overall efficiency of electrocatalytic water splitting and other renewable energy conversion technologies.^1–4^ Among the various alkaline OER catalysts, transition-metals, particularly Ni-based systems, have attracted considerable attention due to their low cost and favorable catalytic performance.^5–7^ Most of these materials undergo *in situ* reconstruction during electrochemical operation, forming the actual catalytically active phase of metal oxyhydroxides on the surface or even throughout the bulk.^8,9^ These reconstructed phases are thermodynamically more stable, and their electrified surfaces remain in a dynamic equilibrium with the electrolyte.^10^ However, different precursors and reconstruction conditions can lead to distinct transformation behavior,^11^ resulting in variations in structural porosity, defect density, and local coordination environments, and ultimately accounting for diverse catalytic performance. Recently, intermetallic compounds have emerged as novel precursors for promising OER catalysts, owing to their ordered atomic structures, unique properties, and the selective p-block element leaching under oxidative conditions.^12^ These materials are typically activated and reconstructed in 1 M KOH, and in some cases, may undergo complete element leaching.^8^ However, according to the Pourbaix diagram, several elements, such as indium, tend to form insoluble phases under alkaline OER conditions.^13^ For instance, CoIn_3_ primarily experienced phase separation in 1 M KOH, with indium forming In­(OH)_3_ rather than leaching into the electrolyte.^14^ The potential roles of such nonleaching elements, the reconstruction behavior of these intermetallics in highly concentrated electrolytes, and their influence on critical processes such as Fe incorporation and oxygen evolution remain largely unexplored. Moreover, expanding the library of intermetallic nanoparticles for OER through more accessible synthesis is highly desirable to uncover diverse reconstruction dynamics and local structures of active phases.

As early as 1987, Corrigan reported that trace Fe impurities in the electrolyte could significantly boost OER activity.^15^ In recent years, the Boettcher group demonstrated that pure Ni­(OH)_2_/NiOOH is intrinsically a poor OER catalyst.^16^ The presence of Fe is therefore essential, with undercoordinated Fe located at interfacial sites such as edges or defects exhibiting superior activity compared to bulk-incorporated Fe.^17^ However, the exact role of Fe remains a subject of ongoing debate. Markovic *et al.* proposed that Fe acts as the dynamic catalytic center, with the activity of Fe-MO_*x*_H_*y*_ determined by the average number of Fe sites at the interface.^18^ Nocera *et al.* suggested that Fe^3+^, as a strong Lewis acid, promotes the formation of Ni^4+^ species.^19^ Additionally, Strasser and coworkers proposed that the intrinsic activity of Fe–Ni catalysts originates from the dual-metal-site nature of the reaction centers, which can stabilize OER intermediates.^20,21^ Nevertheless, the coexistence of interfacial Fe and Ni is crucial. Extensive studies have revealed that Fe incorporation is influenced by multiple factors,^22^ including the nature of the precursors,^11^ the structure of the reconstructed host material,^23^ the location of deposited Fe,^17^ its dynamic dissolution/redeposition behavior,^18,24,25^ and the Fe content.^26,27^ However, how to effectively coordinate these factors—such as directing the reconstruction of appropriate precursors into desirable host structures and enriching dynamically stable interfacial Fe sites for efficient OER catalysts remains a significant challenge. In this context, intermetallic precursors, with their distinct reconstruction dynamics and ability to generate abundant interfaces (surfaces, edges, or defects), offer a promising and tunable host for regulating Fe incorporation. Therefore, this work aims to address the following research questions (Scheme 1):

**Scheme 1:**
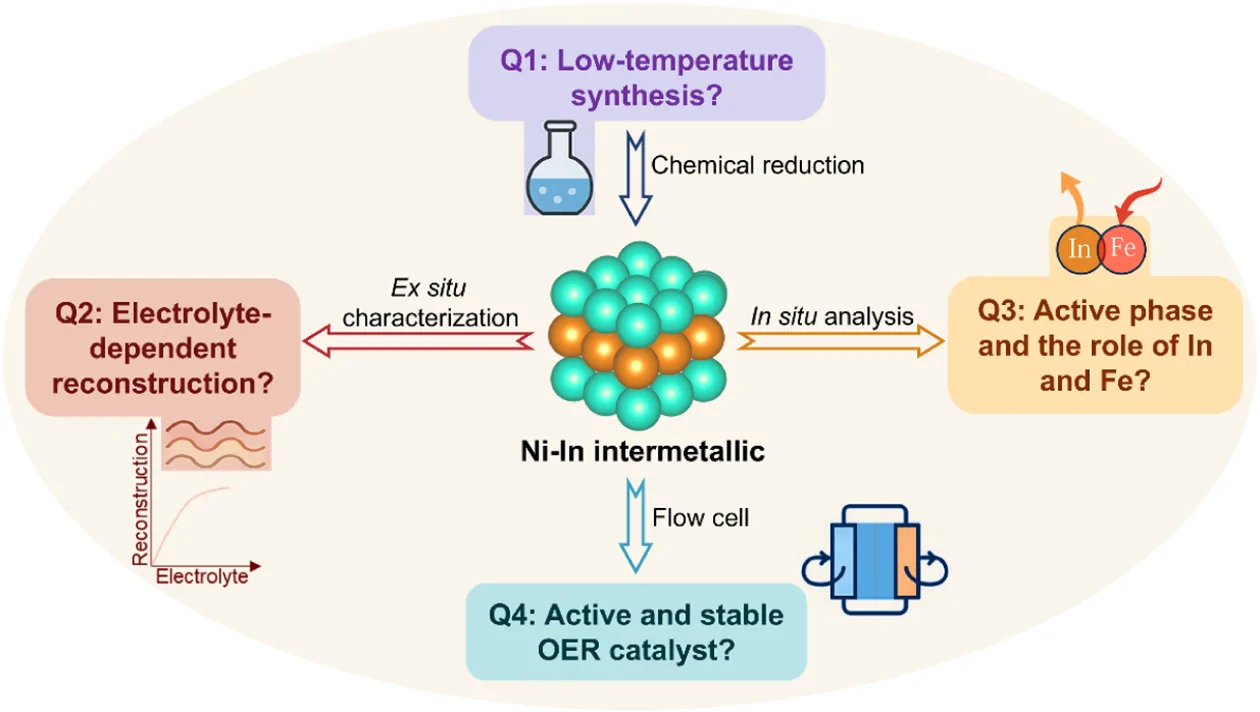
Schematic Illustration of the Key Research Questions Guiding this Work: Ni–In Intermetallics as Precursors → Electrolyte-Driven Reconstruction → Active Phase and Roles of In and Fe in Activation/Catalysis → Emergence of Stable, Active OER Phases Under Industrial Conditions

1) Is it possible to prepare Ni–In intermetallic nanoparticles as a novel precursor for OER catalysis via a more energy-efficient method?
2) How does the electrolyte concentration influence the reconstruction process of intermetallics containing so-called nonleaching elements, and how do the resulting reconstructed phases perform electrochemically?
3) What are the active phase and local structure of its active centers? What roles might indium and iron play during activation and catalysis?
4) Can a highly active and durable OER catalyst, especially under industrial conditions, be developed?

Herein, we employ a chemical reduction method to achieve the low-temperature synthesis of intermetallic Ni_2_In_3_ nanoparticles, replacing the conventional high-temperature solid-state approach. Comprehensive characterization demonstrated the electrolyte-dependent reconstruction involving progressive phase segregation, indium leaching, and interfacial Fe incorporation, as well as enhanced OER activity with the degree of reconstruction. To elucidate the role of indium, Ni nanoparticles with comparable size were synthesized as a reference, enabling a direct comparison of reconstruction behavior and its influence on key processes such as Fe incorporation and OER. To further clarify the role of Fe, both Ni and Ni_2_In_3_ nanoparticles were activated in Fe-free 6 M KOH, commercial 6 M KOH, and additionally subjected to Fe spiking, to evaluate how different precursors and treatment conditions affect Fe incorporation and catalytic performance. The *in situ* Raman spectroscopy and quasi-*in situ* X-ray absorption spectroscopy (XAS) methods were performed to probe the active phase and the local structure of the active centers. Notably, the optimal catalyst established by design in this work delivered a long-term stability of 260 h at 1 A cm^–2^ in an anion exchange membrane water electrolyzer (AEMWE), underscoring its industrial applicability.

## Results and Discussion

### Synthesis and Characterization of Ni_2_In_3_ Nanoparticles

The intermetallic Ni_2_In_3_ nanoparticles were prepared via a chemical reduction method (Figure S1). To the best of our knowledge, this is the first report on a low-temperature synthesis of Ni_2_In_3_ nanoparticles. Compared to conventional methods that require 600 °C and prolonged reaction times, resulting in large particle sizes up to several micrometers,^28^ our approach offers a more energy-efficient and time-saving alternative to obtain nanoparticles. The powder X-ray diffraction (PXRD) confirmed the formation of a pure Ni_2_In_3_ phase (Figure 1a), which crystallizes in the hexagonal system (PDF No. 65–7718) with each Ni atom coordinated by eight In atoms (Figure 1b). Scanning electron microscopy (SEM) with energy-dispersive X-ray elemental (EDX) mapping reveals a small, uniform particle size and a homogeneous distribution of Ni and In (Figures 1c and S2). Transmission electron microscopy further demonstrates crystalline nanoparticles with diameters ranging from 10 to 50 nm (Figures 1d–e and S3) while selected area electron diffraction (SAED) pattern confirms the presence of the (101), (110), (012), (021), (202), (211), and (122) crystalline planes, exhibiting high crystallinity and perfectly matching the XRD peaks. The Ni K-edge X-ray absorption near edge structure (XANES, Figure 1f) of Ni_2_In_3_ nanoparticles exhibits a profile similar to that of Ni metal, with a typical shallow edge rise, reflecting its metallic nature. The extended X-ray absorption fine structure (EXAFS, Figure S4) spectrum was used to reveal the Ni–In and Ni–Ni coordination environments in accordance with the crystal structure.

**Figure 1:**
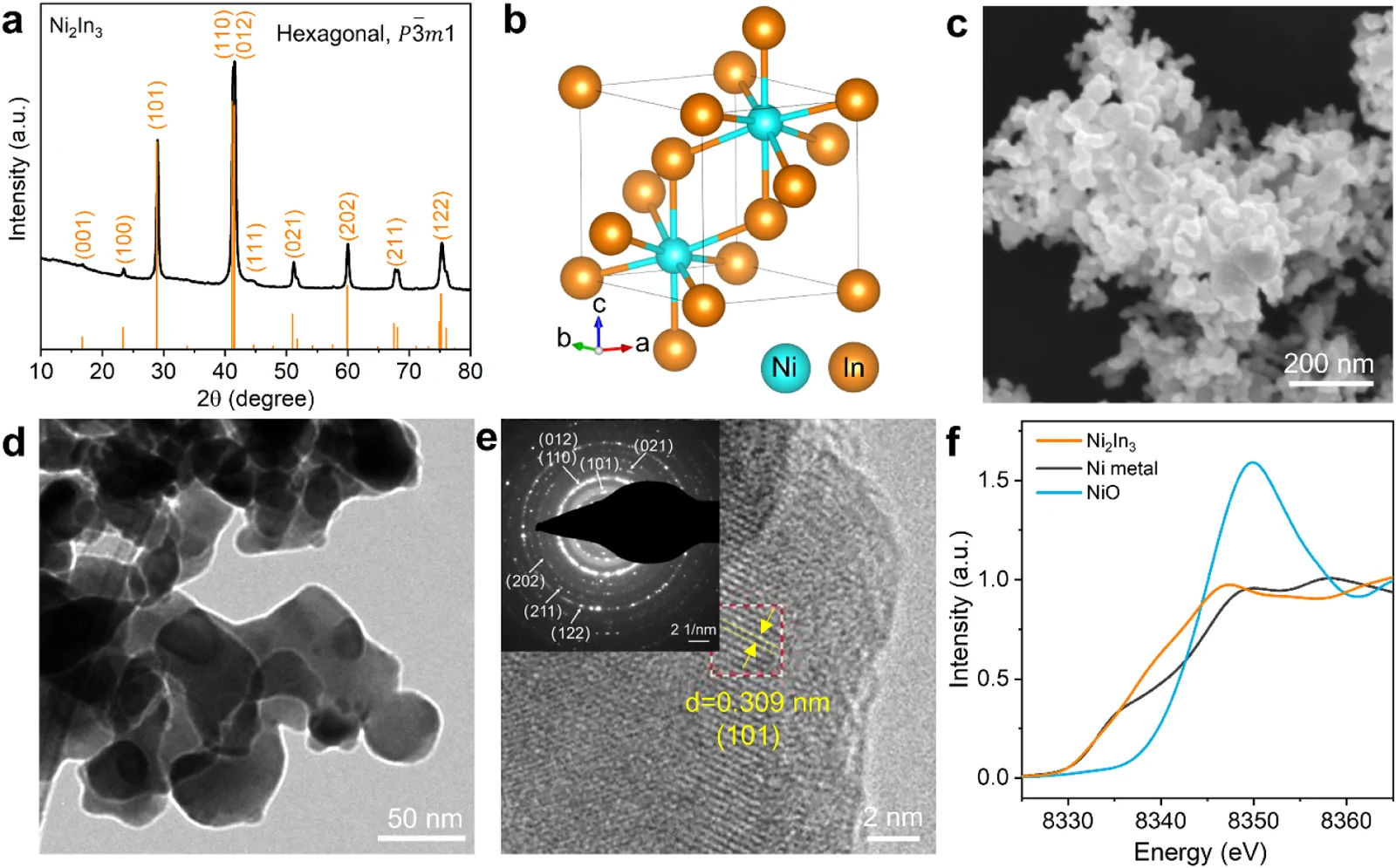
Structural characterization of Ni_2_In_3_ nanoparticles. (a) Powder XRD with a simulated pattern using the unit cell (b) of Ni_2_In_3_ (PDF No. 65–7718). (c) SEM and (d-e) TEM images with lattice spacing marked in yellow. The inset in (e) is the corresponding SAED pattern with crystalline planes marked in white. (f) Ni K-edge XANES spectra of Ni_2_In_3_, reference Ni metal, and NiO.

### Electrolyte-Dependent Reconstruction and Its Effect on OER

To study electrocatalytic behavior, Ni_2_In_3_ nanoparticles were deposited on fluorine-doped tin oxide (FTO) glass plate via electrophoretic deposition (EPD), referred to as Ni_2_In_3_/FTO. PXRD and SEM analyses of Ni_2_In_3_/FTO (Figures S5–S6) confirmed unchanged crystal structure and morphology after EPD. The total loading of Ni_2_In_3_ on FTO was about 0.15 mg, measured by inductively coupled plasma optical emission spectroscopy (ICP-OES). X-ray photoelectron spectroscopy (XPS) analysis indicated that Ni and In coexist in both metallic and oxidized states due to partial surface oxidation (Figure S7). Then, considering that In­(OH)_3_ can be formed in the alkaline OER region according to the Pourbaix diagram (Figure S8), to investigate whether partial removal of indium is possible at higher alkalinity, Ni_2_In_3_/FTO was activated in KOH solutions of varying concentrations (1 M, 2 M, 3 M, and 6 M) through 60 cyclic voltammetry (CV) cycles between 1.1 and 1.7 V_RHE_ at a scan rate of 5 mV s^−1^. 60 CV cycles were chosen, as the reconstruction process under 6 M KOH reaches a quasi-stable state by 60 CVs, enabling comparison of all samples under a unified activation protocol (Figure S9). The potentials were approximated and calibrated using a previously reported equation.^29^ After activation, these catalysts were labeled as Ni_2_In_3_/FTO-*n*M, where *n* represents the concentration. At each KOH concentration, both the Ni (II/III/IV) oxidation peak area and current density increased progressively with CV cycling (Figure 2a), indicating structural reconstruction and the gradual exposure of Ni sites. Concurrently, the oxidation peaks in the range of 1.35–1.50 V_RHE_ shift cathodically, suggesting changes in the electronic structure of Ni. Furthermore, comparison across different concentrations revealed that higher KOH concentrations result in larger oxidation peak areas and current densities, implying that concentrated electrolytes facilitate the exposure of more Ni sites.

**Figure 2:**
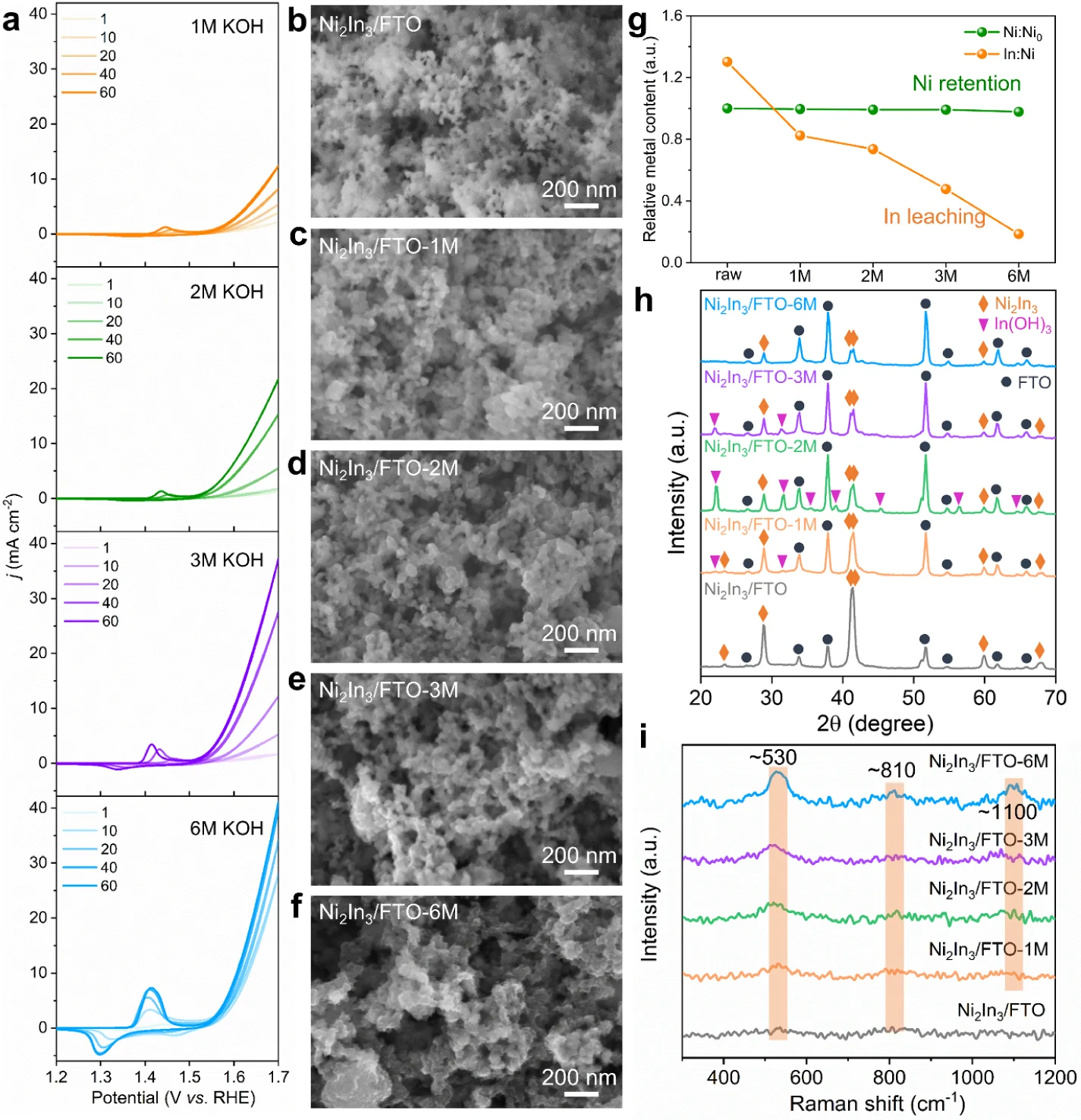
Structural and compositional characterization after activation and reconstruction in different concentrations of KOH. (a) CV curves of samples activated in 1, 2, 3, and 6 M KOH, with the first, 10th, 20th, 40th, and 60th CV curves selected. (b-f) SEM images of the as-prepared sample and the samples activated in 1, 2, 3, and 6 M KOH, respectively. (g) The variations in Ni and In contents as measured by ICP-OES, presented as the ratios of Ni to the initial Ni and In to Ni for different catalysts. (h) Grazing incidence XRD, and (i) Raman spectra of samples activated in different concentrations of KOH. Raman peaks around 530, 810, and 1100 cm^–1^ indicate the presence of Ni oxides or hydroxides.

To investigate the reconstruction process, multiple techniques were employed. First, SEM revealed that increasing the KOH concentration led to significant morphological changes, with progressively larger and rougher aggregates observed on the catalyst’s surface (Figure 2b–f, Figures S10–S13). TEM analysis of Ni_2_In_3_/FTO-6M (Figure S14) further indicated that this morphological evolution was accompanied by a loss of crystallinity, as the initially well-defined crystalline nanoparticles gradually transformed into short-range ordered aggregates with indistinct boundaries. The SAED pattern indicated poor crystallinity and the presence of Ni (oxy)­hydroxides (Figure S14d). Similarly, the ICP-OES analysis was performed to investigate changes in elemental composition, where Ni dissolution was found to be negligible, with only ∼ 2% loss even in 6 M KOH. In contrast, indium leaching became more pronounced with increasing KOH concentration, reaching 80% in Ni_2_In_3_/FTO-6M (Figure 2g). To further investigate compositional variations, grazing incidence X-ray diffraction (GIXRD) was conducted. As the KOH concentration increased, the intensity of the Ni_2_In_3_ peaks gradually decreased (Figure 2h), indicating a deeper reconstruction. Upon activation in 1 M KOH, additional peaks assigned to In­(OH)_3_ (Figure S15) appeared. For Ni_2_In_3_/FTO-2M, more pronounced In­(OH)_3_ peaks indicated that a substantial amount of In­(OH)_3_ remains on the surface despite some indium leaching. This suggests that at lower concentrations, Ni_2_In_3_ primarily undergoes phase segregation, similar to that in CoIn_3_.^14^ With further increase in concentration, the In­(OH)_3_ peaks weaken and nearly disappear for Ni_2_In_3_/FTO-6M, indicating significant indium leaching and the removal of most surface In­(OH)_3_. While no crystalline Ni-containing phases were observed in XRD, Raman spectra provide indications for the presence of Ni oxides or hydroxides (Raman peaks around 530, 810, and 1100 cm^–1^) (Figure 2i).^30–33^ As the KOH concentration increases, the proportion of Ni^δ+^ species correspondingly increases. Furthermore, the XPS confirmed indium leaching, as evidenced by a clear decrease in In 3d peak intensity with increasing concentration (Figure S16). ICP-OES analysis also revealed the presence of trace Fe, with Fe/Ni ratios of 0.006, 0.011, 0.009, and 0.024 in Ni_2_In_3_/FTO-1M, Ni_2_In_3_/FTO-2M, Ni_2_In_3_/FTO-3M, and Ni_2_In_3_/FTO-6M, respectively. In conclusion, we demonstrate that with increasing KOH concentration, Ni_2_In_3_ undergoes progressively deeper reconstruction, likely forming a Ni (oxy)­hydroxides/In­(OH)_3_@Ni_2_In_3_ with trace Fe on the FTO surface. At lower concentrations, phase segregation dominates, while at higher concentrations, significant leaching of In­(OH)_3_ occurs, resulting in a marked decrease.

To assess the OER activity of these catalysts after activation, they were tested in 1 M KOH. A greater degree of reconstruction enhances the OER activity of the reconstructed catalysts (Figure 3a). Accordingly, Ni_2_In_3_/FTO-6M exhibited the highest OER performance with η_10_ = 325 ± 4 mV (η_10_: overpotential at 10 mA cm^–2^). Steady-state Tafel plots revealed similar Tafel slopes for all the samples in the low current density region, suggesting the presence of comparable kinds of active sites (Figure 3b). Nyquist plots obtained from electrochemical impedance spectroscopy (EIS) revealed that Ni_2_In_3_/FTO-6M exhibited the lowest charge transfer resistance (*R*_ct_) (Figure S17 and Table S1), indicating a strong interfacial electron transfer capability. Electrochemical surface area (ECSA) is primarily used to approximate the number of active sites and is usually determined from the double-layer capacitance (C_dl_). However, accurate C_dl_ measurements are very challenging for Ni-based catalysts because of Ni redox/OER interference at high potentials and poor electronic conductivity at low potentials (Figures S18–S19).^34–36^ Therefore, as a more practical alternative, the integration of the Ni­(II/III/IV) reduction peaks at 1.1–1.5 V_RHE_ was used to estimate the quantity of Ni redox-active sites (Figure S20 and Table S2), and the corresponding normalized CV curves indicated their intrinsic activity.^37,38^ Ni_2_In_3_/FTO-1M exhibited the lower normalized current density, while the other three samples showed comparable performance (Figure 3c). This proves that the variations in apparent activity mainly arise from the differences in the number of active sites.

**Figure 3:**
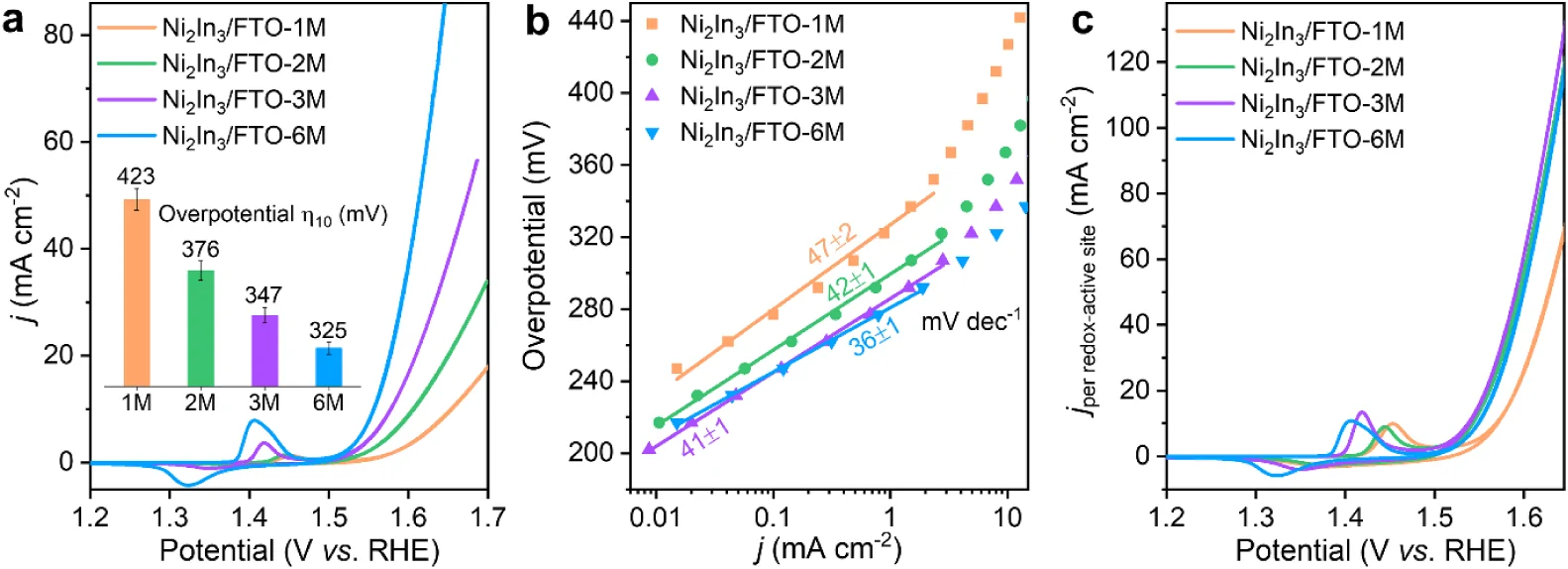
Electrochemical data of all activated Ni_2_In_3_/FTO tested in 1 M KOH. (a) CV curves with a scan rate of 5 mV s^–1^ (Inset: the OER overpotential at 10 mA cm^–2^). Potentials are *iR* compensated (see SI for details). (b) Steady-state Tafel analysis. The corresponding Tafel slopes are indicated in mV per decade; errors are 1s confidence intervals from three independent measurements. (c) The normalized CV curves based on the amount of Ni redox-active sites (normalized as described in Section 1.7 in SI).

### Investigation of the Active Structure

To explore the role of indium, Ni nanoparticles (NPs) with comparable sizes to those of Ni_2_In_3_ were synthesized as a reference (Figures S21–S22). Ni NPs were also deposited on FTO to obtain Ni NP/FTO with a metal loading of 0.14 mg and activated in 6 M KOH to obtain Ni NP/FTO-6M (Figure S23a). To eliminate the effect of Fe impurity in electrolytes, Ni_2_In_3_/FTO and Ni NP/FTO were also activated in Fe-free 6 M KOH (Figure S23b–c), which was purified following a reported procedure.^16^ The activated samples were denoted as Ni_2_In_3_/FTO-p6M and Ni NP/FTO-p6M, respectively. To further investigate the effect of Fe incorporation, Fe spiking was carried out on Ni_2_In_3_/FTO-6M and Ni NP/FTO-6M to increase the Fe content, hereafter denoted as Ni_2_In_3_/FTO-6M-Fe and Ni NP/FTO-6M-Fe (Figure S24). In addition, the benchmark catalyst Fe_*x*_Ni_1–*x*_OOH was synthesized as a reference. EDX and ICP-OES confirmed its composition to be approximately Fe_0.08_Ni_0.92_OOH (Figure S25).

Various characterization techniques were employed to study all the obtained samples. ICP-OES revealed a Fe: Ni: In atomic ratio of 7:73:20 in Ni_2_In_3_/FTO-6M-Fe, with the Fe content similar to the reference Fe_0.08_Ni_0.92_OOH (Figure S26). In contrast, Ni NP/FTO-6M-Fe exhibited a lower Fe incorporation, with an Fe: Ni ratio of only 2:98. The Ni_2_In_3_/FTO-6M-Fe displayed a porous and rough morphology on the surface (Figure 4a and Figure S27), indicating a poorly crystalline nature, similar to that of Ni_2_In_3_/FTO-6M (Figure S14). The SAED patterns suggested that the primary phase is Ni (oxy)­hydroxides (inset of Figure 4a). On the contrary, the SAED patterns revealed that Ni_2_In_3_ remains the dominant phase in Ni_2_In_3_/FTO-p6M, indicating limited reconstruction (Figure S28). On the other hand, Ni NP/FTO-6M-Fe and Ni NP/FTO-6M display large, bulky structures on the surface with similar phase characteristics (Figures S29–S31), indicating the aggregation and growth of Ni NPs after activation. *Ex situ* Raman spectra revealed the pronounced differences between the Ni_2_In_3_-derived and Ni NP-derived samples (Figure 4b). The Ni_2_In_3_-derived samples primarily consisted of Ni (oxy)­hydroxides, with more pronounced characteristic peaks corresponding to Ni^2+^–O.^30,31^ In contrast, the Ni NP-derived samples exhibited partial retention of Ni oxyhydroxides,^39^ where Ni has a higher oxidation state, as further confirmed by XPS (Figures S32–S33).

**Figure 4:**
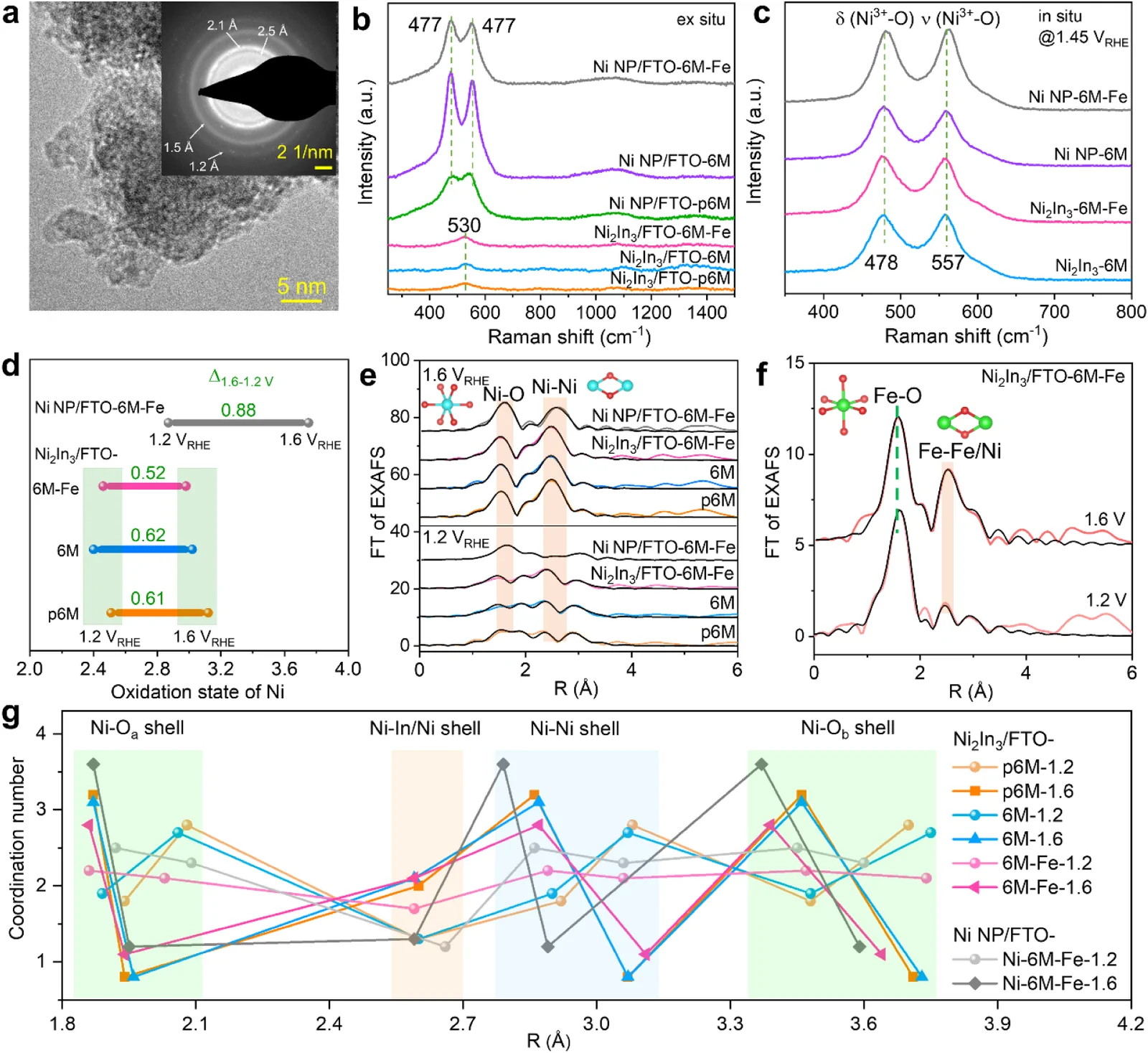
Structure, oxidation state, and local structural change of Ni and Ni_2_In_3_. (a) TEM image of Ni_2_In_3_/FTO-6M-Fe with the corresponding SAED pattern. (b) The *ex situ* Raman spectra of all Ni_2_In_3_- and Ni NP-derived samples. (c) The *in situ* Raman spectra at 1.45 V_RHE_ of Ni_2_In_3_-6M, Ni_2_In_3_-6M-Fe, Ni NP-6M, and Ni NP-6M-Fe deposited on Pt foil. Prior to spectral acquisition, all samples were activated (See Section 1.6 in SI), and the potential was held for 5 min. (d) Oxidation states of Ni in Ni_2_In_3_-derived catalysts and Ni NP/FTO-6M-Fe determined by quasi-*in situ* XANES. The quasi-*in situ* (e) Ni K-edge and (f) Fe-edge EXAFS data of the activated samples by 60 CV cycles freeze-quenching after operation at 1.2 and 1.6 V_RHE_ for over 20 min. (g) The coordination number of the different coordination shells in EXAFS simulations (see Tables S4 and S6). There are two distances for every shell of Ni–O_a_, Ni–Ni, and Ni–O_b_.

*In situ* Raman spectroscopy was employed to investigate the active phases of Ni_2_In_3_-6M, Ni_2_In_3_-6M-Fe, Ni NP-6M, and Ni NP-6M-Fe. All tested samples exhibited characteristic peaks of NiOOH in the OER potential region, located at approximately 478 and 560 cm^–1^, corresponding to δ­(Ni^3+^–O) and ν­(Ni^3+^–O),^39^ respectively (Figure 4c). However, for Ni_2_In_3_-derived samples, these characteristic peaks only began to appear when the potential reached 1.40 V_RHE_ (Figure S34). In contrast, Ni NP-derived samples already displayed NiOOH features under OCP conditions, with these peaks becoming prominent starting from 1.25 V_RHE_, indicating that the oxidation of Ni occurs more easily. This difference may be attributed to the presence of indium in Ni_2_In_3_-derived catalysts, which modulates the electronic structure of Ni and strengthens the Ni–O bond, thus requiring a higher potential for oxidation. The peak intensity ratio of ν­(Ni^3+^–O) to δ­(Ni^3+^–O) suggests that Fe incorporation modulates the local Ni–O environment in Ni NP-derived catalysts as previously reported^40^ but has a limited impact in Ni_2_In_3_-derived ones, suggesting that Fe may be primarily located at the interface (Figure S35).

To investigate the dynamic changes of the catalysts, quasi-*in situ* XAS measurements were performed by freeze-quenching the CV activated electrodes after operation at potentials below (1.2 V_RHE_) and above (1.6 V_RHE_) the redox peak for over 20 min. Quasi-*in situ* Ni K-edge XANES reveals that Ni in Ni_2_In_3_-derived catalysts oxidizes to approximately +3 at 1.6 V_RHE_ (Figure 4d, Figure S36a, and Table S3), indicative of the formation of oxyhydroxide phases. In contrast, Ni in Ni NP/FTO-6M-Fe exhibits a higher oxidation state of +3.8, resulting from the relatively low Fe content (Figure S26).^41^ Even at 1.2 V_RHE_, it remains relatively high (+2.9), in agreement with the Raman results, suggesting a more irreversible Ni redox process in the Ni NP-derived catalysts (Figure 4d, Figure S36b–c, and Table S3). Comparing three Ni_2_In_3_-derived catalysts, the oxidation state of Ni at 1.6 V_RHE_ slightly decreases with increasing Fe content. This may be attributed to the increased electron density around the O atoms bridging Fe and Ni due to Fe incorporation, which suppresses the oxidation of adjacent Ni sites,^42^ also indicating the formation of O-bridged NiFe active centers. The difference in oxidation states between 1.2 and 1.6 V_RHE_ (Δ_1.6–1.2 V_) is correlated with the Ni redox activity. Ni_2_In_3_/FTO-6M-Fe exhibits the smallest Δ _1.6–1.2 V_ value of 0.52 among Ni_2_In_3_-derived catalysts (Figure 4d), suggesting that Fe incorporation can reduce the Ni redox activity, probably due to Fe atoms occupying the interfacial redox-active Ni sites.^43^ Quasi-*in situ* Fe K-edge XANES analysis suggests changes in the Fe coordination environment upon oxidation of the Ni_2_In_3_/FTO-6M-Fe material at 1.6 V_RHE_ (Figure S36d), with changing edge features being in line with a transition from Fe^3+^O_5_ to Fe^3+^O_6_ coordination at 1.6 V_RHE_.^44^ Also, the simulation results of the Fe EXAFS spectra support a coordination number increase at 1.6 V_RHE_, possibly associated with additional μ-oxo bridging to a neighboring Ni ion (Table S8). The suggested coordination number change is likely attributed to Fe being predominantly located at interfacial sites with coordination unsaturation.^45^

Quasi-*in situ* EXAFS analysis was performed to study the local structure and coordination environment of Ni and Fe. The EXAFS data could successfully be simulated with structural models of metal (oxy)­hydroxides (Figures 4e and S34). In the layered structure composed of edge-sharing MO_6_ octahedra, the applied model considers three coordination shells: Ni–O_a_, Ni–Ni, and Ni–O_b_, each with two distinct shell-distances corresponding to different oxidation states. The coordination numbers of these respective shells further provided insights into the distribution of oxidation states within the materials. We performed two kinds of fitting: one including only the six shells of nickel (oxy)­hydroxides, and another incorporating an additional Ni–In/Ni–Ni shell representing the as-prepared phase. The inclusion of this extra shell significantly improved the fitting quality, not only revealing the presence of residual In but also confirming the reliability of our fitting strategy (Tables S4–S8). In the Ni–O_a_ shell, the shorter Ni–O distance exhibits a higher coordination number at 1.6 V_RHE_, corresponding to a higher oxidation state, while the longer Ni–O distance dominates at 1.2 V_RHE_, indicating a lower oxidation state (Figure 4g). In the Ni–Ni and Ni–O_b_ shells, the trends were similar. Most importantly, the coordination number trends remained similar under the same potential across the three Ni_2_In_3_-derived catalysts, suggesting that Fe has minimal impact on the local coordination environment of Ni. Ni NP/FTO-6M-Fe exhibits a shorter Ni–Ni distance at 1.6 V_RHE_, indicating a more distorted structure and a higher oxidation state during OER. Moreover, the coordination number of Ni–In in the reconstructed catalysts was approximately 2, compared to 8 in the pristine Ni_2_In_3_, indicating that roughly 25% of indium was retained, in good agreement with the ICP-OES results. Compared with the Fe K-edge EXAFS spectrum of bare FTO with strong Fe–Fe shell features, our samples predominantly showed stronger Fe–O coordination (Figure S38). In conventional layered NiFeOOH catalysts, the pronounced Fe–M (M = Fe or Ni) coordination peak is generally associated with the incorporation of Fe into a well-defined oxyhydroxide lattice.^41,43^ In contrast, Ni_2_In_3_/FTO-6M-Fe exhibits a dominant Fe–O coordination shell below 2 Å and two relatively weak Fe–M peaks between 2 and 3 Å, particularly at 1.2 V_RHE_ (Figure 4f), suggesting a local Fe environment distinct from that of typical NiFeOOH. Quantitative EXAFS fitting further shows that the Ni–M (M = Ni, Fe, or In) coordination number in the first shell remains 6 at both 1.2 and 1.6 V_RHE_ (Tables S4 and S6), indicating a largely preserved Ni-based framework. The Ni–O coordination number remains approximately 4 at both potentials, indicating a coordinatively unsaturated Ni environment consistent with defects generated by In leaching. In contrast, at 1.2 V, the Fe–M and Fe–O coordination numbers are approximately 5.3, suggesting that Fe is initially undercoordinated. At 1.6 V_RHE_, the coordination number increases to 6.1, suggesting potential-induced structural reorganization toward a more ordered local environment under OER conditions. However, the Fe–M peak position remains nearly unchanged, unlike the noticeable potential-dependent shift reported for lattice-incorporated Fe in conventional NiFeOOH.^41^ These observations suggest that the initially incorporated Fe species are likely associated with defect-rich interfacial regions.

### Investigating the Origin of Activity: The Roles of In and Fe

Ni_2_In_3_/FTO and Ni NP/FTO were subjected to three different treatments: Fe-free 6 M KOH, commercial 6 M KOH, and additional Fe spiking, resulting in six distinct samples. The OER activity of these reconstructed samples, along with the in-house produced Fe_*x*_Ni_1–*x*_OOH, was evaluated in 1 M KOH. Ni_2_In_3_/FTO-6M-Fe exhibited the best OER activity, even slightly outperforming Fe_*x*_Ni_1–*x*_OOH/FTO and commercial IrO_2_ (Figure S39). The order of η_10_ is as follows: Ni_2_In_3_/FTO-6M-Fe < Ni NP/FTO-6M-Fe < Fe_*x*_Ni_1–*x*_OOH/FTO < Ni_2_In_3_/FTO-6M ≈ Ni NP/FTO-6M < Ni_2_In_3_/FTO-p6M ≈ Ni NP/FTO-p6M (Figures 5a and S40). Moreover, when comparing the enhancement after Fe spiking, Ni_2_In_3_/FTO-6M showed a greater increase in activity than Ni NP/FTO-6M, indicating that the nature of the host material also plays an important role in Fe incorporation and activity.^46^ Ni_2_In_3_/FTO-6M-Fe exhibited the lowest Tafel slope of 31 mV dec^–1^, closely approaching that of Fe_*x*_Ni_1–*x*_OOH/FTO (34 mV dec^–1^) (Figure 5b). Under the same treatment conditions, Ni_2_In_3_-derived catalysts consistently showed slightly lower Tafel slopes than Ni NP-derived catalysts, suggesting enhanced electron transfer kinetics. Although the interfacial Ni–Fe reaction centers may predominantly contribute to the overall activity, their exact number is difficult to quantify. Therefore, estimating the quantity of interfacial sites by integrating the Ni­(II/III/IV) reduction peak area (Figure S41 and Table S9) remains a valuable approach. The normalized CV curves based on the number of interfacial sites reveal the following order of η_10_: Ni_2_In_3_/FTO-6M-Fe < Ni NP/FTO-6M-Fe ≈ Fe_*x*_Ni_1–*x*_OOH/FTO < Ni_2_In_3_/FTO-6M ≈ Ni NP/FTO-6M < Ni_2_In_3_/FTO-p6M < Ni NP/FTO-p6M (Figure 5c), suggesting that the Ni_2_In_3_/FTO-6M-Fe exhibits the highest intrinsic activity.

**Figure 5:**
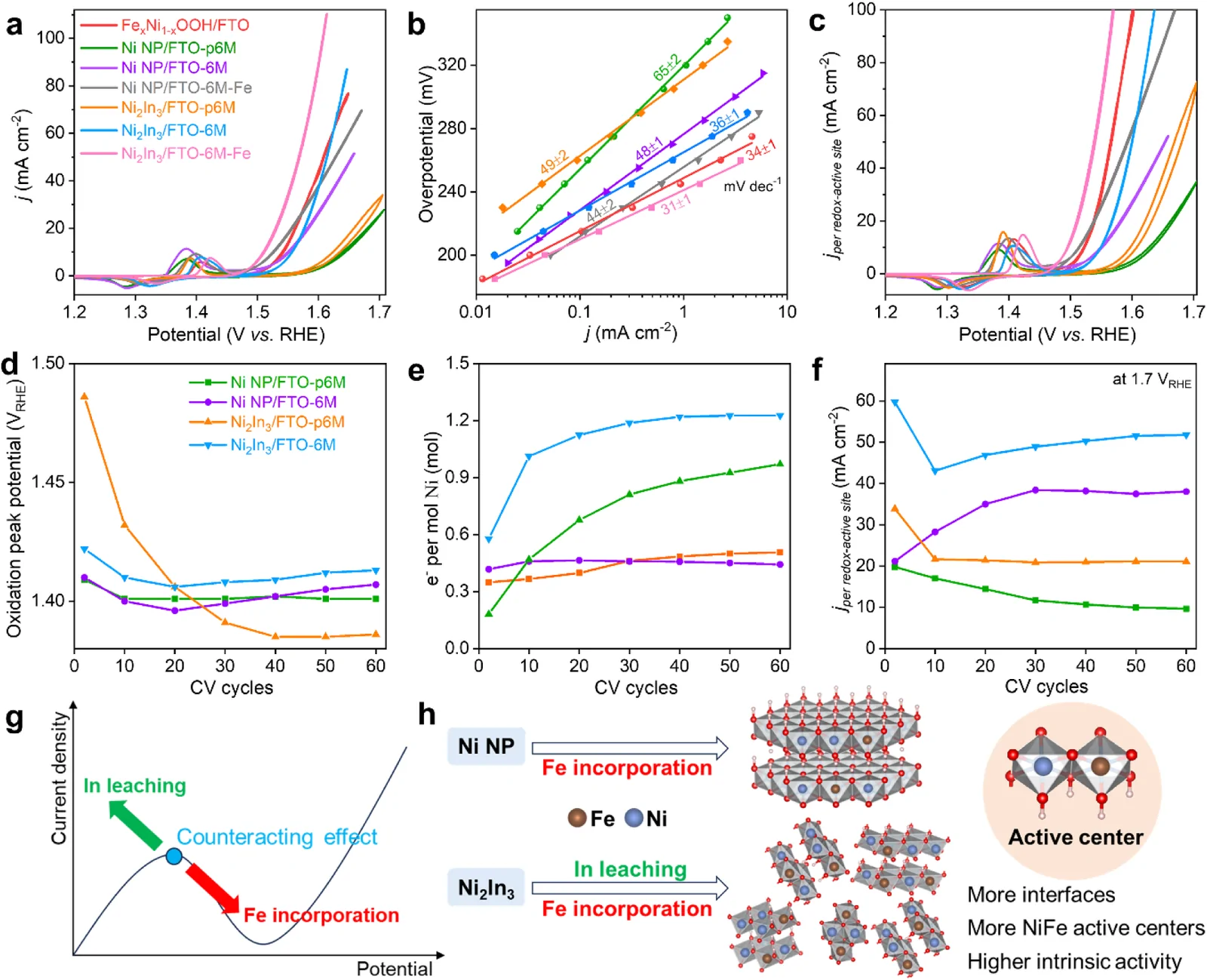
Comparison of electrochemical data tested in 1 M KOH and the activation curves in 6 M KOH of Ni_2_In_3_ or Ni NP derived samples. (a) CV recorded at a scan rate of 5 mV s^–1^. Potentials are *iR* compensated (see SI for details). (b) Steady-state Tafel plots and (c) normalized CV curves based on the number of redox-active sites in 1 M KOH of all the samples. Evolution of (d) oxidation peak potentials, (e) numbers of Ni redox-active sites per mole of Ni, and (f) normalized current densities at 1.7 V_RHE_ based on Ni redox-active sites during CV cycling for Ni_2_In_3_/FTO and Ni NP/FTO activated in Fe-free and commercial 6 M KOH, respectively. (g) Indium leaching and Fe incorporation exert opposite effects on the oxidation peak shift. (h) Schematic illustration of reconstruction differences between Ni NP- and Ni_2_In_3_-derived catalysts.

To elucidate the origin of the catalytic activity differences among these catalysts, we conducted a systematic study. At first, the activity of indium is worth exploring, especially given that several reports have suggested its potential activity.^47,48^ To this end, indium particles were synthesized (Figure S42), activated, and exhibited no observable OER activity (Figure S43). Furthermore, we monitored the leaching behavior of indium particles after CV cycling through ICP-OES. The results indicated that a substantial amount of indium, approximately 80%, leached out in 6 M KOH. Even in 1 M KOH, around 10% of indium exhibited leaching. Although the amphoteric nature of In­(OH)_3_ remains under debate, indium leaching was indeed observed here, possibly existing as In­(OH)_4_^–^ at a low concentration.^13,49^ This interesting finding appears to deviate from the general expectation given by the Pourbaix diagram (Figure S8).

To study the role of indium leaching and rule out the influence of Fe, we first compare the CV curves of Ni_2_In_3_/FTO and Ni NP/FTO activated in Fe-free 6 M KOH (Figure S23). The potential of the Ni­(II/III/IV) oxidation peak for Ni_2_In_3_/FTO-p6M exhibited a gradually cathodic shift with CV cycling, while that of Ni NP/FTO-p6M remained almost unchanged, suggesting that indium leaching has a significant effect on the electronic structure of Ni during reconstruction (Figure 5d). Both the current density of the oxidation peak and the number of redox-active sites per mol Ni, (Figures S44–S45) exhibit a more pronounced continuous increase for Ni_2_In_3_/FTO-p6M during activation compared with Ni NP/FTO-p6M (Figures 5e and S42), which can be attributed to indium leaching that exposes more Ni sites to the electrolyte. Ni_2_In_3_/FTO-p6M also exhibits slightly higher intrinsic activity (Figure 5f). Therefore, it suggests that indium leaching not only exposes more interfacial sites, but also modulates the electronic structure of Ni, resulting in a final catalyst with both an increased number of active sites and a slightly enhanced intrinsic activity.

To assess the effect of Fe incorporation, the activation CV curves in Fe-free and commercial 6 M KOH were compared. The comparison between Ni NP/FTO-6M and Ni NP/FTO-p6M confirms that Fe incorporation causes an anodic shift of the oxidation peak, a slight decrease in peak current density, and a reduced redox peak area (Figures 5d–e and S42), in agreement with previous studies.^40,41^ In contrast, for Ni_2_In_3_/FTO-6M, the activation process is influenced by two concurrent factors: indium leaching and Fe incorporation (Figure 5g). Accordingly, the trends of the activation curves reflect the collective or counteracting effect of both processes. The oxidation peak of Ni_2_In_3_/FTO-6M exhibited an anodic shift after the combined effects, with the final peak potential significantly higher than that of Ni_2_In_3_/FTO-p6M (Figure 5d). Both samples show a comparable increase in peak current density, indicating a negligible influence of Fe on peak current (Figure S46). During CV cycling, the number of interfacial redox-active sites increases for both samples due to indium leaching; however, the increase is slightly slower for Ni_2_In_3_/FTO-6M, owing to the slight counteracting effect of Fe incorporation. Compared with Ni NP, Fe has a smaller impact on the redox peak area of Ni_2_In_3_ (Figure 5e). Both Ni NP/FTO-6M and Ni_2_In_3_/FTO-6M exhibited higher normalized current densities based on the redox peak area (Figures S47–S48) compared to Fe-free ones, highlighting the effect of Fe on intrinsic activity through the formation of possible NiFe active centers^17,20,21^ (Figure 5f).

To further investigate the role of residual In, density functional theory (DFT) calculations were performed based on simplified NiOOH models (Figure S49a; see SI for details). Fe incorporation lowers the theoretical overpotential from 0.60 V for NiOOH to 0.39 V, whereas Fe/In co-doped NiOOH exhibits a comparable value of 0.40 V (Figure S49b). This indicates that Fe substantially improves the intrinsic OER energetics, while residual In provides no appreciable additional enhancement. Nevertheless, the presence of In modifies the local geometric and electronic environments of adjacent Ni sites, as evidenced by the contraction of one Ni–O bond from approximately 2.04 to 1.88 Å, the downshift of the Ni d-band center from – 2.47 to – 2.82 eV, and the altered charge-transfer characteristics of adsorbed *OH (Figure S49c–e). These calculations suggest that In leaching facilitates Fe incorporation, thereby enhancing the intrinsic OER activity, whereas residual In may regulate the local electronic structure of Ni but appears to have a limited effect on the activity.

Overall, the differences in OER activity among the three Ni NP-derived catalysts are primarily attributed to varying degrees of Fe incorporation. In contrast, the enhanced activity of the Ni_2_In_3_-derived catalysts originates from a greater density of exposed active sites due to In leaching, as well as enhanced intrinsic activity arising from the synergistic effects of indium leaching-induced Ni electronic modulation and increased Fe incorporation (Figure 5h). During electrochemical reconstruction, the selective leaching of In is expected to generate a defect-rich and locally undercoordinated Ni–O framework, including cation-deficient sites and unsaturated surface oxygen species. These structural features may provide energetically favorable adsorption and trapping sites for Fe-containing species from the electrolyte, thereby facilitating their incorporation into the reconstructed Ni-based oxyhydroxide phase. In comparison, the reconstruction of Ni nanoparticles does not involve the selective removal of a second metallic component and is therefore expected to generate fewer vacancies and defect sites, resulting in lower Fe uptake under identical activation conditions. The greater extent of Fe incorporation enables the formation of more catalytically active Ni–Fe motifs, thereby contributing to the enhanced OER activity. Although Ni NP/FTO-6M-Fe exhibits a high average Ni oxidation state of +3.8, which has been reported in some studies to correlate with enhanced activity,^19,50^ our results demonstrate that the formation of abundant interfacial NiFe active centers seems to play a more critical role in boosting OER performance.^20,21^

### Practical Application Potential

To investigate the potential of practical applications, Ni_2_In_3_ was deposited on Ni foam via EPD and activated in 6 M KOH with Fe spiking, designated as Ni_2_In_3_/NF-6M-Fe. Electrochemical testing in a three-electrode system revealed that Ni_2_In_3_/NF-6M-Fe showed good OER activity with an overpotential (η_100_) of 291 mV (Figure 6a) and displayed promising stability, sustaining over 100 h under the current densities of 10, 50, and 200 mA cm^–2^, respectively (Figure S50). Recently, anion exchange membrane water electrolyzers (AEMWE) have emerged as a promising technology for sustainable hydrogen production.^51–53^ Therefore, the Ni_2_In_3_/NF-6M-Fe catalyst was assessed as the anode in an AEMWE cell with Ni_4_Mo/MoO_2_@NF as the cathode under industrially relevant conditions. Fast-scanning (Figure S51) and stepwise (Figures 6b and S52) polarization curves at different temperatures confirmed the good catalytic activity of Ni_2_In_3_/NF-6M-Fe. Notably, in an AEMWE cell operating at 80 °C and 1 A cm^–2^, the catalyst exhibited outstanding operational stability, with the cell voltage remaining nearly constant within the fluctuation range over 260 h (Figure 6c). Poststability characterization suggests the persistence of the reconstructed Fe-containing Ni-based active phase after 260 h of operation, as evidenced by the retained catalyst layer, homogeneous Ni, Fe, In, and O distributions, unchanged metal oxidation states, and predominantly amorphous nature (Figures S53–S56). Motivated by these promising results, future studies will aim to provide deeper insights into its performance and stability.

**Figure 6:**
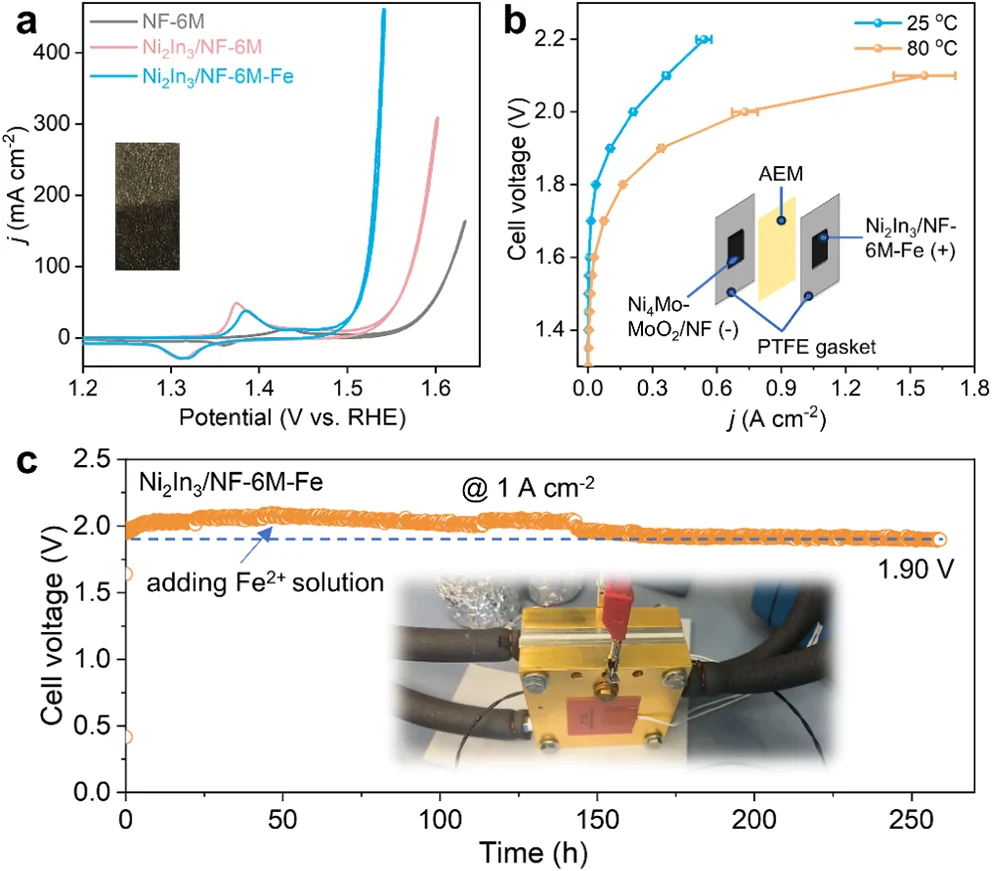
Electrochemical data of Ni_2_In_3_/NF-6M-Fe in 1 M KOH. (a) CV curves in a three-electrode system with a scan rate of 5 mV s^–1^. Potentials are *iR* compensated (see SI for details). (b) Polarization curves with error bars based on stepwise chronoamperometry (SI for more information) at different temperatures and (c) the stability test via chronopotentiometry at 1 A cm^–2^ at 80 °C in an AEMWE cell with Ni_2_In_3_/NF-6M-Fe as anode and Ni_4_Mo/MoO_2_@NF as cathode. The inset shows a photo of the AEMWE cell used in this study.

## Conclusion

In summary, we report for the first time the low-temperature synthesis of intermetallic Ni_2_In_3_ NPs via chemical reduction, thereby addressing research question Q1 from the introduction. With respect to Q2, this material was employed as a novel precursor for OER catalysis, and the reconstruction degree was deliberately controlled by adjusting the electrolyte concentration: phase segregation dominates at low concentrations, whereas indium leaching prevails at higher concentrations. The variations in the apparent activity of the reconstructed samples mainly arise from differences in the number of active sites. To address Q3, the reconstruction behaviors and catalytic activities of Ni and Ni_2_In_3_ NPs as precursors were compared. Our findings indicate that indium leaching not only exposes more interfacial sites but also modulates the electronic structure of Ni, resulting in a final catalyst with both an increased number of active sites and a slightly enhanced intrinsic activity. Compared with Ni NP-derived catalysts, Fe incorporation in Ni_2_In_3_-derived catalysts may mainly occur at the interface, exerting a smaller impact on the Ni redox properties but significantly enhancing the intrinsic activity through the formation of NiFe active centers. Therefore, the superior OER activity primarily originates from the synergistic effects of a higher density of interfacial sites, indium-induced electronic modulation of Ni, and enhanced interfacial Fe incorporation. Regarding Q4, the Ni_2_In_3_/NF-6M-Fe catalyst demonstrates excellent OER activity and stability for 260 h at 1 A cm^–2^ at 80 °C in an AEMWE cell. This work offers valuable insights into the rational design and synthesis of advanced OER catalysts by emphasizing the role of electrolyte- and precursor-dependent reconstruction, particularly in modulating electronic structure and enabling interfacial Fe engineering.

## Experimental Section

### Low-Temperature Synthesis of Ni_2_In_3_ Intermetallic Nanoparticles

Using a Schlenk apparatus, NiCl_2_ 6H_2_O (237 g/mol, 0.44 mmol, 103 mg), InCl_3_ (221 g/mol, 0.56 mmol, 123 mg), and poly­(2-ethyl-oxazoline) (M.W. 50,000, 0.6 g) were added to a 100 mL three-necked flask. The system was evacuated and backfilled with nitrogen three times to ensure an inert atmosphere. Next, 15 mL of degassed tetraethylene glycol (TEG) was added to the flask, and the flask was stirred at 600 rpm. The reaction mixture was then heated to 110 °C under vacuum for 1 h, with the thermocouple directly inserted into the reaction solution (Figure S1). Afterward, the temperature was increased to 150 °C, and a freshly prepared solution of NaBH_4_ (37.8 g/mol, 5.0 mmol, 190 mg) in 5 mL of degassed TEG was added dropwise over 15 min. Following the complete addition, the temperature was raised to 230 °C and maintained for 2 h. After heating was stopped, the solution was allowed to cool naturally to room temperature. The resulting product was then isolated by centrifugation at 8500 rpm for 5 min, washed three times with 60 mL ethanol and once with 40 mL acetone, and then dried in air to yield the desired black powder.

### Electrochemical Measurements

Electrochemical measurements were conducted in a three-electrode configuration using 1 M KOH (30 mL) and a potentiostat (SP-200, BioLogic). FTO-supported catalysts were used as the working electrode, a Pt wire as the counter electrode, and a Hg/HgO electrode as the reference. All potentials were converted to the RHE scale using a calibrated value of 0.931 V with *iR* correction. The catalysts were activated by 60 CV cycles (1.1–1.7 V_RHE_). The uncompensated resistance was determined by impedance measurements, and 90% *iR* compensation was applied during measurements, followed by 10% correction during data processing. EIS was performed at 1.56 V_RHE_ in the frequency range of 100 kHz to 1 mHz. Tafel slopes were obtained from steady-state potentiostatic measurements with 15 mV steps, averaging the current over 180 s. The intrinsic activity was evaluated by normalizing the current density to the charge associated with the Ni­(II/III/IV) redox process (1.1–1.5 V_RHE_). All measurements were repeated at least three times. More details are described in the Supporting Information.

## Supplementary Material



## Notes

General considerations, chemicals and materials, characterization methods and data (SEM, TEM, XRD, XPS, XAS, *etc*.), synthesis of reference catalysts, additional electrochemical measurements, integration of reduction peaks in CV curves (PDF)
