# Supplementary material for: Reconstruction of Oxygen Evolution Precatalysts: Effects of Leaching, Electrolyte Concentration and Iron Incorporation
Source: ACS Nano. 2026 Oct 12;20(42):28268–79. doi: 10.1021/acsnano.6c08336 (PMC13718603; doi:10.1021/acsnano.6c08336)
Supplement: Supplementary file 1 [file nn6c08336_si_001.pdf]

Supporting Information

Reconstruction of Oxygen Evolution Precatalysts: Effects of Leaching, Electrolyte Concentration and Iron Incorporation

Xinzhang Lin,[†] *J. Niklas Hausmann,*[||] *Na An,*[||] *Suptish Ghosh,*[†,||] *Stefan Mebs,*[§] *Zhe Xue,*[‡] *Holger Dau,*[§] *Matthias Driess,*[†,*] *Prashanth W. Menezes*[†,||,*]

[†] Department of Chemistry: Metalorganics and Inorganic Materials, Technische Universität Berlin, Straße des 17. Juni 135, Sekr. C2, 10623 Berlin, Germany

[||] Department of Materials Chemistry for Catalysis, Helmholtz–Zentrum Berlin für Materialien und Energie GmbH, Hahn-Meitner-Platz 1, 14109 Berlin, Germany

[§] Department of Physics, Freie Universität Berlin, Arnimallee 14, 14195 Berlin, Germany

[‡] School of Materials Science and Engineering, North University of China, Xueyuan Road 3, Taiyuan 030051, China

1. Experimental section

1.1. Chemicals and materials

Nickel(II) chloride hexahydrate ($NiCl_2$ $6H_2O$, 98%) was purchased from Grüssing GmbH. Indium(III) chloride ($InCl_3$, 98%), tetraethylene glycol (TEG, 99%), poly(2-ethyl-2-oxazoline) ($(C_5H_9NO)_n$, M. W. 50000, PEO), and 1 M aqueous potassium hydroxide (KOH) were purchased from Thermo Fisher Scientific. Sodium borohydride ($NaBH_4$, 95%) was purchased from TCI. The water used throughout the experiments was high-purity H_2O with 18.2 MΩ.cm, obtained from ELGA LabWater. The electrode substrate fluorine-doped tin oxide glass plates (FTO, resistivity 8−12 Ω/sq) and Ni foam (NF) were obtained from Sigma-Aldrich and Recemat BV, respectively. FTO glass was ultrasonically cleaned in acetone for about 10 min before use. NF was cleaned by ultrasonication for 10 minutes in 1 M HCl, followed by deionized water and acetone before use. The Hg/HgO reference electrode was purchased from CH Instruments, Inc. The Pt wire counter electrode (0.5 mm diameter × 230 mm length; A-002234) was purchased from BioLogic. Tetraethylene glycol (TEG, 99%) was degassed by freeze–pump–thaw cycles using liquid nitrogen and vacuum. All other chemicals were used as received without further purification.

1.2. Synthesis of Ni_2In_3 intermetallic nanoparticles

Using a Schlenk apparatus, $NiCl_2{\cdot}6H_2O$ (237 g/mol, 0.44 mmol, 103 mg), $InCl_3$ (221 g/mol, 0.56 mmol, 123 mg), and poly(2-ethyl-oxazoline) (M.W. 50,000, 0.6 g) were added to a 100 mL three-necked flask. The system was evacuated and backfilled with nitrogen three times to ensure an inert atmosphere. Next, 15 mL of degassed tetraethylene glycol (TEG) was added to the flask, and the flask was stirred at 600 rpm. The reaction mixture was then heated to 110 °C under vacuum for 1 h, with the thermocouple directly inserted into the reaction solution (Figure S1). Afterwards, the temperature was increased to 150 °C, and a freshly prepared solution of $NaBH_4$ (37.8 g/mol, 5.0 mmol, 190 mg) in 5 mL of degassed TEG was added dropwise over 15 min. Following the complete addition, the temperature was raised to 230 °C and maintained for 2 h. After heating was stopped, the solution was allowed to cool naturally to room temperature. The resulting product was then isolated by centrifugation at 8500 rpm for 5 min, washed three times with 60 mL ethanol and once with 40 mL acetone, and then dried in air to yield the desired black powder.

1.3. Synthesis of nickel or indium nanoparticles

To prepare the pure Ni and pure indium reference materials, the procedure outlined above was adapted by using only $NiCl_2 \cdot 6H_2O$ (237 g/mol, 1 mmol, 237 mg) for the Ni sample or only $InCl_3$ (221 g/mol, 1 mmol, 221 mg) for the indium sample, instead of both salts together.

1.4. Synthesis of $Fe_xNi_{1-x}OOH$ thin film on FTO

The $Fe_xNi_{1-x}OOH$/FTO was prepared according to the literature.[1] Nickel nitrate ($Ni(NO_3)_2 \cdot 6H_2O$, 291 g/mol, 1.90 mmol, 553 mg) and iron nitrate ($Fe(NO_3)_3$, 242 g/mol, 0.1 mmol, 24.2 mg) were dissolved in 20 mL of demineralized water to prepare a solution containing 0.095 mol/L nickel and 0.005 mol/L iron. Using this freshly prepared solution, without stirring, a film was cathodically deposited onto a 1 cm^2 FTO substrate in a two-electrode setup, with a platinum foil as the counter electrode. A current density of 1.4 mA/cm^2 was applied for 60 seconds. After deposition, the electrode was briefly rinsed in approximately 50 mL of high-purity water for five seconds to remove residual nitrate solution. Without allowing the electrode to dry, it was immediately used for the OER test.

1.5. Characterization

Powder X-ray Diffraction (PXRD). PXRD measurements were conducted using a Panalytical X'Pert PRO diffractometer, equipped with Bragg-Brentano geometry and Cu-Kα radiation.

Grazing Incidence X-ray Diffraction (GIXRD). GIXRD was performed using a PANalytical X'Pert Pro MPD X-ray diffractometer equipped with Cu-Kα radiation using grazing incidence geometry. An incidence angle of 0.5° was selected as it allows for optimal exploitation of the sample`s volume for the given layer thickness. The scan was carried out from 10 to 80°.

Scanning Electron Microscopy (SEM). To gain deeper insight into the surface morphology and structure of our samples, SEM was conducted using a Zeiss Gemini 500 microscope integrated with energy-dispersive X-ray (EDX) (EDAX, Apollo XPP). Data acquisition and analysis were performed using the EDAX software package.

Transmission Electron Microscope (TEM). The nanostructure of the samples was examined using an FEI Tecnai G2 20 S-TWIN transmission electron microscope (FEI Company, Eindhoven, Netherlands), equipped with a LaB6 source and operated at a 200 kV acceleration voltage. For post-electrocatalysis sample measurement, the catalyst films were scraped from the FTO electrode substrate, dispersed in acetone, and drop-cast onto a carbon-coated copper grid. Images were captured with a GATAN MS794 P CCD camera.

Inductively Coupled Plasma Optical Emission Spectroscopy (ICP-OES). ICP-OES was carried out on a Thermo Jarrell Ash Trace Scan analyzer. The samples were dissolved using 1 mL aqua regia and then diluted to 14 mL using high-purity H_2O with 18.2 MΩ cm. The aqua regia was freshly prepared by 1:3 HNO_3: HCl v/v (nitric acid, SUPRA-Qualität ROTIPURAN® Supra 69%, and hydrochloric acid, SUPRA-Qualität ROTIPURAN® Supra 30%).

X-ray Photoelectron Spectroscopy (XPS). XPS analysis was performed using a Kratos Axis Ultra spectrometer (Kratos Analytical Ltd., UK) equipped with a monochromatic Al-Kα X-ray source (hv = 1486.7 eV). Core-level spectra of Ni 2p, In 3d, Fe 2p, and O 1s were recorded using a pass energy of 20 eV and an energy step size of 0.1 eV. To enhance the signal-to-noise ratio, 100 scans were collected for the Fe 2p region due to its low concentration, whereas other elements required 25 scans. XPS data were analyzed using XPS Peak software. All binding energies were referenced to the adventitious carbon C 1s peak at 284.8 eV for charge correction. The Shirley background was subtracted from all spectra before peak fitting. The peaks were fitted using a Gaussian-Lorentzian (GL) function. For the deconvolution of all elements, constraints were applied to the peak positions and full-widths at half-maximum (FWHMs) where appropriate. Specifically, apart from the shake-up satellite peaks which possess inherently broad features, the FWHMs of all other fitted sub-peaks were strictly constrained to be less than 3.5 eV (typically falling within a reasonable range of 1.4–2.5 eV).

Raman spectroscopy. *Ex situ* and *in situ* Raman spectroscopy was performed using a Renishaw inVia confocal Raman spectrometer, with laser position and intensity calibrated using a standard Si wafer. *Ex situ* Raman measurements of dry samples were carried out using a 50× objective. For *in situ* measurements, a Leica water immersion objective (40×, 0.8 NA) was used, covered with a thin Teflon film to protect it from the corrosive electrolyte. *In situ* Raman measurements were conducted in a custom-built three-electrode Teflon cell, with activated samples deposited on a Pt sheet (Ni_2In_3/Pt-6M, Ni_2In_3/Pt-6M-Fe, Ni NP/Pt-6M, and Ni NP/Pt-6M-Fe) and immersed in 1 M KOH. A SP-200 potentiostat (BioLogic) controlled the applied potential, using a Pt-ring as the counter electrode and an Ag/AgCl electrode as the reference. Raman Spectra were collected in chronoamperometry (CA) mode at OCP, 1.25 V, 1.35 V, 1.40 V, 1.45 V, and 1.50 V vs. RHE. Before each measurement, the samples (Ni_2In_3/Pt-6M, Ni_2In_3/Pt-6M-Fe, Ni NP/Pt-6M, and Ni NP/Pt-6M-Fe) had been activated under their respective conditions (see Section 1.6), and the potential was held for 5 min at each step prior to spectral acquisition. At each potential, three different sample spots were measured. A 473 nm blue laser operating in edge mode with a circular spot was used as the excitation source at 10% of the

maximum power (~13 mW). Each spectrum (136–2267 cm^{-1} range) was obtained by averaging five accumulations, with a collection time of 10 s per accumulation. Raman spectra were analyzed using Fitky software.

X-ray Absorption Spectroscopy (XAS). Quasi-in-situ XAS, including X-ray absorption near edge structure (XANES) and extended X-ray absorption fine structure (EXAFS) measurements, were conducted at the KMC-3 beamline of BESSY II synchrotron (Helmholtz-Zentrum Berlin) using a closed-cycle helium cryostat (Oxford Instruments) maintained at 20 K, with incident beam energy selected by a Si(111) double-crystal monochromator.[2] Quasi-in-situ XAS measurements were performed on the samples of Ni_2In_3/FTO-p6M, Ni_2In_3/FTO-6M, Ni_2In_3/FTO-6M-Fe and Ni NP/FTO-6M-Fe after activation under respective conditions. These activated samples were freeze-quenched under argon flow with liquid N_2 (−196 °C) after chronoamperometry (CA) operation at target potentials (1.2 V_{RHE} and 1.6 V_{RHE}) for over 20 min, and stored in liquid N_2 until spectral acquisition to prevent structural relaxation as previously described.[2, 3] Ni and Fe K-edge spectra were acquired in fluorescence mode via a 13-element silicon-drift detector array (RaySpec), where K-edge energies were determined by the integral method. The raw spectra were processed with k^3-weighting for k-space simulations, and EXAFS fitting was implemented using phase functions derived from FEFF 8.4, followed by least-squares minimization (Levenberg-Marquardt algorithm) of the residual error between experimental Fourier transforms (1-3 Å reduced distance range) and simulated spectra, as detailed in prior studies.[2, 4] Further quantitative analysis of the coordination structure was performed by fitting the R-space of all samples using a model based on metal (oxy)hydroxides (Figure S38). In the layered structure formed by edge-sharing [MO_6] octahedra, the applied model considers three coordination shells: Ni–O_a, Ni–Ni, and Ni–O_b. For each shell, two distinct bond distances are included to account for the varied oxidation states of the transition metals. The respective coordination numbers (population) for the first, third, and fifth shells were constrained to be the same, indicating a higher Ni oxidation state with shorter bonds. Likewise, those for the second, fourth, and sixth shells were constrained to be the same, indicating a lower Ni oxidation state with longer bonds.[2] The fitting models, either including or excluding an additional Ni–In shell for the Ni_2In_3-derived samples, or a Ni–Ni shell for the Ni NP-derived sample, were systematically compared (Table S4-S7). Excluding the additional Ni–In or Ni–Ni coordination shell from the fitting model results in significantly higher R_f values, underscoring the importance of including these shells and confirming the reliability of our fitting approach. In our models, the sum of the coordination numbers of the first-sphere shells (Ni-O, Ni-O, Ni-In) were constrained to be six, which is reasonable and provided stable fits

with a minimum number of fit variables. Fits without this constraint resulted in first-sphere shell populations of 5.7-7.1 with similar R_f-values (not shown), but lacking any trends, and were thus discarded. Also, since the FT-spectra of the samples at 1.2 V_{RHE} are characterized by weak amplitudes of the metal-metal-peaks, which could either be caused by reduced crystallinity or shell-cancellation, additional fits were conducted in which the shell populations of the Ni-Ni shells were constrained to be half of those from the short Ni-O shells (not shown). These fits resulted in considerably higher R_f values (by up to 10%), making the shell cancellation effect more plausible than the reduced crystallinity. Since Fe is ubiquitous, we measured the XAS of bare FTO as a reference to enhance the reliability of our data. The Fe spectra of our samples appear significantly more oxidic compared to that of bare FTO. Despite the relatively low absolute fluorescence counts in both cases (approximately 280 counts for our samples vs. ~100 counts for bare FTO), the spectra still provide meaningful information (Figure S39). While bare FTO exhibits strong Fe–Fe bonding features, our samples predominantly display stronger Fe–O bonding characteristics. For the Fe K-edge EXAFS fitting, due to lower signal intensity compared to Ni and the absence of long-range structural order, the coordination numbers were not constrained, and all shell populations were freely refined.

1.6. Electrophoretic deposition (EPD) on substrates and electrochemical activation

The materials under investigation were electrophoretically deposited onto both NF and FTO substrates by applying a potential difference of 10 V in a mixture of iodine and acetone over a 1 × 1 cm^2 area.[5] For the standard deposition procedure, 15 mg of catalyst powder was dispersed in 5 ml of acetone, to which 2 mg of iodine was added. The solution was then sonicated for 30 minutes. Thin, uniform films were obtained by applying a potential of 10 V for 30 seconds while continuously stirring the solution at room temperature. The obtained samples were named Ni_2In_3/FTO and Ni_2In_3/NF.

Ni_2In_3/FTO was activated in KOH solutions of varying concentrations (1M, 2M, 3M, and 6M) through 60 cyclic voltammetry (CV) cycles between 1.1 and 1.7 V_{RHE} at a scan rate of 5 mV/s. After activation, these samples were labeled as Ni_2In_3/FTO-*n*M, where *n* represents the concentration. After Fe spiking to Ni_2In_3/FTO-6M-Fe (see Section 1.8), the sample was named as Ni_2In_3/FTO-6M-Fe. Similarly, Ni nanoparticles were deposited on FTO by EPD, named as Ni NP/FTO. After electrochemical activation in 6 M KOH and subsequent Fe spiking, the samples were referred to as Ni NP/FTO–6M and Ni NP/FTO–6M–Fe, respectively. For Raman measurements, the samples were also deposited on Pt foil. Similarly, they were denoted as Ni_2In_3/Pt-6M, Ni_2In_3/Pt-6M-Fe, Ni NP/Pt-6M, and Ni NP/Pt-6M-Fe.

1.7. Electrochemical measurements in a three-electrode cell

The electrochemical measurements were carried out using a standard three-electrode cell, consisting of a working, counter, and reference electrode, with 30 mL of 1.0 M aqueous KOH. A potentiostat (SP-200, BioLogic Science Instruments) controlled by the EC-Lab v10.20 software was employed for data acquisition. Fluorine-doped tin oxide (FTO) glass plates with thin films of the respective compounds served as the working electrode, while a Pt wire (0.5 mm diameter × 230 mm length, A-002234, BioLogic) acted as the counter electrode, and a Hg/HgO electrode (CH Instruments, Inc.) was used as the reference electrode. The prepared Ni_2In_3/FTO and Ni NP/FTO catalysts were activated in KOH solutions of varying concentrations (1M, 2M, 3M, and 6M) through 60 cyclic voltammetry (CV) cycles in the potential range of 1.1–1.7 V_{RHE}. The calibration of the Hg/HgO reference electrode was conducted following the reported experiment-based calibration procedure, using a commercial Gaskatel Hydroflex reversible hydrogen electrode (RHE).[6] Before calibration, the electrodes were immersed in 1 M KOH under Ar for at least 12 h to ensure stable and reliable readings. The measurement was repeated four times with independently prepared electrolyte solutions to confirm reproducibility. The open-circuit potential (OCP) between the Hg/HgO electrode and the Hydroflex RHE was recorded for 60 s after stabilization. The calibration potential of the Hg/HgO electrode versus RHE in 1 M KOH was experimentally determined to be 0.931 V, which was used to convert all measured potentials to RHE in this work, using the equation:

$$E_{RHE} = E_{measured} + 0.931\text{V} - iR \qquad (1)$$

Prior to each electrochemical experiment, the uncompensated solution resistance (R_u) was determined by an impedance point measurement at 1.168 V_{RHE}, 100 MHz, 10 mV amplitude. Five data points were acquired at 0.1 s intervals and averaged. The R_u values ranged from 10.7 to 12.9 Ω for samples deposited on FTO. The R_u values ranged from 0.9 to 1.1 Ω for samples deposited on Ni foam. The impedance point measurement took less than one second, after which the cyclic voltammetry started automatically. During the measurements, 90% of the R_u was compensated in real time (on-the-fly *iR* compensation) for CV measurements. 90% of the R_u was manually compensated for CP measurements, since the potentiostat was not capable of real-time *iR* compensation for CP. An additional 10% manual *iR* compensation was applied during data processing for a more accurate evaluation of OER activity, as shown in Figure 3a, Figure 5a and Figure 6a. Each sample was tested at least three times. The error bars for the overpotentials were provided based on the results from three independently prepared films on FTO, derived from the same synthesis batch. Electrochemical impedance spectroscopy (EIS)

was recorded at 1.56 V_{RHE} to obtain the Nyquist plots. The amplitude of the sinusoidal wave was determined in a frequency range of 100 kHz to 1 mHz.

The steady-state Tafel slopes were determined from potentiostatic measurements with stepwise potential increments of 15 mV. At each potential, the current was recorded for 180 s under constant potential, and the average current values were used to construct the Tafel plots. The Tafel slope (**b**) was obtained according to the following Tafel equation. The error bars for the Tafel slopes were provided based on the results from three independently prepared films on FTO, derived from the same synthesis batch.

$$\eta = b \log j + a \qquad (2)$$

where η is the overpotential, j is the current density, and a is a constant related to the logarithm of the exchange current density.

To evaluate the intrinsic activity, the corresponding normalized CV curves were plotted. First, the integration of the Ni(II/III/IV) reduction peaks within 1.1–1.5 V_{RHE} was used to estimate the quantity of Ni redox-active sites. The number of moles of electrons transferred during the reaction was calculated from the integrated area of the reduction peak according to Equation (3). Finally, the normalized CV curves were obtained by dividing the current density by the number of moles of electrons transferred.

$$[e^-] = \frac{1}{\text{scan rate} \times F} \times \int_{V_1}^{V_2} I(E)dE \qquad (3)$$

Scan rate= 5 mV/s, F=96485 s·A/mol. V_1 and V_2 were defined as the potentials at which the CV current first deviated from and subsequently returned to 0 mA cm^{-2}, respectively. The integrated area between the CV curve and the 0 mA cm^{-2} baseline within this potential range—including both anodic and cathodic currents—was calculated to obtain the charge associated with the Ni(II/III/IV) redox process.

1.8. Fe spiking

In order to maximize the promotional effect of Fe incorporation, Fe spiking was further carried out on the activated Ni_2In_3/FTO-6M and Ni NP/FTO-6M samples through 30 CV cycles in the potential range of 1.1–1.6 V_{RHE} in 40 mL of 1M KOH with 0.5 mM $FeSO_4$ xH_2O (5.6 mg). Small amounts of yellow precipitate were observed, indicating that the KOH solution was saturated with Fe species.

1.9. Electrochemical measurements in AEMWE cell

The Ni_2In_3/NF-6M-Fe catalyst with the Ni_2In_3 loading of 1.6 mg cm^{-2} as anode was assessed in an anion exchange membrane water electrolysers (AEMWE) cell with Ni_4Mo/MoO_2@NF as cathode under industrially relevant conditions. The electrode area was 5 cm^2. Ni_4Mo/MoO_2@NF was prepared based on the previous report.[7] The used membrane was PiperlON 80 µm. A potentiostat (SP-200, BioLogic Science Instruments) controlled by the EC-Lab v10.20 software was employed for data acquisition. Prior to each electrochemical experiment, the uncompensated solution resistance (R_u) was determined by an impedance point measurement at 1.168 V_{RHE}, 100 MHz, 10 mV amplitude. Five data points were acquired at 0.1 s intervals and averaged. The R_u values ranged from 0.07 to 0.11 Ω for samples deposited on 5 cm^2 Ni foam. The impedance point measurement took less than one second, after which the cyclic voltammetry started automatically. During the measurements, 90% of the R_u was compensated in real time (on-the-fly iR compensation) for CV. 90% of the R_u was manually compensated for CP measurements, since the potentiostat was not capable of real-time iR compensation for CP. At first, fast-scanning CV with a scan rate of 5 mV s^{-1} and step-wise chronoamperometry (CA) at different temperatures were measured in 1M KOH at 25 and 80 ^{o}C, respectively. The error bars were calculated based on the fluctuations (noise) in the CA data. At each potential, the average of the maximum and minimum current density values is taken as the current density shown in the figure, and the difference between the maximum and minimum values is used as the length of the error bar. The stability was measured via chronopotentiometry (CP) at 1 A cm^{-2} at 80 ^{o}C. To maintain the dynamic balance of Fe species at the interface during the long-term stability test, a small amount of Fe-containing electrolyte was introduced into the electrolyte reservoir after approximately 48 h of operation. A $FeSO_4 \cdot xH_2O$ solution with a nominal concentration of 0.5 mM was freshly prepared in 1M KOH before use. A small amount of yellow precipitate was observed, indicating that the solution was saturated with Fe species. Subsequently, 5 mL of the saturated Fe solution was added to the electrolyte reservoir with about 180 mL of 1M KOH solution. The Fe-containing electrolyte was added only once during the entire test, and no further Fe replenishment was performed. The introduced Fe species were intended to compensate for Fe loss from the catalyst surface during prolonged OER operation and to facilitate a dynamic equilibrium between Fe dissolution and redeposition, thus helping to preserve a high number of the dynamically stable interfacial Fe species.[8]

1.10. Computational methods

All spin-polarized density functional theory (DFT) calculations were performed using the Vienna ab initio simulation package (VASP) code based on the projected-augmented-wave

(PAW) method [9]. The electron exchange and correlation energy were described by the Perdew-Burke-Ernzerhof (PBE) functional of the generalized gradient approximation (GGA) [10]. The cut-off energy for the plane wave was set to 500 eV. The force criterion and energy for convergence of the electron density were set to 0.05 eV $Å^{-1}$ and 10^{-5} eV, respectively. The van der Waals interactions between adsorbates and the surface were described by implementing DFT-D3 corrections, as proposed by Grimme [11-12]. The DFT + U method was employed to correct the localized 3d electrons with the Hubbard parameters of 5.5 and 4.3 eV for Ni and Fe, respectively [13]. The k-point grids were set as 3 × 3 × 1 for geometry optimization and 9 × 9 × 1 for electronic structure computations to sample the first Brillouin zone.

2. Figures and Tables

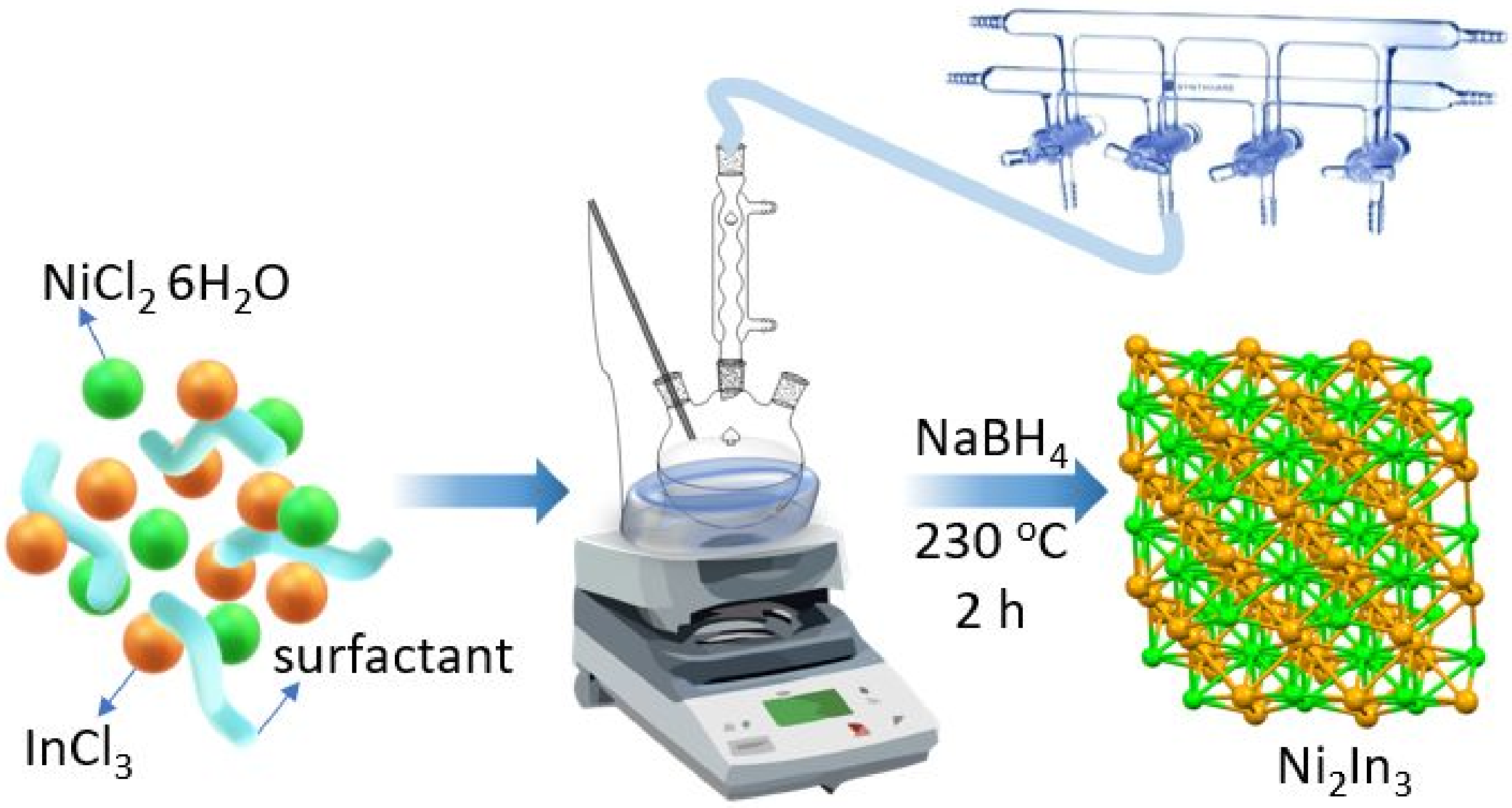

Figure S1. Synthesis schematic of intermetallic Ni_2In_3 nanoparticles. In brief, $NiCl_2 \cdot 6H_2O$, $InCl_3$, and poly(2-ethyl-oxazoline) as a surfactant in tetraethylene glycol were reduced by $NaBH_4$ at 230 ^oC for 2 h under an inert atmosphere.

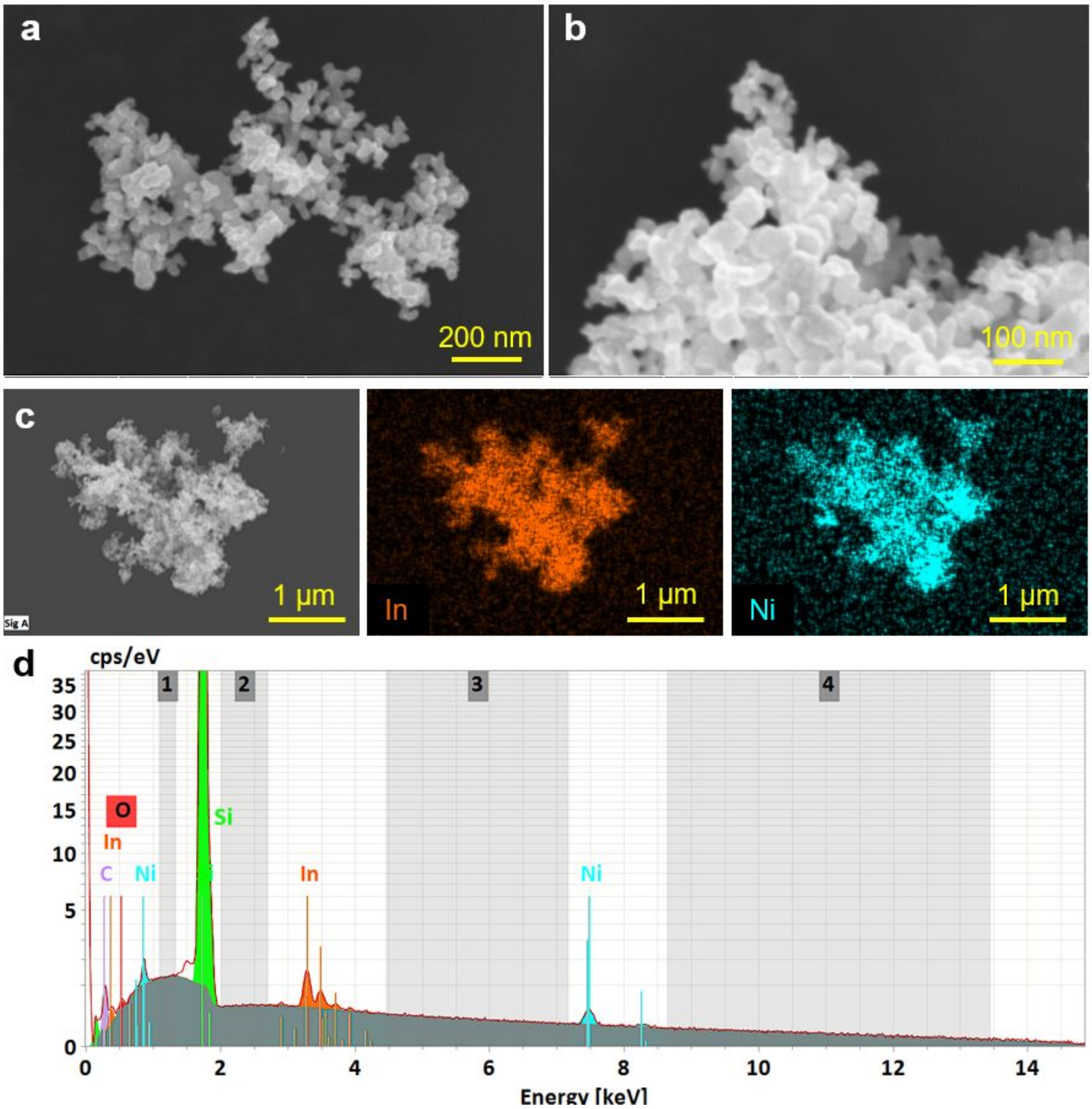

Figure S2. (a-b) SEM images, (c) elemental mapping, and (d) EDX of the as-prepared Ni_2In_3 powder on a silicon wafer. The results reveal that the particles are small and uniformly distributed, with a homogeneous spatial distribution of Ni and In. The EDX spectra confirm the presence of Ni and In, and the Si signal originates from the silicon wafer. A small C signal may arise from surfactant residues, while O is likely due to partial surface oxidation of the particles.

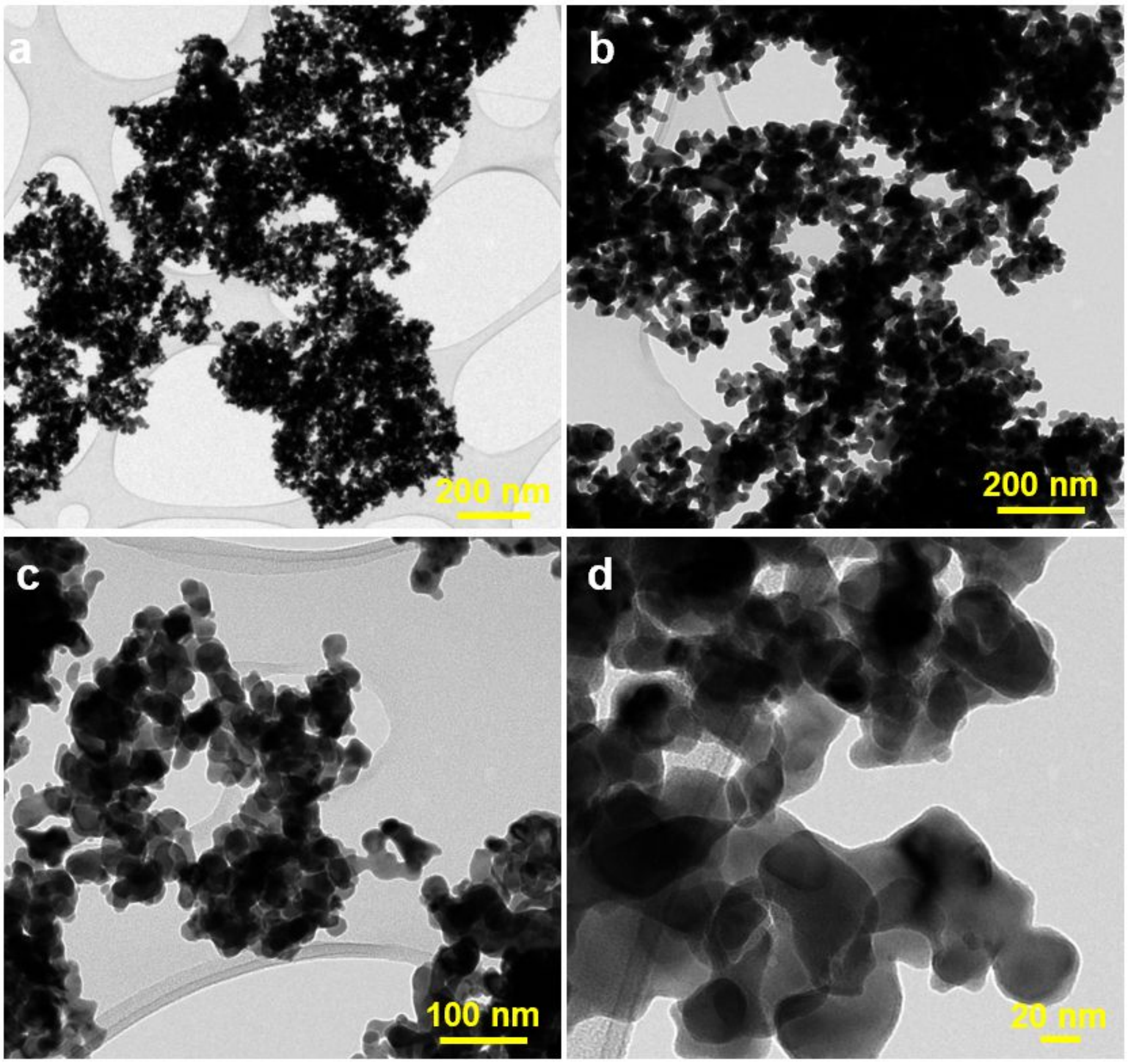

Figure S3. (a-d) TEM images of the as-prepared Ni_2In_3 powders at different magnifications. The TEM results further confirm that the nanoparticles are highly uniform, with sizes ranging from approximately 10 to 50 nm.

a

Shell	*N*	Error	*R* [Å]	Error [Å]	σ [Å]
Ni-In	8		2.62	0.01	0.087
Ni-Ni	3		2.95	0.02	0.102

R_f=26.3

Shell	*N*	Error	*R* [Å]	Error [Å]	σ [Å]
Ni-In	2.8	0.3	2.60	0.01	0.050
Ni-Ni	2.7	0.3	2.89	0.01	0.050

R_f=17.9

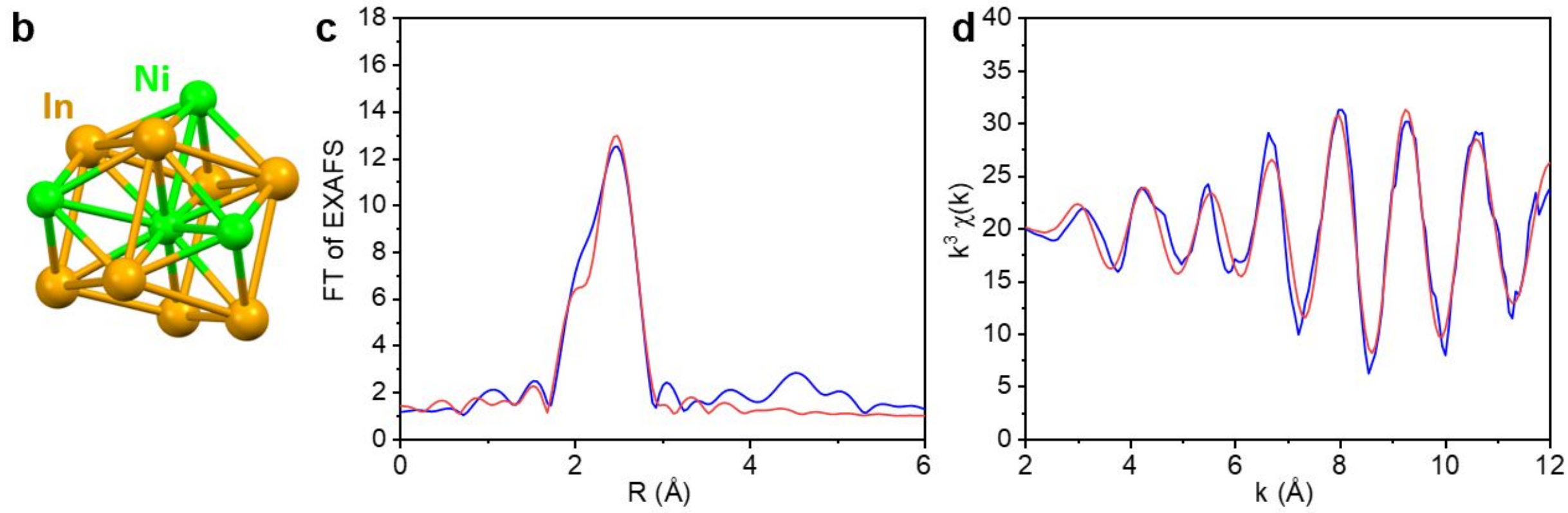

Figure S4. Ni K-edge EXAFS and fitting of the as-prepared Ni_2In_3 powders. (a) Table with parameters for simulations of the EXAFS spectra. The fit-range was 1-3 Å. *N* is the coordination number (or shell population) of the shell at distance *R*, *σ* is the Debye–Waller factor, and R_f represents the fitting error in %. The amplitude reduction factor S_0^2 was set to 0.85. Two fits were performed: one using the expected coordination number (b) and the other employing identical Debye–Waller factors (0.05) for comparison with other fits. The former got a high R_f value, whereas the latter provided a more reasonable result. (b) According to the crystal structure of Ni_2In_3 (PDF No. 65–7718), each Ni atom coordinates by eight indium atoms at bond lengths of 2.62–2.77 Å from **Figure 1b**, and the Ni–Ni coordination number is 3 at a distance of 2.94 Å. (c) Fourier transformed (FT) EXAFS spectra fitted in R space and (d) k^3-weighted EXAFS spectra from the latter fit. The blue and red lines represent the experimental and fitting values, respectively. From the latter fit, the Ni–In coordination number with a distance of 2.60 Å is 2.8. This low apparent value may arise from shell cancellation effects: multiple nearly identical scattering paths produce slight phase differences that lead to destructive interference and a reduced signal amplitude, similar to the case observed for $CoIn_3$.[14] The Ni–Ni coordination number is 2.8 at a distance of 2.89 Å, closely matching the theoretical value of 3.

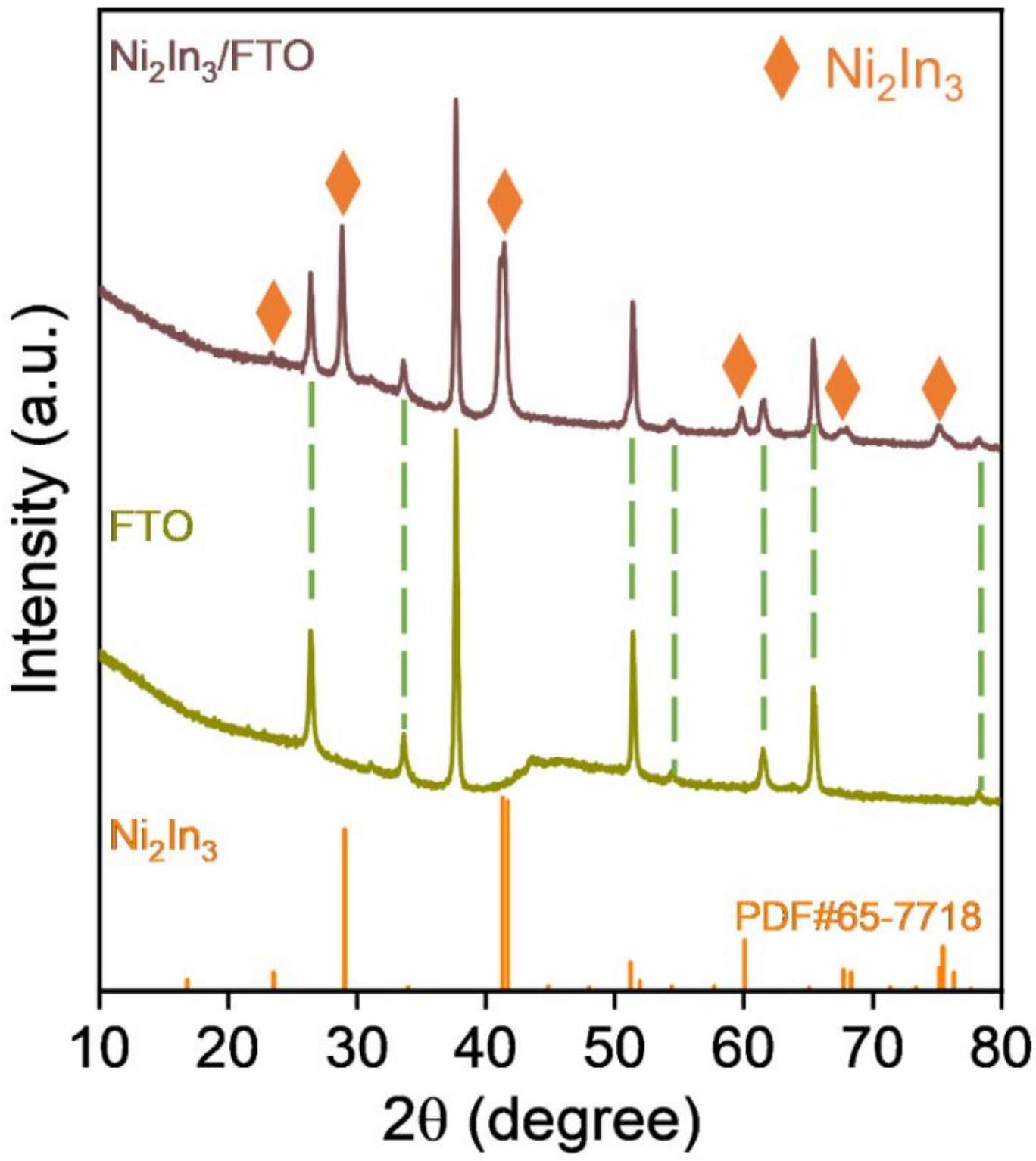

Figure S5. Powder XRD of blank FTO and Ni_2In_3/FTO after electrophoretic deposition with a simulated pattern of Ni_2In_3 (PDF No. 65–7718). There are only diffraction peaks corresponding to Ni_2In_3 and the FTO substrate observed, indicating a retained pure phase.

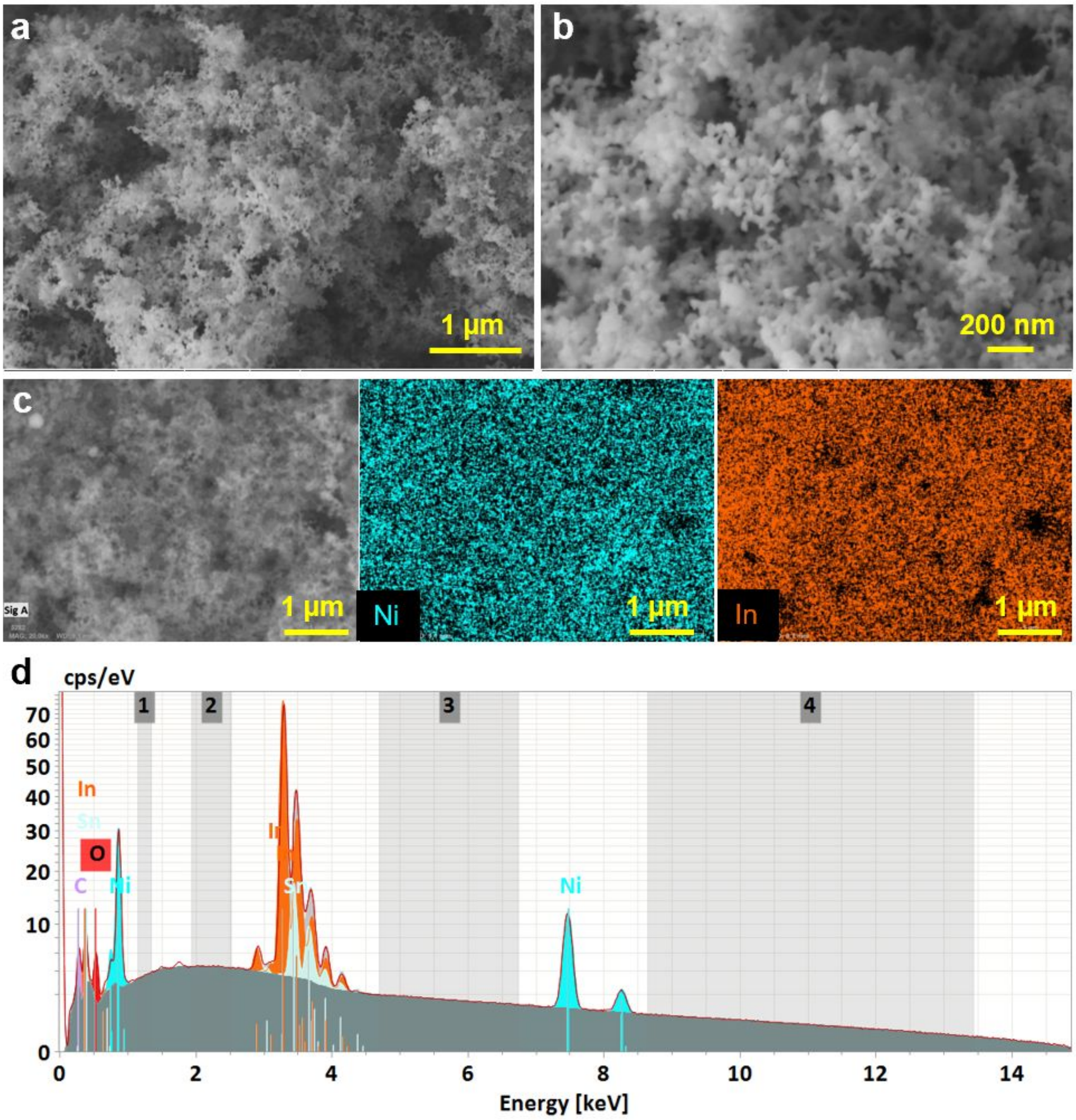

Figure S6. (a-b) SEM images, (c) elemental mapping, and (d) EDX of Ni_2In_3/FTO. The results reveal that the particles show no obvious change after EPD, with a homogeneous spatial distribution of Ni and In. The EDX spectra confirm the presence of Ni and In, while the Sn signal originates from the FTO substrate. A small C signal may arise from surfactant residues or contamination by carbon-containing compounds, whereas O is likely due to partial surface oxidation of the particles.

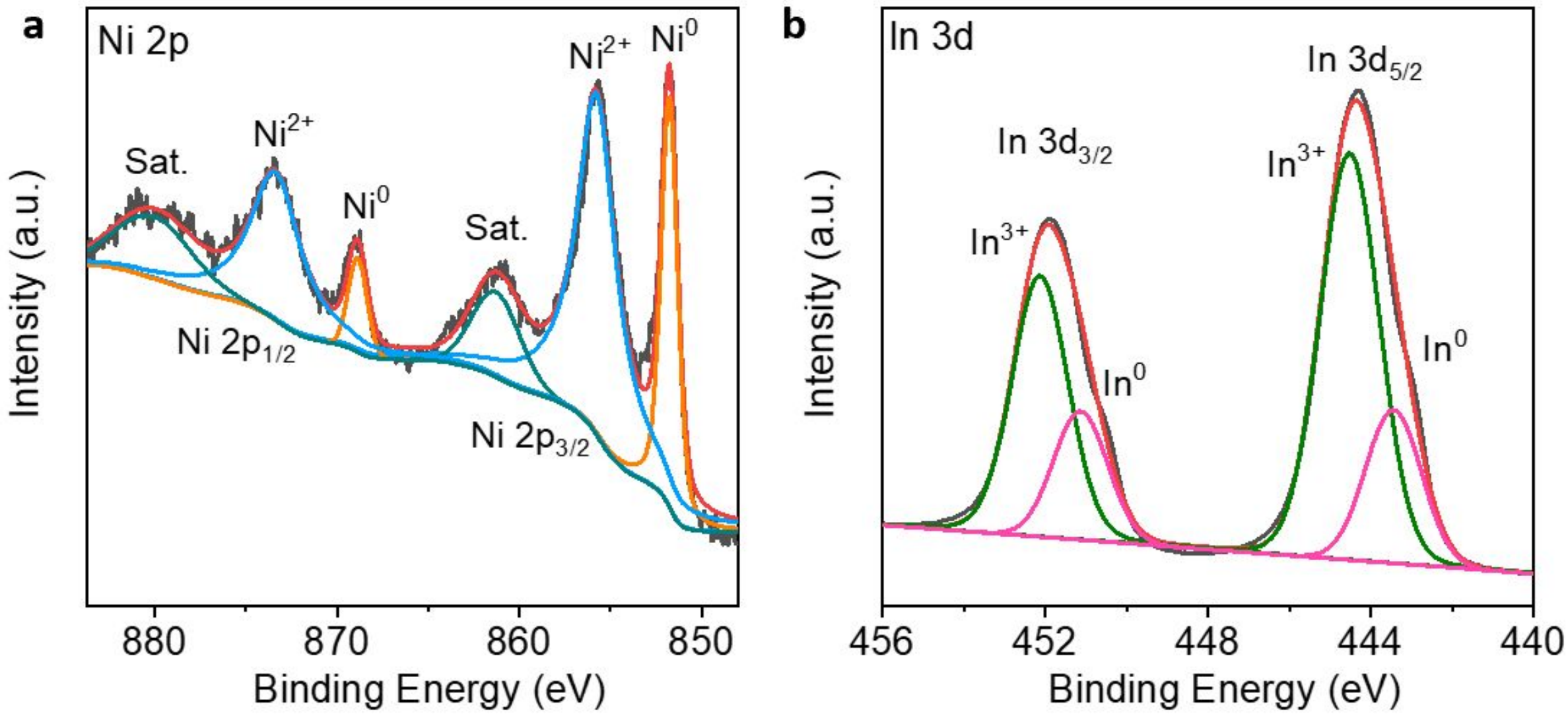

Figure S7. XPS of Ni_2In_3/FTO. (a) The Ni 2p XPS exhibits binding energies at 851.7 eV, 855.8 eV, and 861.5 eV, corresponding to $2p_{3/2}$ of metallic Ni^0, Ni^{2+}, and the satellite peak, respectively.[15] (b) The peaks at 443.4 eV and 444.5 eV correspond to the In $3d_{5/2}$ of metallic In^0 and In^{3+}, respectively.[16] The presence of oxidized species is attributed to surface oxidation upon exposure to air, which is a common phenomenon.[17] The high proportion of oxidized states is due to the surface sensitivity of XPS.

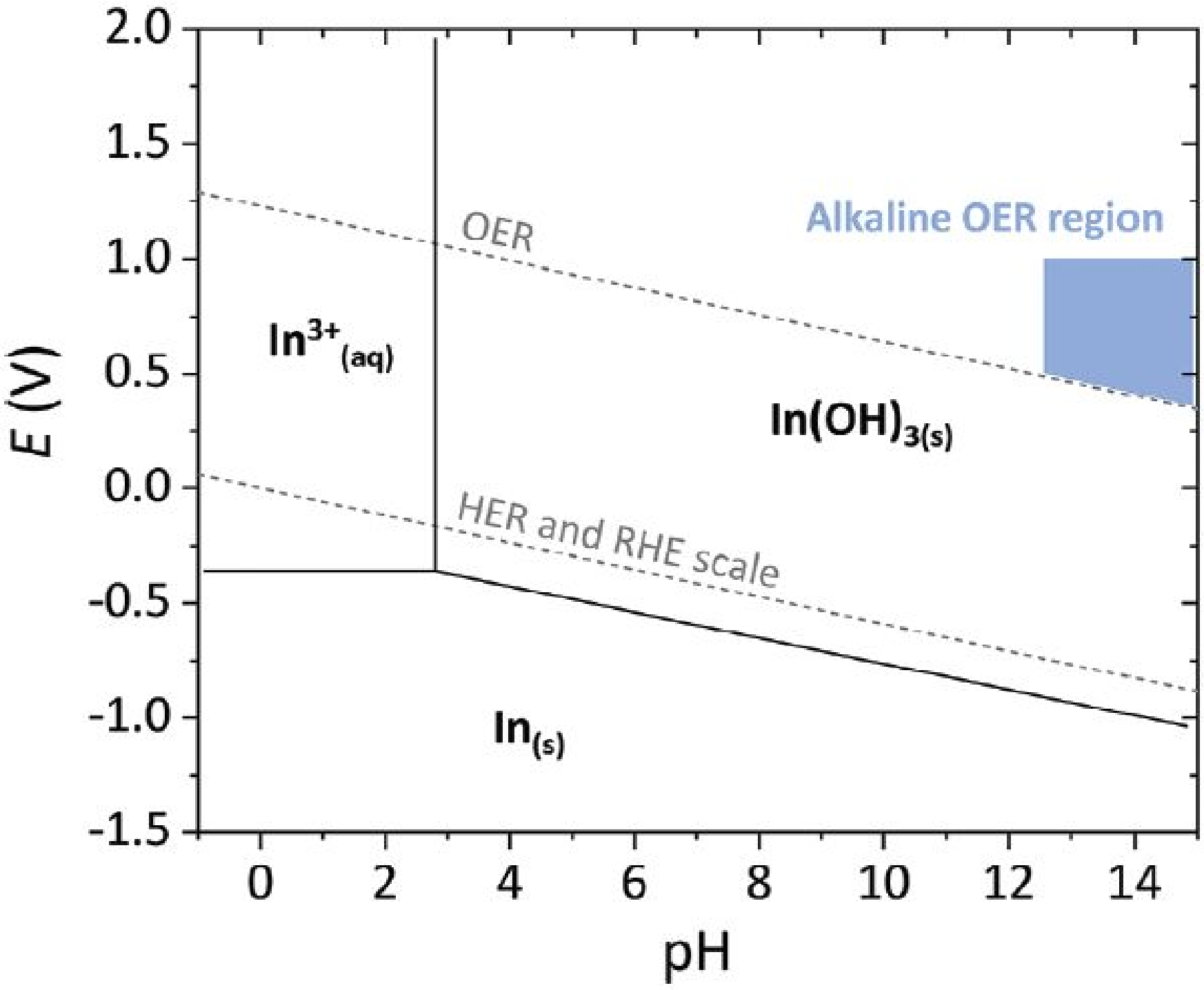

Figure S8. The Pourbaix diagram of indium (25 °C) redrawn from the book[18]: The aqueous chemistry of the elements.[14] The concentrations of soluble species are 0.1 mol/L. Soluble species and most solids are hydrated.

The Pourbaix diagram suggests that indium hydroxide is stable and thus non-leaching in the OER region, although it is constructed based on a relatively high concentration of 0.1 mol/L. Moreover, indium indeed does not leach as readily as other p-block elements in 1 M KOH, as evidenced in the case of $CoIn_3$, where activation primarily leads to phase separation, forming CoOOH and $In(OH)_3$. This gives the general impression that indium remains stable in the OER region in the form of $In(OH)_3$ and is difficult to leach. However, in our study, significant indium leaching was observed from Ni_2In_3 powders in concentrated KOH. Specifically, the indium content decreased by 80% after activation in 6M KOH. Although the amphoteric nature of $In(OH)_3$ remains under debate, indium may undergo leaching into the electrolyte in the form of $In(OH)_4^-$. The calculated dissolved indium concentration (about 3 x 10^{-5} mol/L) does not exceed 0.1 mol/L, and does not contradict the Pourbaix diagram, but it challenges our conventional expectations, hence the term "unexpected indium leaching." Its leaching behavior still offers valuable insights. This leaching behavior provides valuable insights into the chemical stability of indium under alkaline OER conditions.

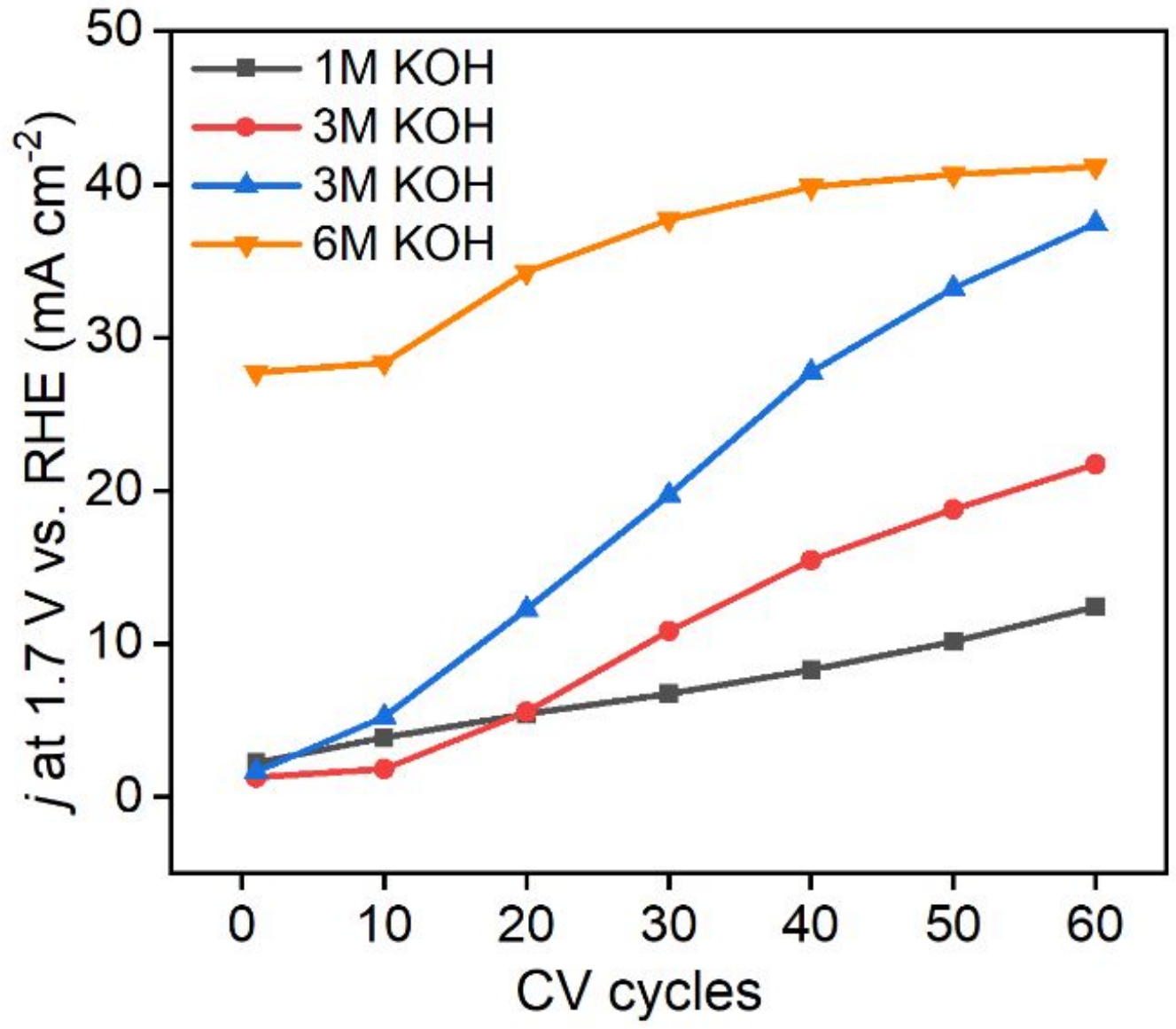

Figure S9. The current density at 1.7 V vs. RHE as a function of CV cycle number from the activation curves under different KOH concentrations in Figure 2a. For 6 M KOH, the current density at 1.7 V vs. RHE increases rapidly during the initial cycles and becomes significantly slower after approximately 40 cycles. Only a minimal change of 1.2% is observed between the 50th and 60th cycles, indicating that the reconstruction approaches a quasi-stable state after 60 cycles. Therefore, 60 cycles were chosen as a practical and consistent activation condition for the entire study. The samples obtained after 60 CV cycles represent reconstruction states generated under a unified activation protocol rather than the fully reconstructed equilibrium states for all electrolyte concentrations.

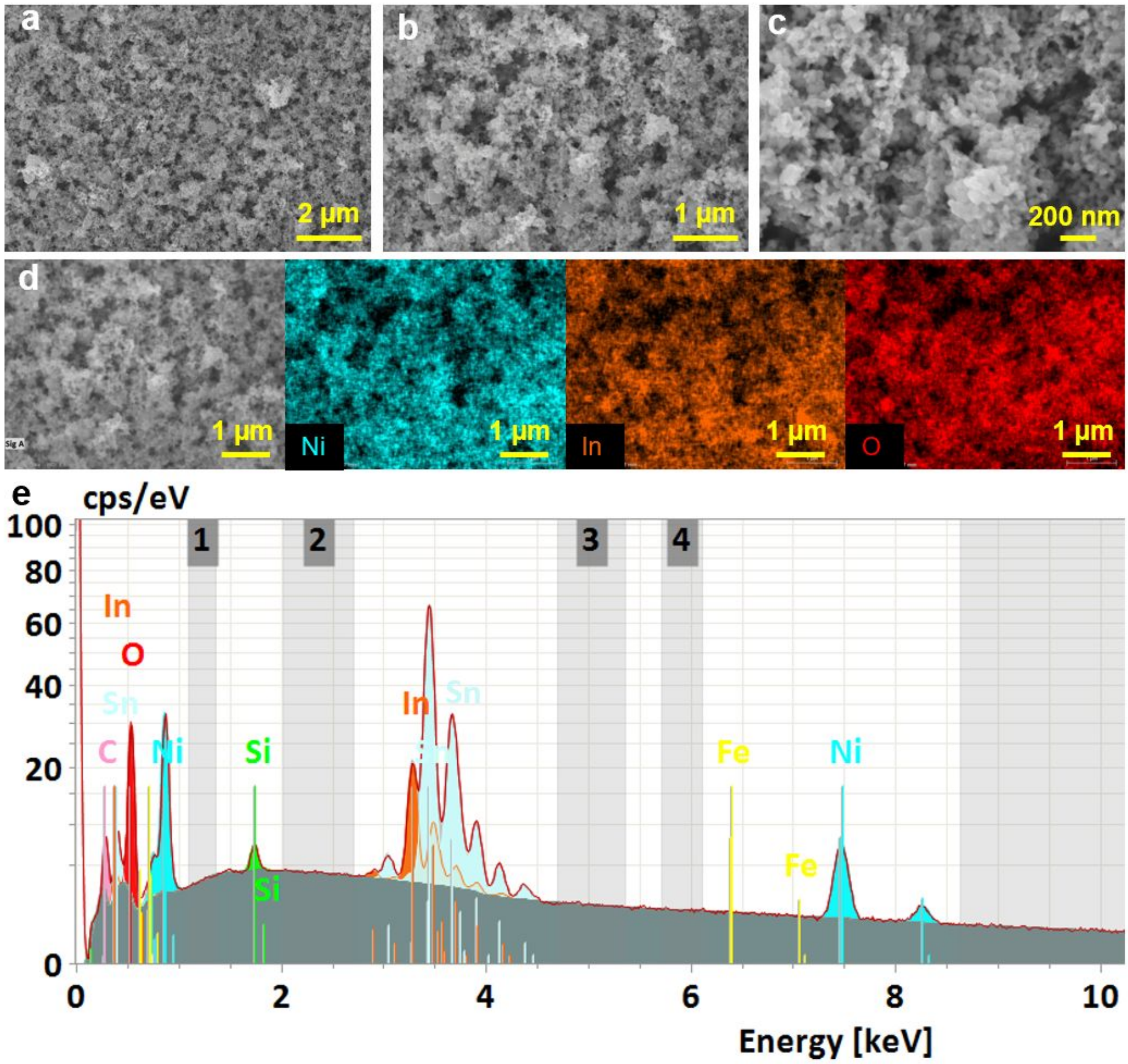

Figure S10. (a-c) SEM images, (d) elemental mapping, and (e) EDX of Ni_2In_3/FTO-1M. The results reveal that Ni_2In_3/FTO-1M exhibits a homogeneous spatial distribution of Ni, In, and O. The EDX spectra confirm the presence of Ni, In, and O, while the Sn signal originates from the FTO substrate and the Si signal from the silicon wafer. A small C signal may arise from surfactant residues or contamination by carbon-containing compounds. No obvious Fe signal peak was observed.

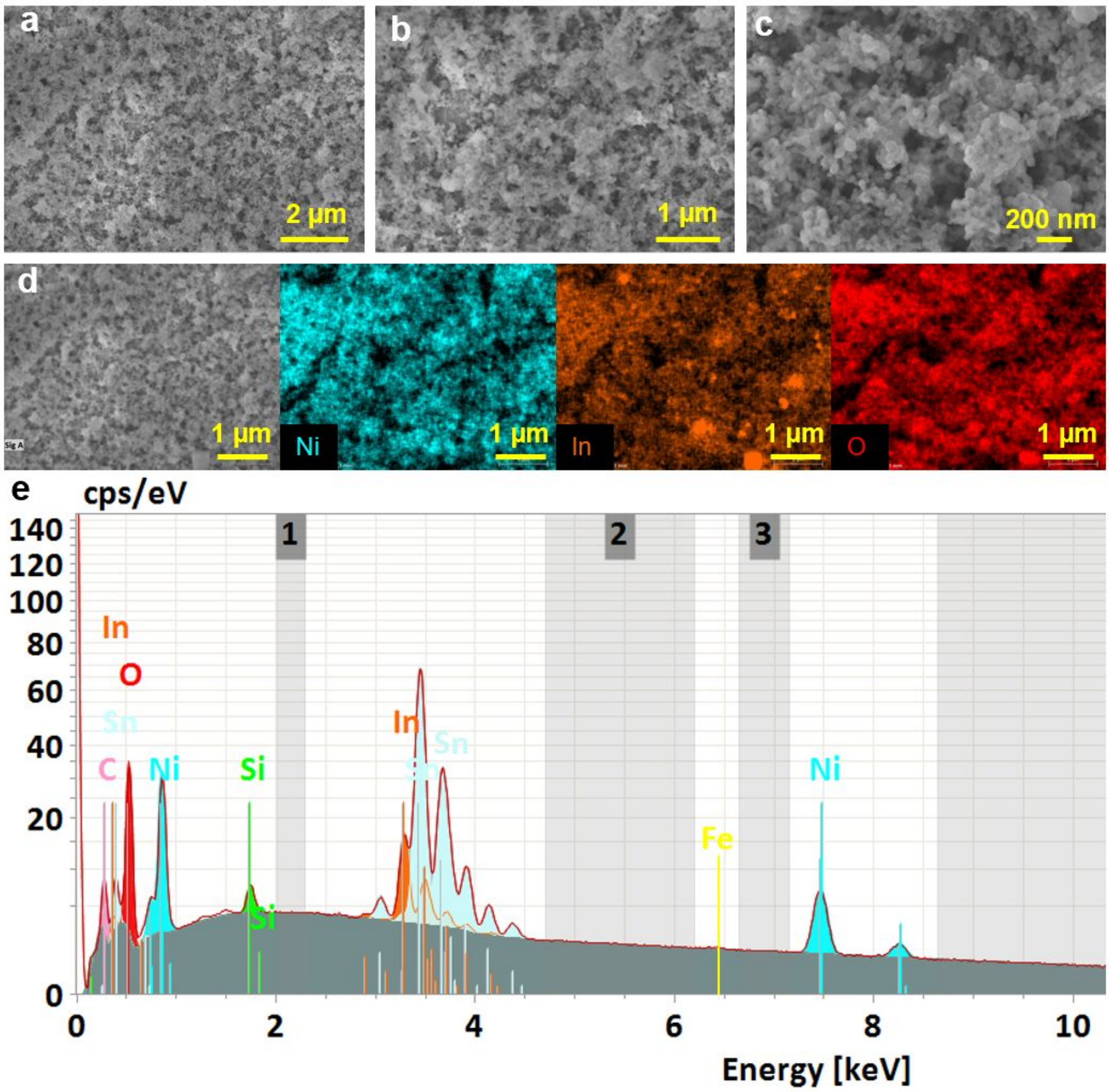

Figure S11. (a-c) SEM images, (d) elemental mapping, and (e) EDX of Ni_2In_3/FTO-2M. The results reveal that Ni_2In_3/FTO-2M exhibits a homogeneous spatial distribution of Ni, In, and O, with partial aggregation of In, indicating phase separation and the formation of $In(OH)_3$. The EDX spectra confirm the presence of Ni, In, and O, while the Sn signal originates from the FTO substrate and the Si signal from the silicon wafer. A small C signal may arise from surfactant residues or contamination by carbon-containing compounds. No obvious Fe signal peak was observed.

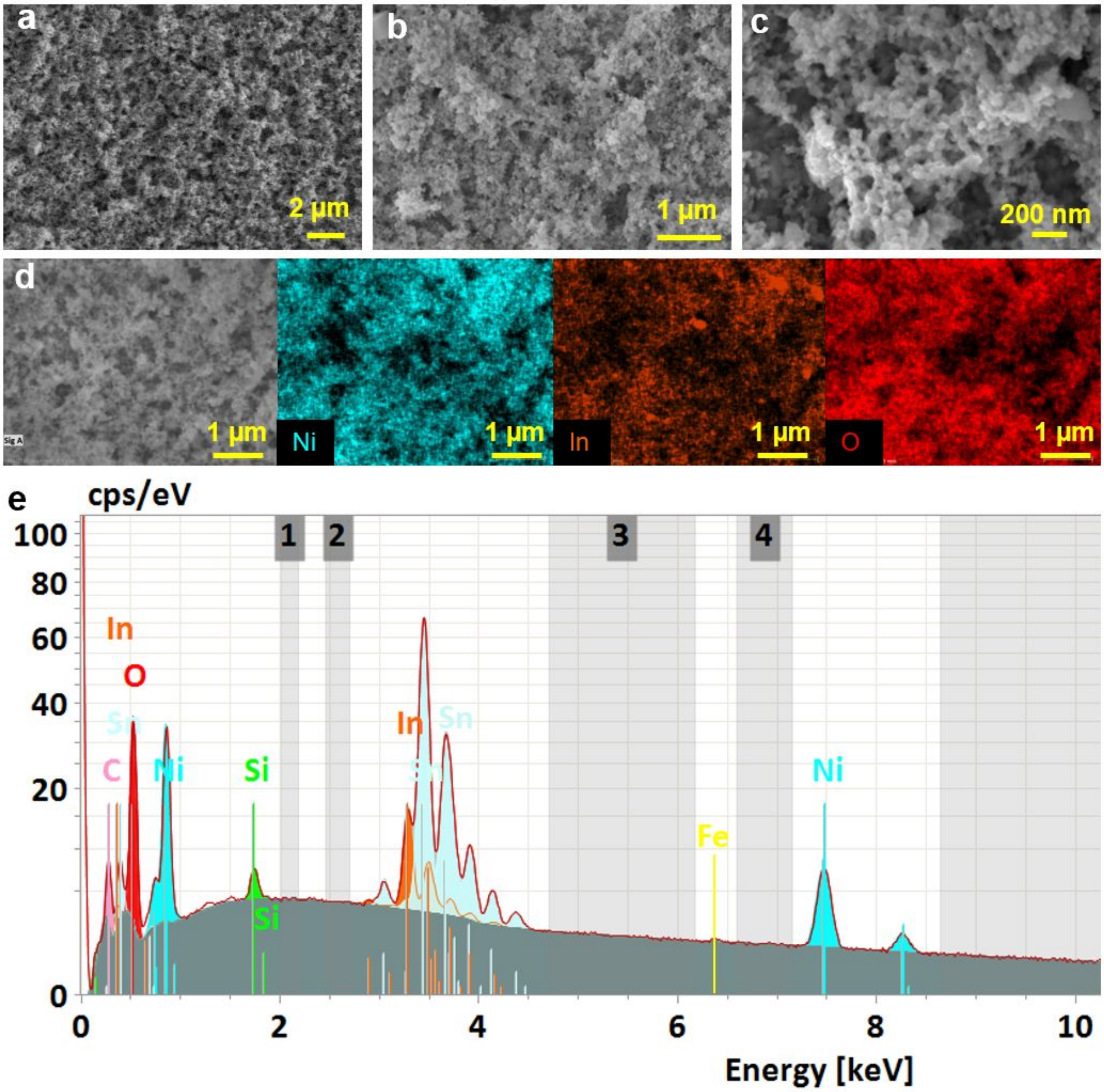

Figure S12. (a-c) SEM images, (d) elemental mapping, and (e) EDX of Ni_2In_3/FTO-3M. The EDX spectra confirm the presence of Ni, In, and O, while the Sn signal originates from the FTO substrate and the Si signal from the silicon wafer. A small C signal may arise from surfactant residues or contamination by carbon-containing compounds. A minor Fe signal peak was also observed.

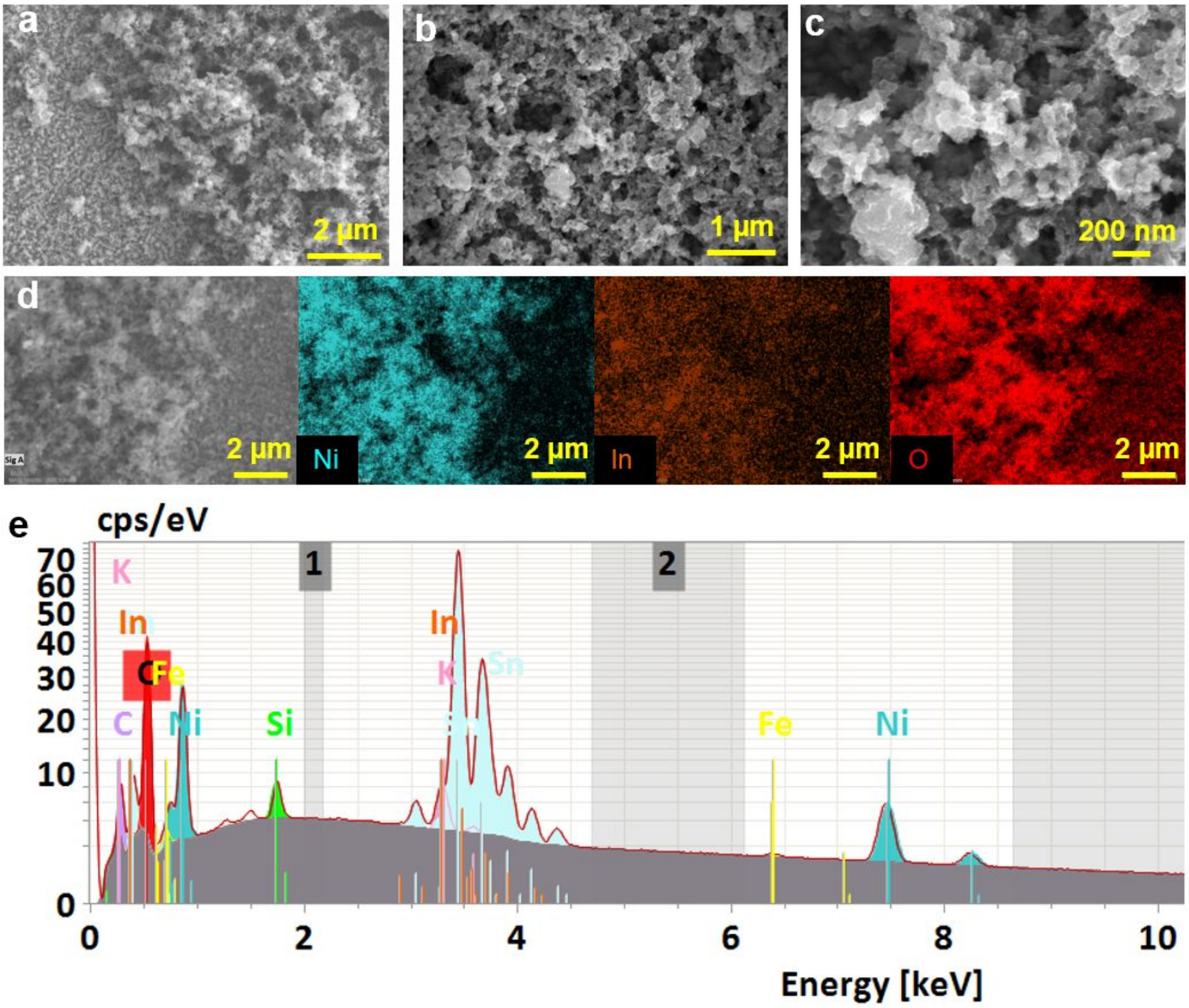

Figure S13. (a-c) SEM images, (d) elemental mapping, and (e) EDX of Ni_2In_3/FTO-6M. The EDX spectra confirm the presence of Ni, In, and O, with a significantly reduced In content. The Sn signal originates from the FTO substrate, and the Si signal from the silicon wafer. A small C signal may arise from surfactant residues or contamination by carbon-containing compounds. A small Fe signal peak was also observed.

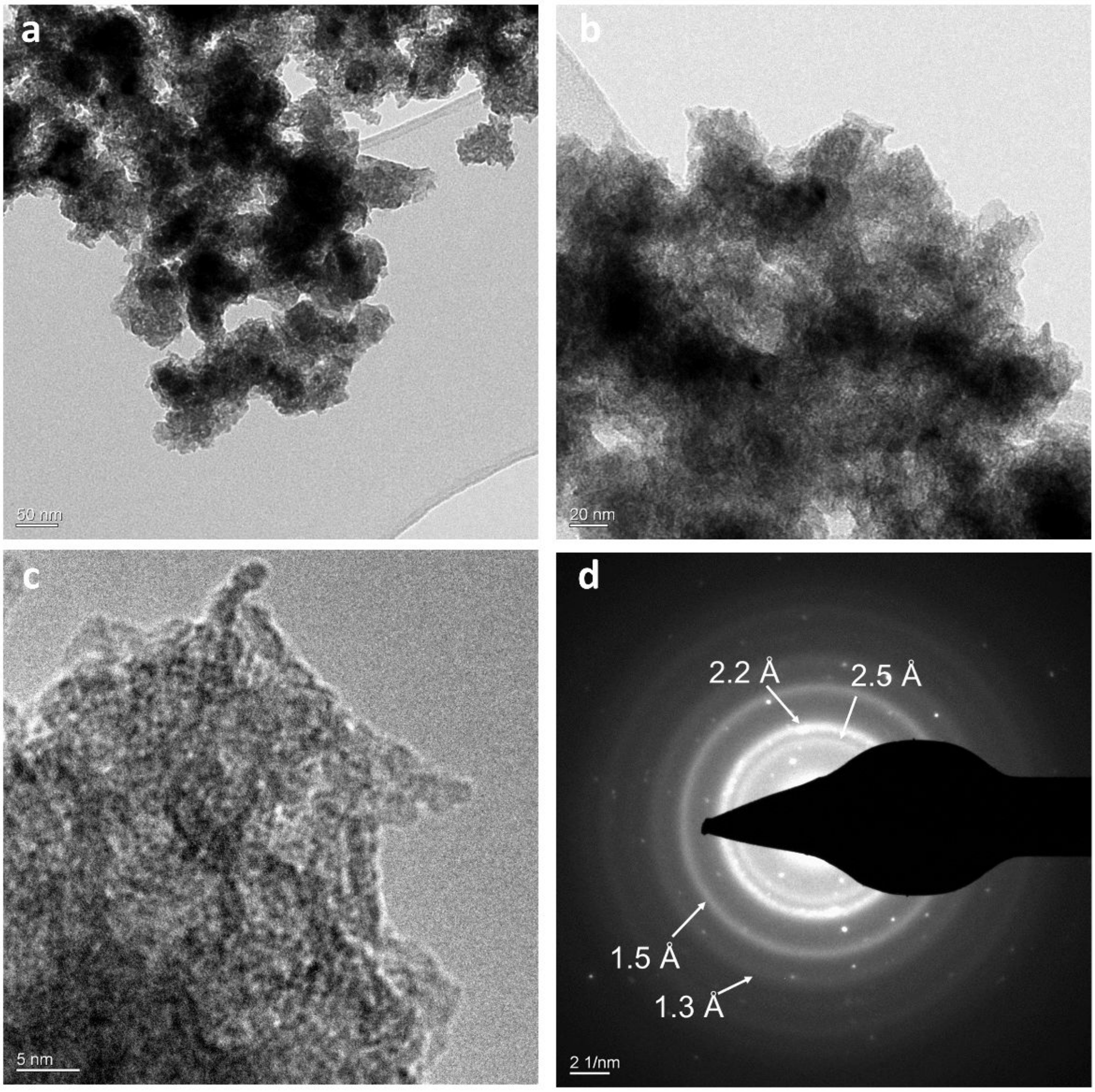

Figure S14. The (a-c) TEM images and (d) the corresponding SAED pattern of Ni_2In_3/FTO-6M. The SAED diffraction rings with d-spacings of approximately 2.5, 2.2, 1.5, and 1.3 Å are consistent with layered Ni (oxy)hydroxides. Owing to the highly similar crystal structures and diffraction features of NiOOH and $Ni(OH)_2$, as well as the limited resolution and broad diffraction rings in SAED, these phases cannot be reliably distinguished. Therefore, the observed phase is conservatively denoted as Ni (oxy)hydroxides.

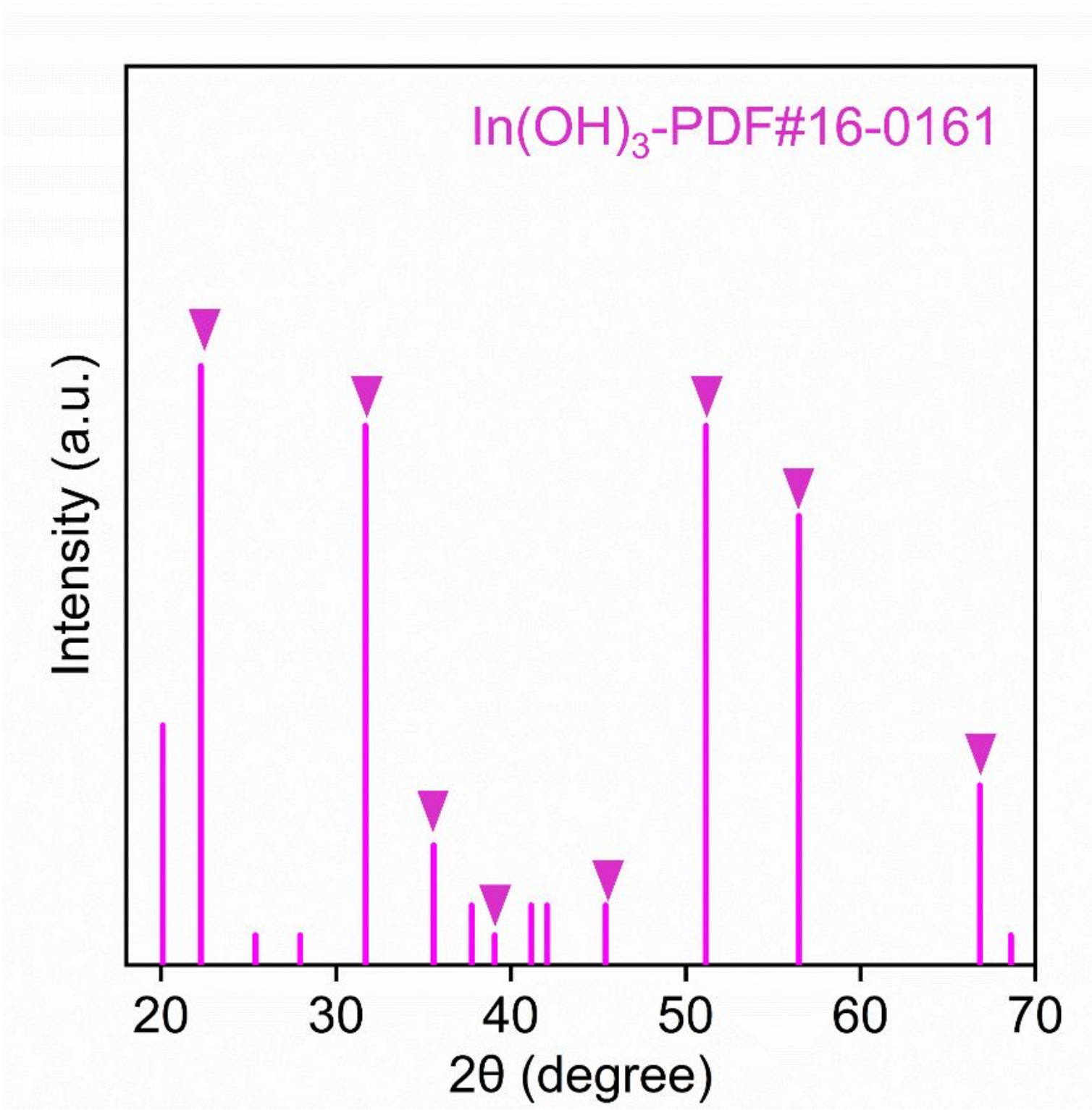

Figure S15. The simulated pattern of $In(OH)_3$ (PDF No.16-0161). The marked peaks can be observed in Figure 2h in the main text.

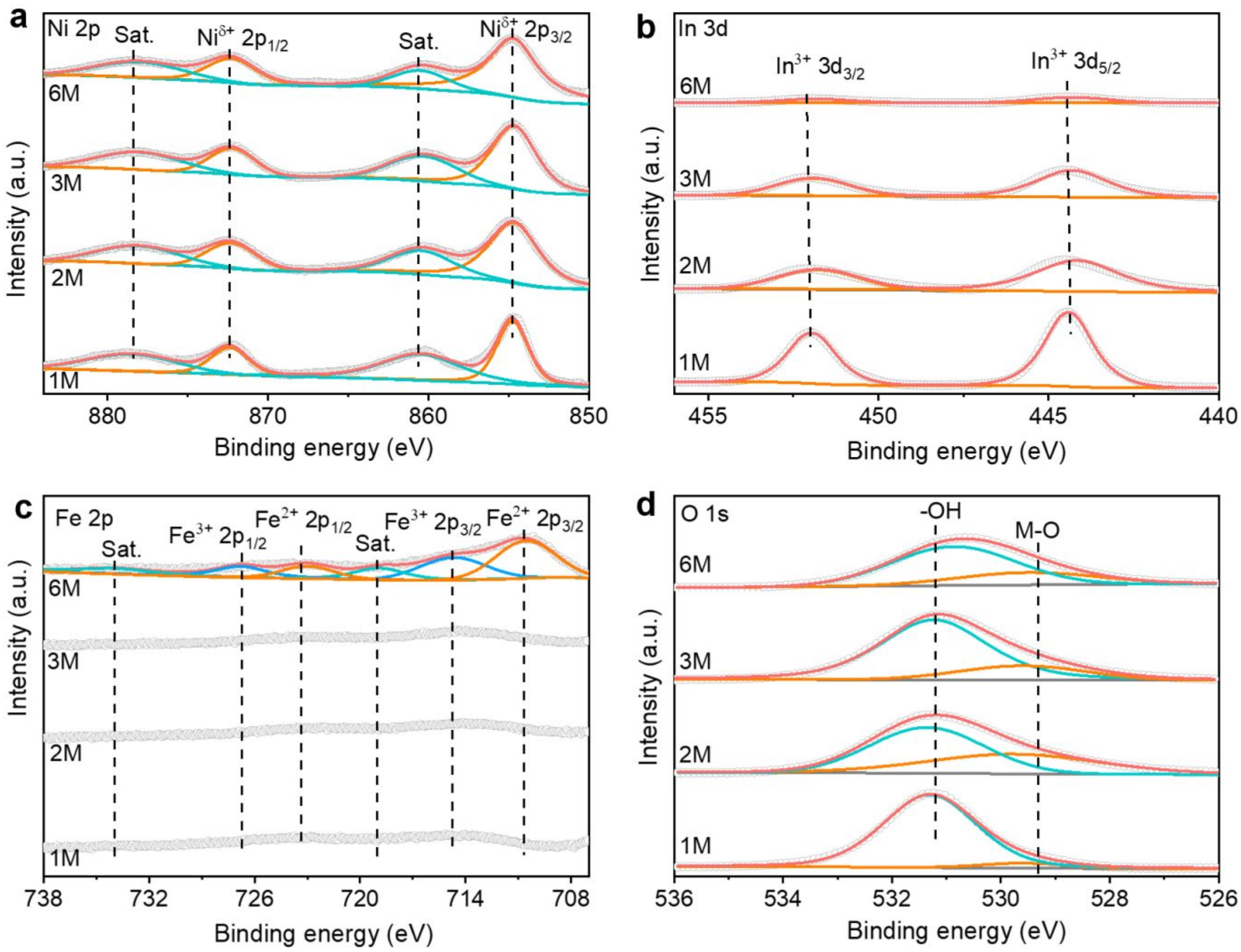

Figure S16. The XPS spectra of (a) Ni 2p, (b) In 3d, (c) Fe 2p and (d) O 1s for Ni_2In_3/FTO-1M, Ni_2In_3/FTO-2M, Ni_2In_3/FTO-3M and Ni_2In_3/FTO-6M, respectively. In the four reconstructed samples, the Ni XPS spectra were nearly identical, indicating a $Ni^{\delta+}$ oxidation state. The peaks at about 860.5 eV represent the satellite peaks.[4, 15] Indium, existing in the +3 state,[16, 19] showed a clear decrease in the peak intensity with increasing KOH concentration, further supporting the occurrence of indium leaching. Although an additional 100 scans were collected for the Fe 2p region compared to 25 scans for other elements among all samples, only Ni_2In_3/FTO-6M showed a detectable Fe signal, corresponding to Fe^{2+} and Fe^{3+}.[20, 21] The O 1s spectra also revealed an increasing proportion of M–O bonds with higher KOH concentration.[22]

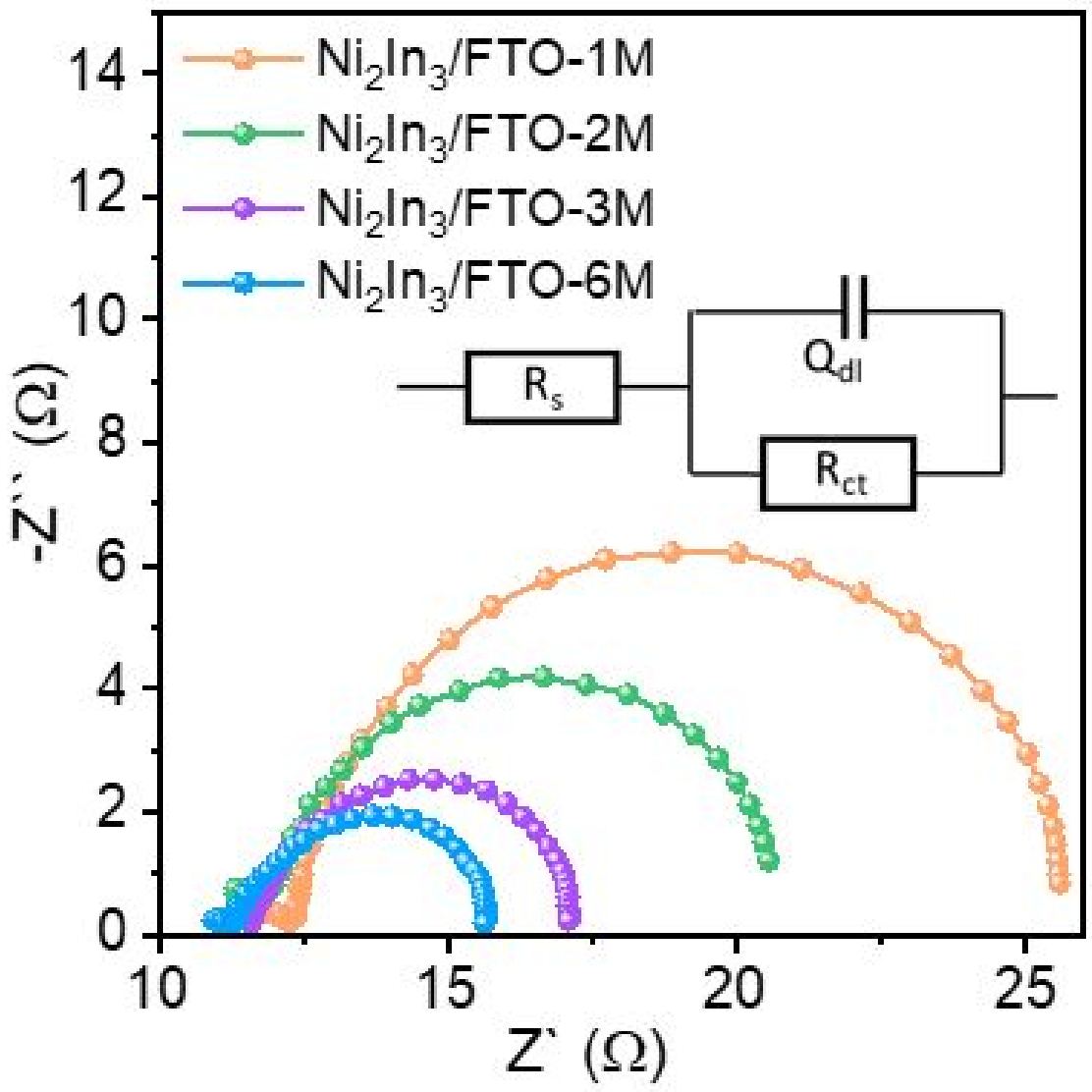

Figure S17. EIS measurements conducted at 1.56 V_{RHE} in 1M KOH after activation in different concentrations of KOH. The curves were fitted based on an equivalent circuit model shown in the figure, and the fitting parameters are shown in Table S1. The equivalent circuit consists of a solution resistance (R_s) in series with a parallel combination of the charge-transfer resistance (R_{ct}) and the double-layer capacitance, the latter modelled as a constant phase element (Q_{dl}, a). For these catalysts at 1.56 V_{RHE}, the solution resistance and constant phase element are likely to also model the redox transitions of the catalysts (rather than the pure solution resistance and double-layer surface capacitance).

Table S1. The fitting parameters of the impedance spectra as shown in Figure S17 for Ni_2In_3/FTO-*n*M.

Samples	R_s (Ω)	R_{ct} (Ω)	Q ($F \cdot s^{(a-1)}$)	a
Ni_2In_3/FTO-1M	12.3	14.3	0.017	0.906
Ni_2In_3/FTO-2M	11.8	9.6	0.016	0.892
Ni_2In_3/FTO-3M	11.6	6.1	0.022	0.859
Ni_2In_3/FTO-6M	11.2	4.6	0.029	0.873

a: dimensional parameter between 0 and 1.

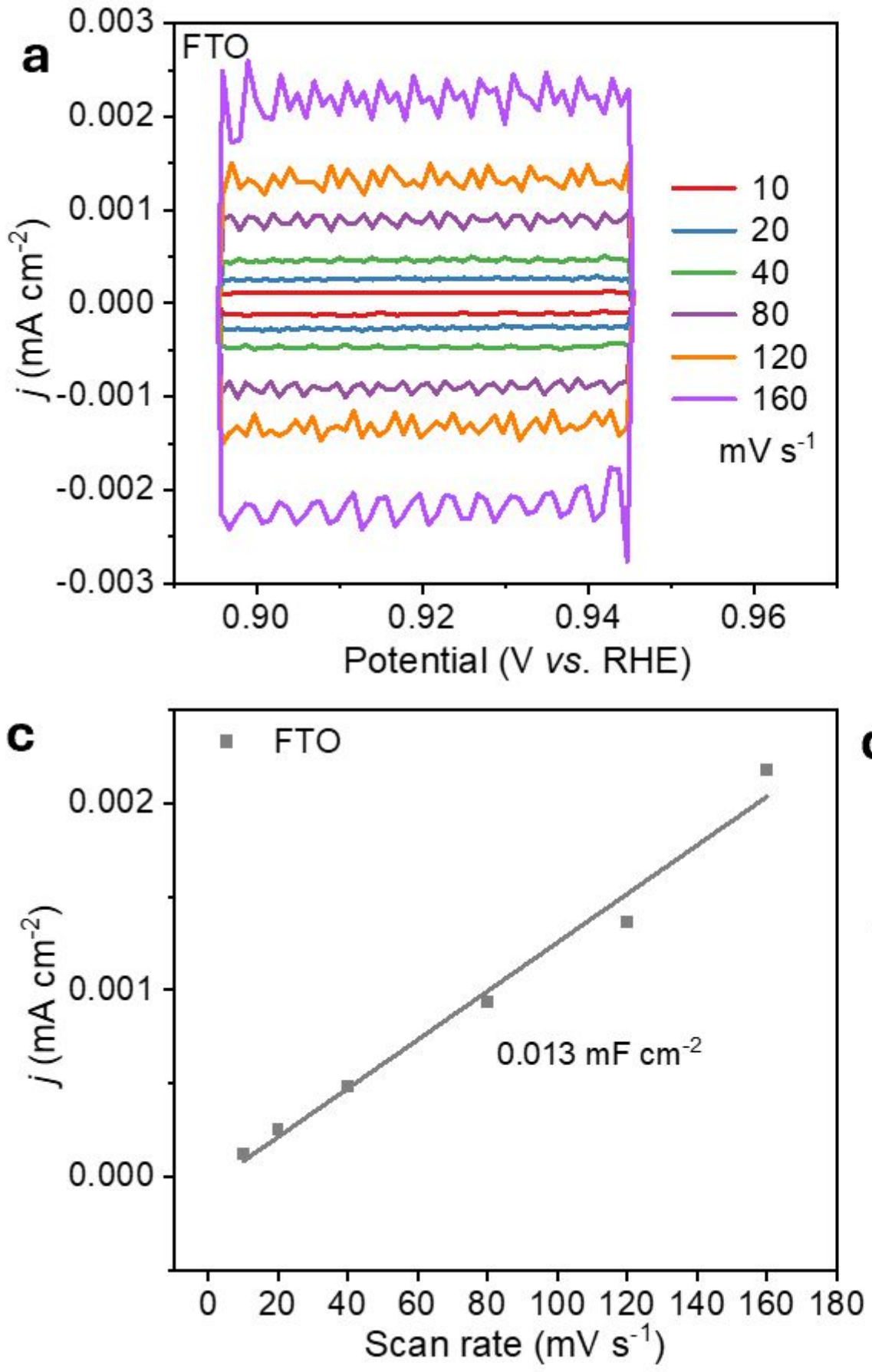

Figure S18. CVs with different scan rates for bare FTO (a) and Ni_2In_3/FTO-6M-Fe (b). (e) The plot of (j_{anodic}-$j_{cathodic}$)/2 at 0.92 V_{RHE} vs. scan rate for FTO (c) and Ni_2In_3/FTO-6M-Fe (d). The capacitive current densities of Ni_2In_3/FTO-6M-Fe are very low and comparable to those of bare FTO. The fitted slopes are also nearly identical, giving C_{dl} values of 0.012 and 0.013 mF cm^{-2} for Ni_2In_3/FTO-6M-Fe and FTO, respectively. This indicates that the measured response is largely dominated by the FTO substrate, making the C_{dl}-derived ECSA unreliable and not meaningful for evaluating the intrinsic surface area of the catalyst.

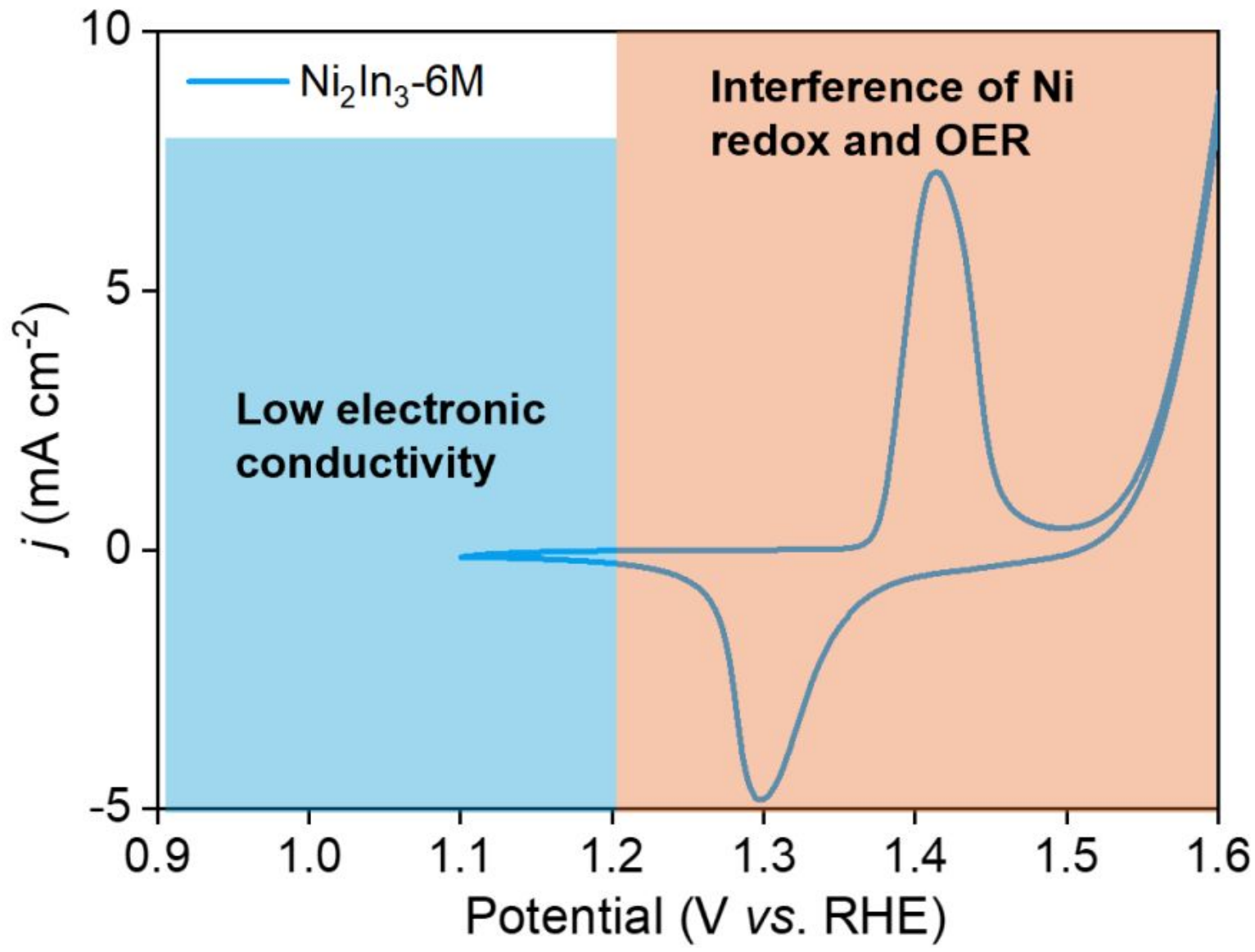

Figure S19. Challenges measuring meaningful double layer capacitances (C_{dl}) or electrochemical surface areas (ECSA) for Ni-based catalysts. A valid C_{dl} measurement requires a potential window free of Faradaic processes (both Ni redox and OER). For Ni-based materials, such a window would be below ~1.1 V vs. RHE, where Ni exists primarily as $Ni(OH)_2$. However, $Ni(OH)_2$ is known to possess poor electronic conductivity, which can significantly affect the measured capacitive current and compromise the reliability of the derived ECSA values. In a nutshell, the interference from Ni redox and OER at high potentials, combined with the poor electronic conductivity at low potentials, makes accurate C_{dl} measurements of Ni-based electrocatalysts very difficult.

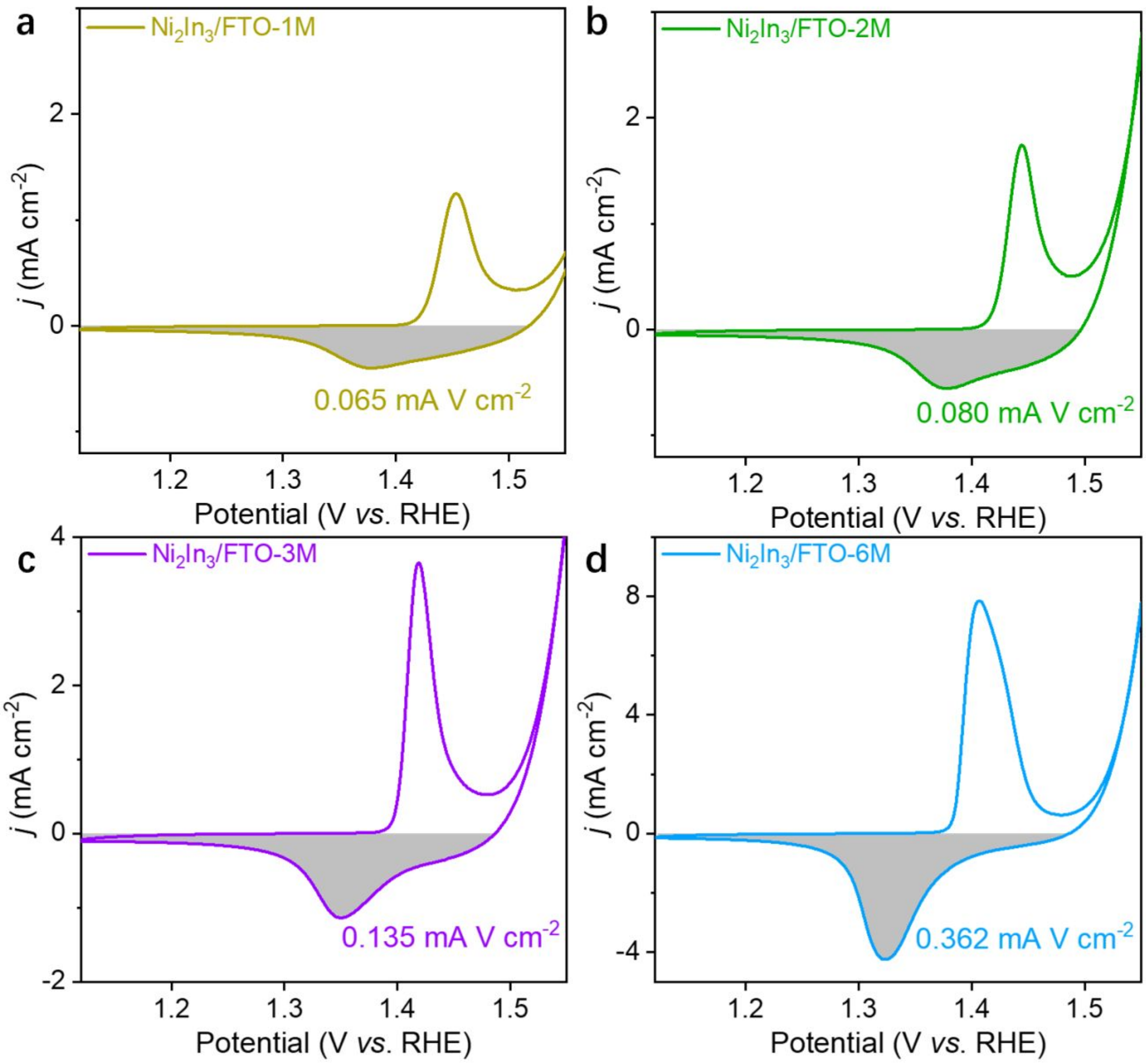

Figure S20. Integration of the reduction peaks in the CV curves of (a) Ni_2In_3/FTO-1M, (b) Ni_2In_3/FTO-2M, (c) Ni_2In_3/FTO-3M, and (d) Ni_2In_3/FTO-6M, measured in 1M KOH after activation. A pair of peaks between 1.2 V_{RHE} and 1.5 V_{RHE} corresponds to the Ni(II/III/IV) redox process in alkaline media. The integration approach involves integrating (summing) all negative current values from both the anodic and cathodic scans.

Table S2. Calculation of the number of moles of electrons transferred during the reaction based on the integrated area of the reduction peak for Ni_2In_3/FTO-*n*M. V_1 and V_2 were defined as the potentials at which the CV current first deviated from and subsequently returned to 0 mA cm^{-2}, respectively. The integrated area between the CV curve and the 0 mA cm^{-2} baseline within this potential range—including both anodic and cathodic currents— was calculated to obtain the charge associated with the Ni(II/III/IV) redox process.

Samples	$\int_{V_1}^{V_2} I(E)\,dE$ (mA · V)	$[e^-]$ (μmol)
Ni_2In_3/FTO-1M	0.065	0.134
Ni_2In_3/FTO-2M	0.080	0.165
Ni_2In_3/FTO-3M	0.135	0.279
Ni_2In_3/FTO-6M	0.362	0.748

$$[e^-] = \frac{1}{\text{scan rate} \times F} \times \int_{V_1}^{V_2} I(E)\,dE$$

Scan rate= 5 mV/s, F=96485 s·A/mol

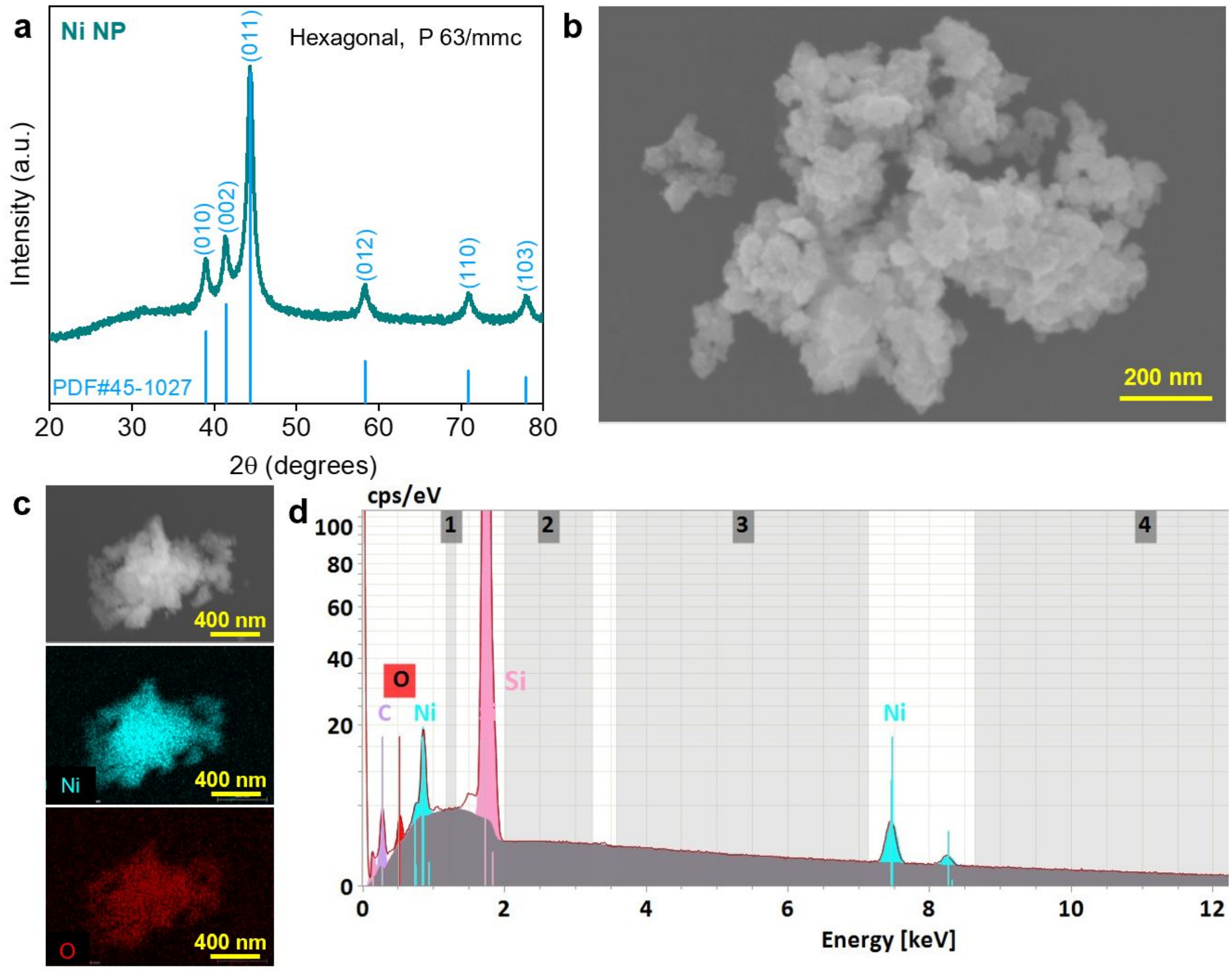

Figure S21. Structural characterization of Ni nanoparticles. (a) Powder XRD, (b) SEM image, (c) elemental mapping, and (d) EDX. XRD results confirm that the Ni nanoparticles are of a pure phase, crystallizing in the hexagonal system (PDF No. 45-1027). SEM indicates that the particles are small in size and uniformly distributed. Elemental mapping and EDX further verify the presence of Ni, with the Si signal originating from the silicon wafer. A small C signal is likely associated with surfactant residues or contamination by carbon-containing compounds, whereas the detected O signal can be attributed to partial surface oxidation of the nanoparticles.

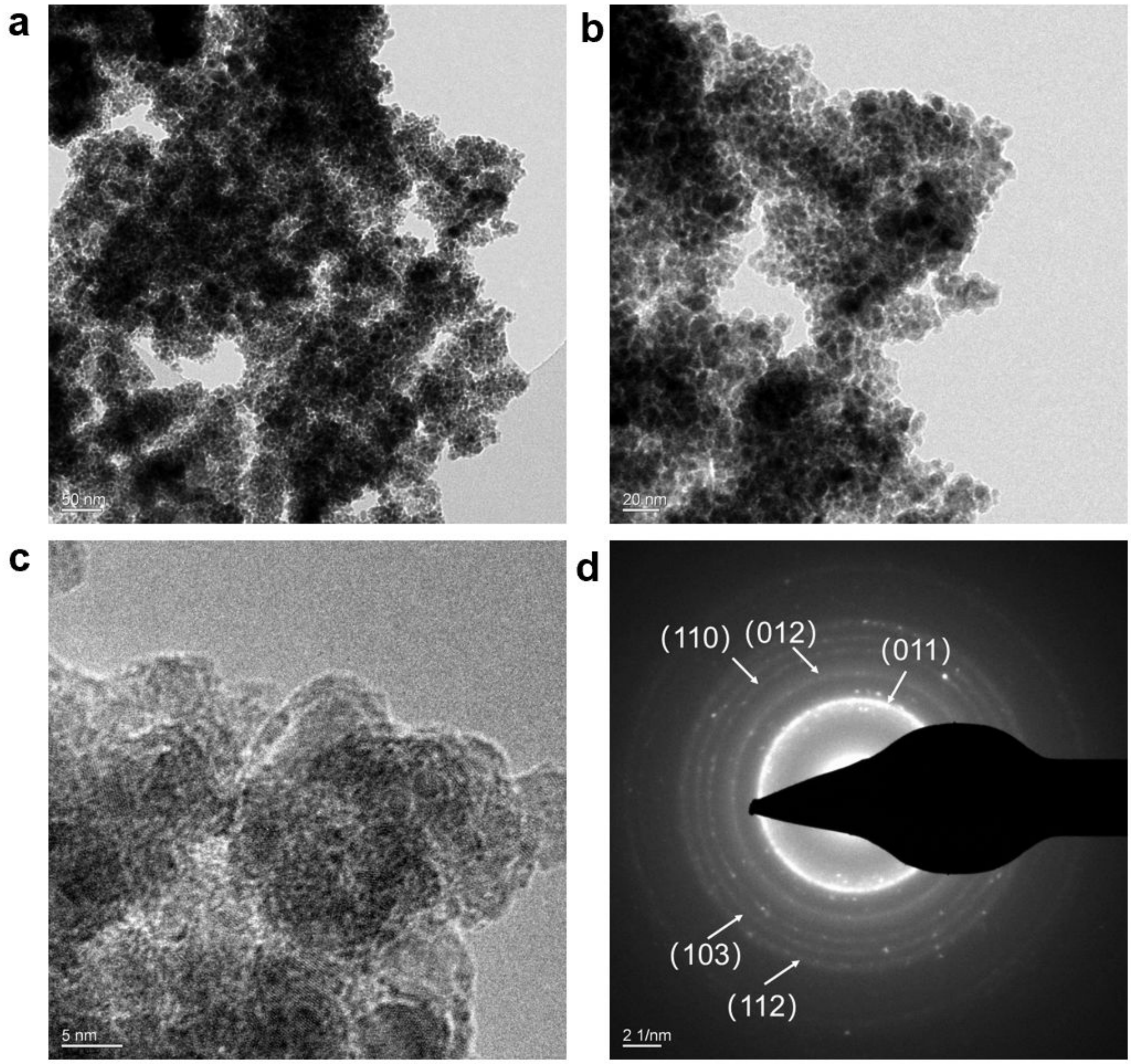

Figure S22. The (a-c) TEM images and (d) the corresponding SAED pattern of Ni nanoparticles. The SAED pattern confirms the presence of crystalline planes (011), (012), (110), (103), and (112) in the Ni phase, which perfectly match the XRD peaks.

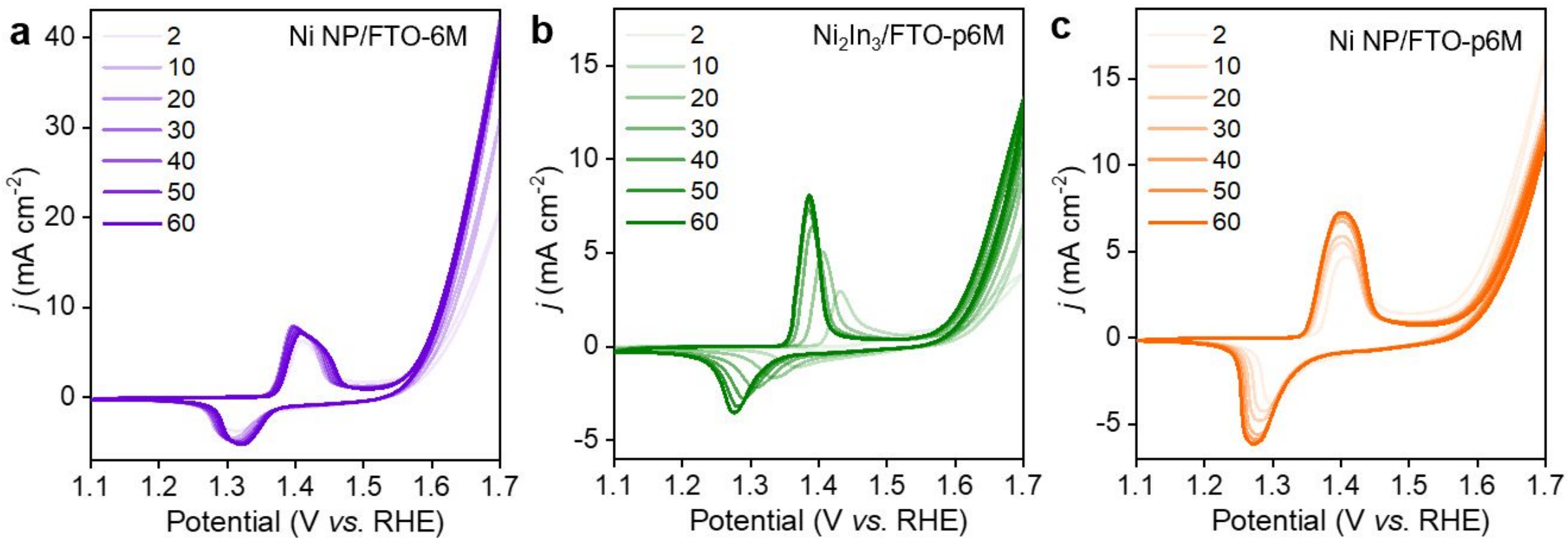

Figure S23. The CV curves of (a) Ni NP/FTO activated in 6M KOH, (b) Ni_2In_3/FTO, and (c) Ni NP/FTO activated in purified 6M KOH. The scan rate is 5 mV/s.

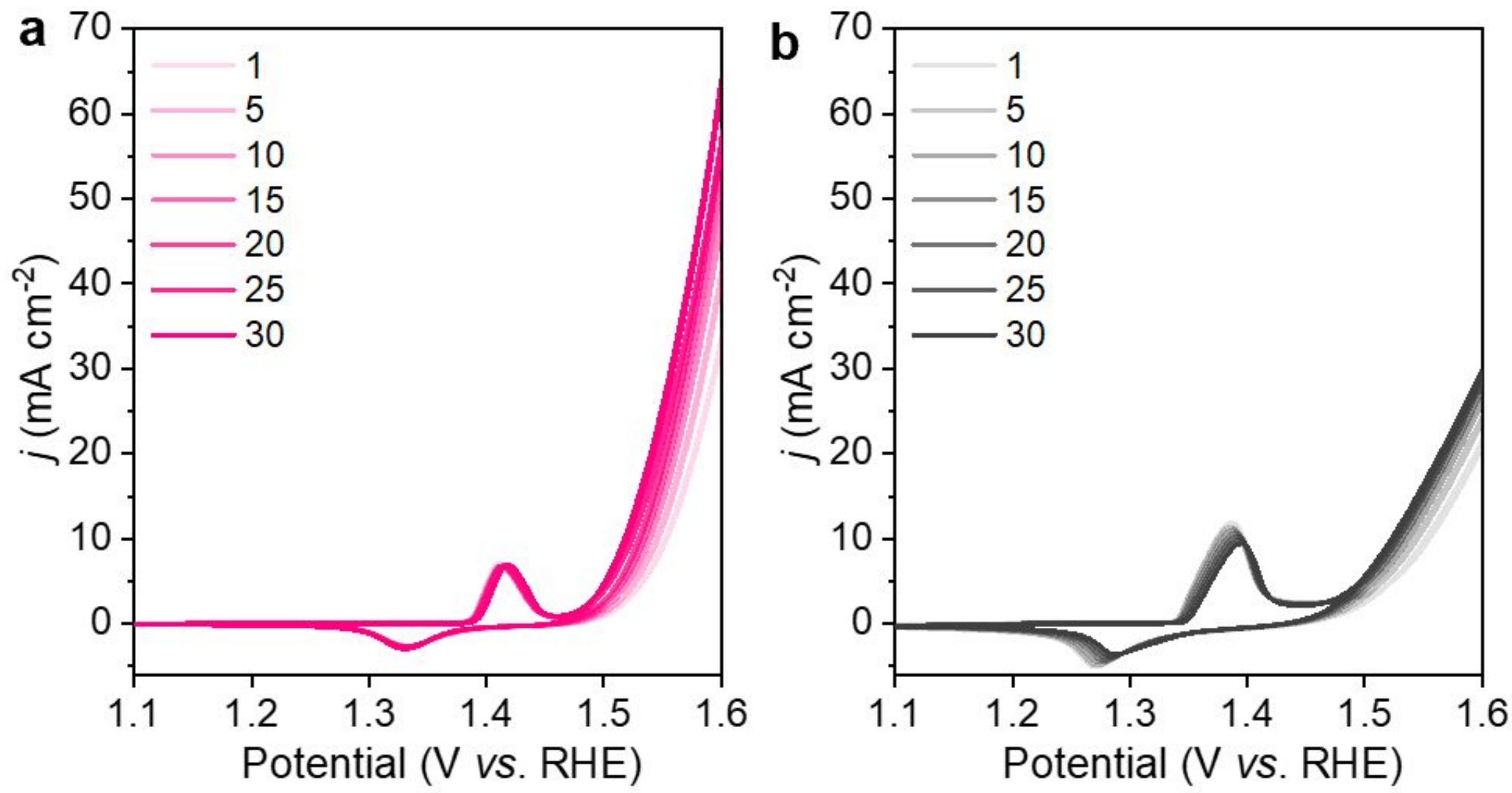

Figure S24. The CV curves during Fe spiking in the potential range of 1.1–1.6 V_{RHE} in 40 mL of 1M KOH with 5 mM $FeSO_4$ xH_2O for (a) Ni_2In_3/FTO-6M and (b) Ni NP/FTO-6M. The scan rate is 5 mV/s.

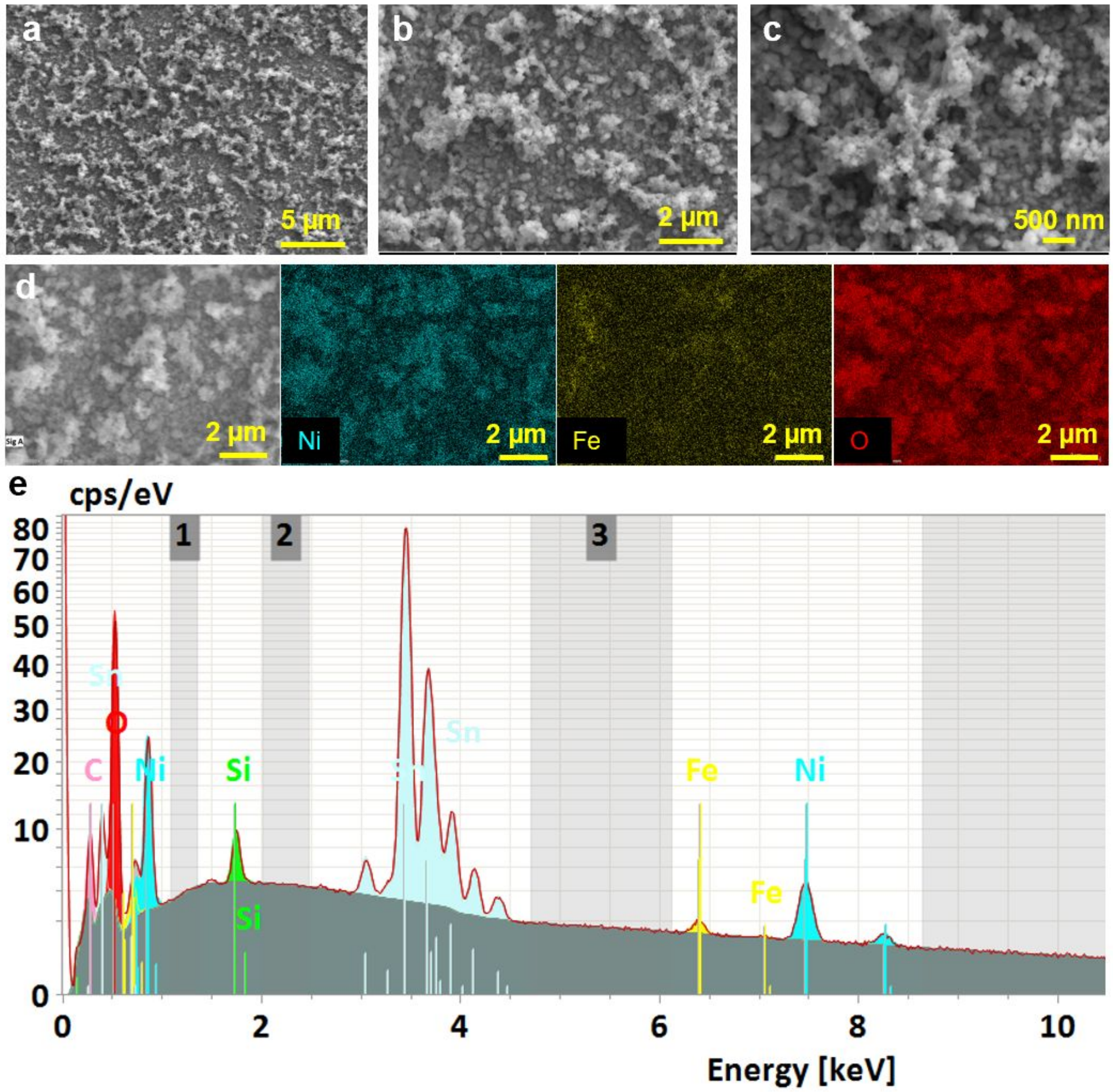

Figure S25. Structural characterization of $Fe_xNi_{1-x}OOH$/FTO. (a) SEM image, (c) elemental mapping and (d) EDX. These results indicate that $Fe_xNi_{1-x}OOH$/FTO consists of irregularly shaped particles with relatively large sizes. Elemental mapping and EDX further verify the presence of Ni, Fe, and O, while Sn originates from the FTO substrate and the Si signal from the silicon wafer. A weak C signal is likely associated with surfactant residues or contamination by carbon-containing compounds.

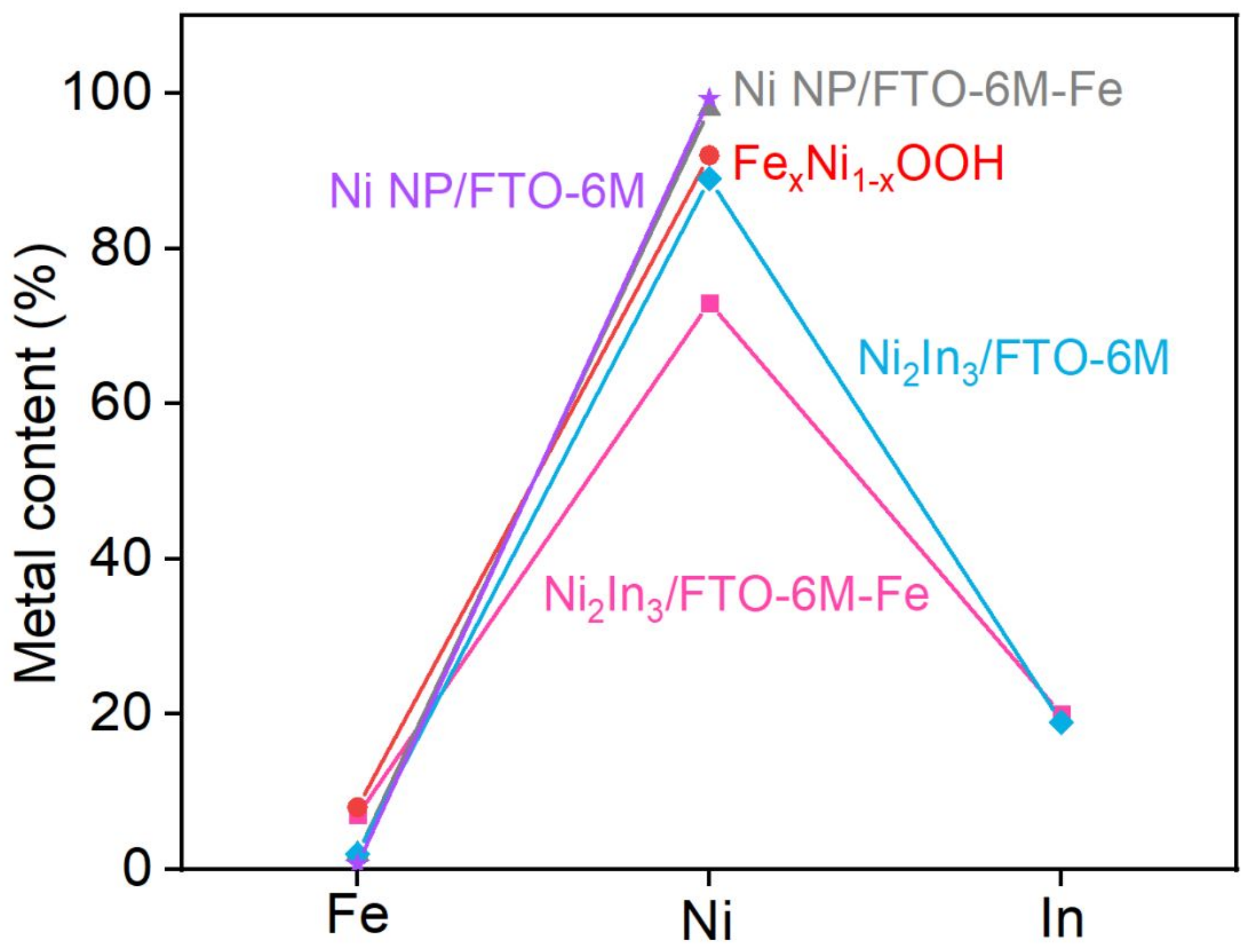

Figure S26. The ICP-OES results of the relative metal content (in percentage) of Fe, Ni, and In elements (the total metal content normalized to 100%) in Ni_2In_3/FTO-6M, Ni_2In_3/FTO-6M-Fe, $Fe_xNi_{1-x}OOH$/FTO, Ni NP/FTO-6M, and Ni NP/FTO-6M-Fe. The resulting compositions were approximately determined as Ni_2In_3/FTO-6M ($Fe_{0.02}Ni_{0.79}In_{0.19}OOH$), Ni_2In_3/FTO-6M-Fe ($Fe_{0.07}Ni_{0.73}In_{0.20}OOH$), $Fe_xNi_{1-x}OOH$/FTO ($Fe_{0.08}Ni_{0.92}OOH$), Ni NP/FTO-6M ($Fe_{0.01}Ni_{0.99}OOH$) and Ni NP/FTO-6M-Fe ($Fe_{0.02}Ni_{0.98}OOH$).

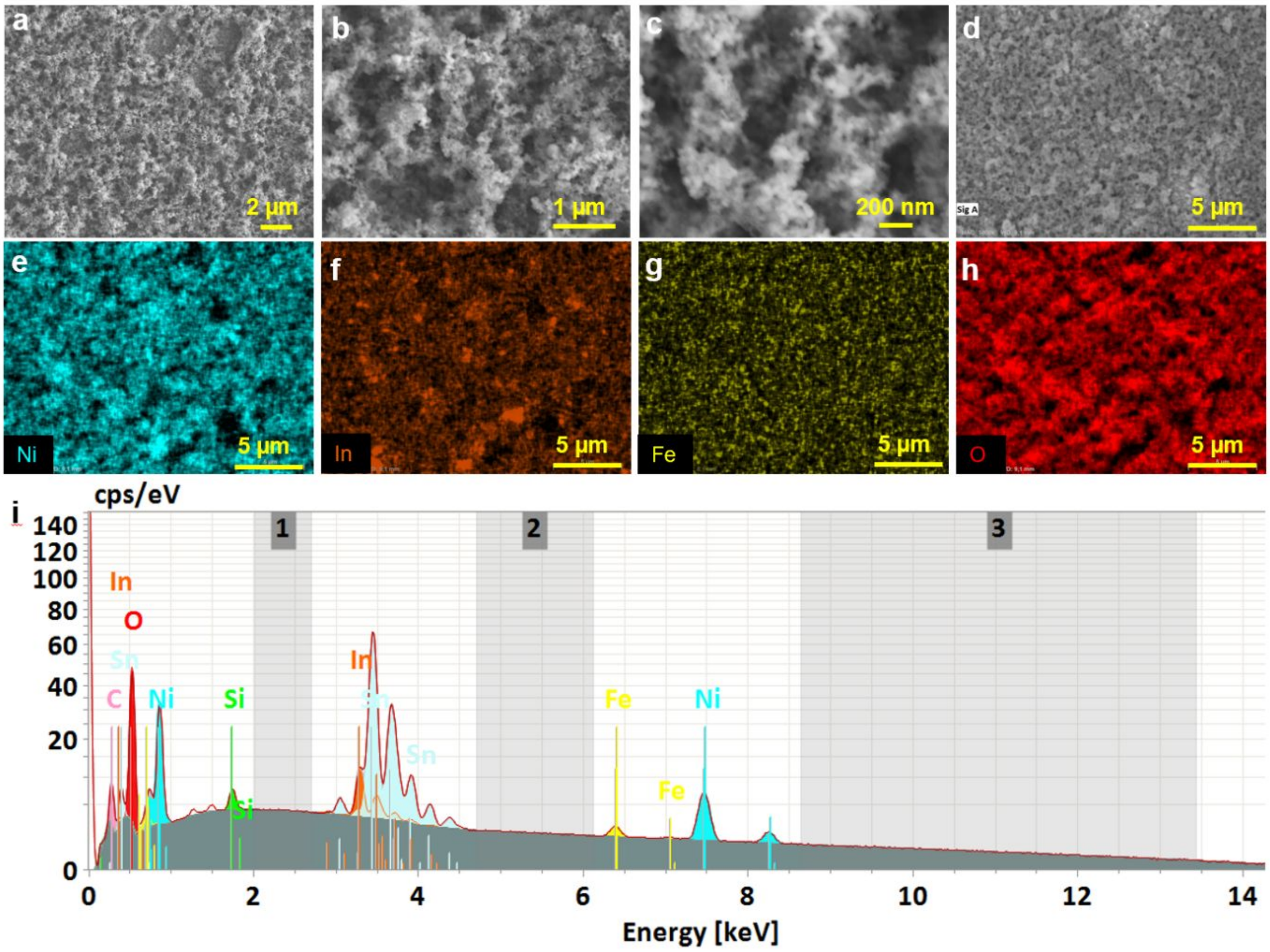

Figure S27. (a-c) SEM images, (d-h) elemental mapping, and (i) EDX of Ni_2In_3/FTO-6M-Fe. Elemental mapping and EDX further verify the presence of Ni, In, Fe, and O, while Sn originates from the FTO substrate and the Si signal from the silicon wafer. A weak C signal is likely associated with surfactant residues or contamination by carbon-containing compounds.

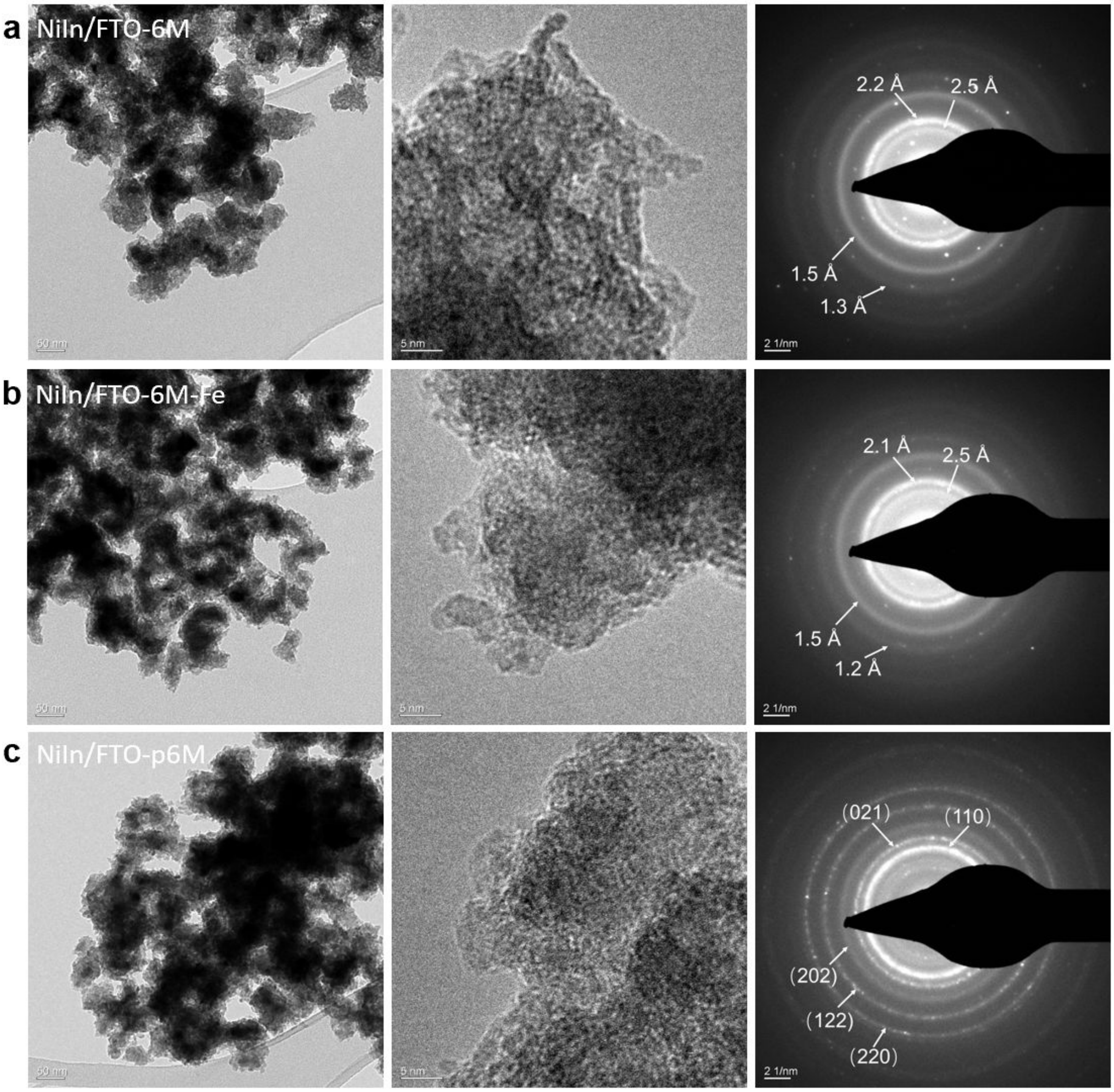

Figure S28. The TEM images and the corresponding SAED pattern of (a) Ni_2In_3/FTO-6M, (b) Ni_2In_3/FTO-6M-Fe, and (c) Ni_2In_3/FTO-p6M, respectively. In (a-b), the SAED patterns indicate that the primary phase is Ni (oxy)hydroxides. In (c), the SAED pattern of Ni_2In_3/FTO-p6M confirms the presence of the (110), (021), (202), (122), and (220) crystalline planes of Ni_2In_3 (PDF No. 65–7718).

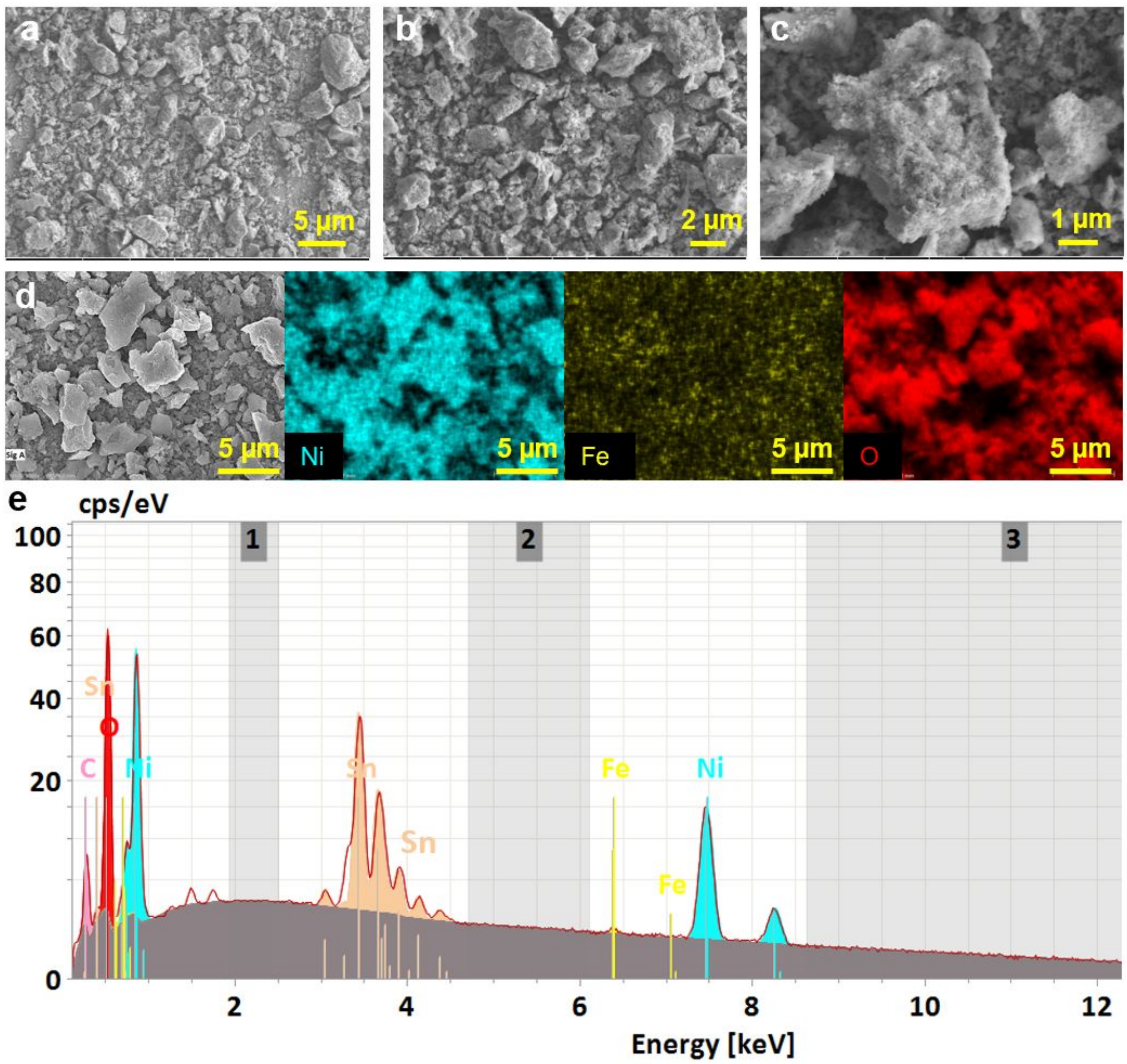

Figure S29. (a-c) SEM images, (d) elemental mapping, and (e) EDX of Ni NP/FTO-6M. Ni particles exhibit pronounced aggregation and growth. Elemental mapping and EDX verify the presence of Ni, Fe, and O, while Sn originates from the FTO substrate. A small C signal is likely associated with surfactant residues or contamination by carbon-containing compounds.

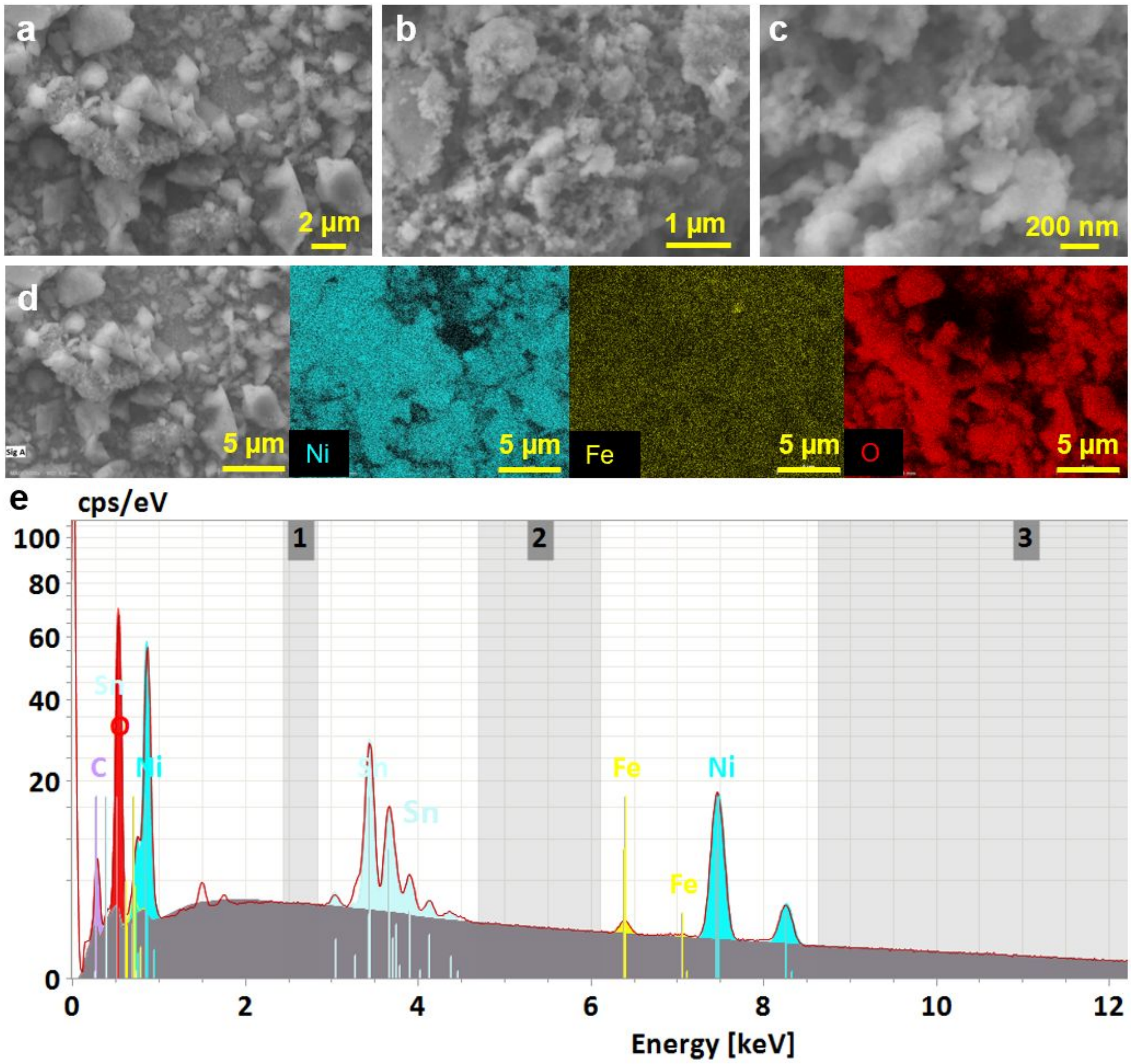

Figure S30. (a-c) SEM images, (d) elemental mapping, and (e) EDX of Ni NP/FTO-6M-Fe. Elemental mapping and EDX verify the presence of Ni, Fe, and O, while Sn originates from the FTO substrate. A small C signal is likely associated with surfactant residues or contamination by carbon-containing compounds.

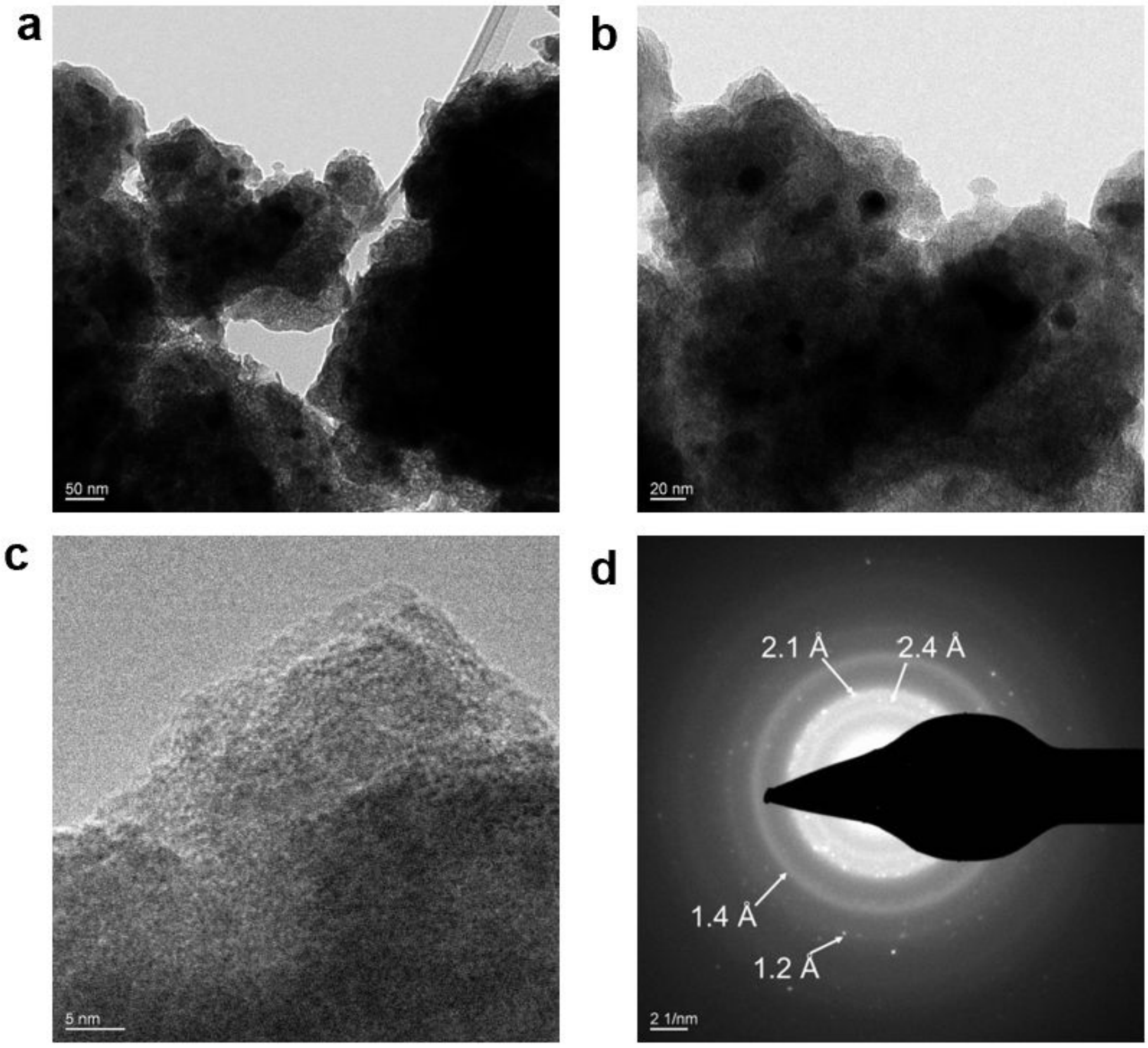

Figure S31. The (a-c) TEM images and (d) the corresponding SAED pattern of Ni NP/FTO-6M-Fe. The SAED patterns indicate that the primary phase is either NiOOH or $Ni(OH)_2$, which are difficult to distinguish due to their similar diffraction features. Therefore, the phase is most likely a mixture of the two, appropriately denoted as Ni (oxy)hydroxides.

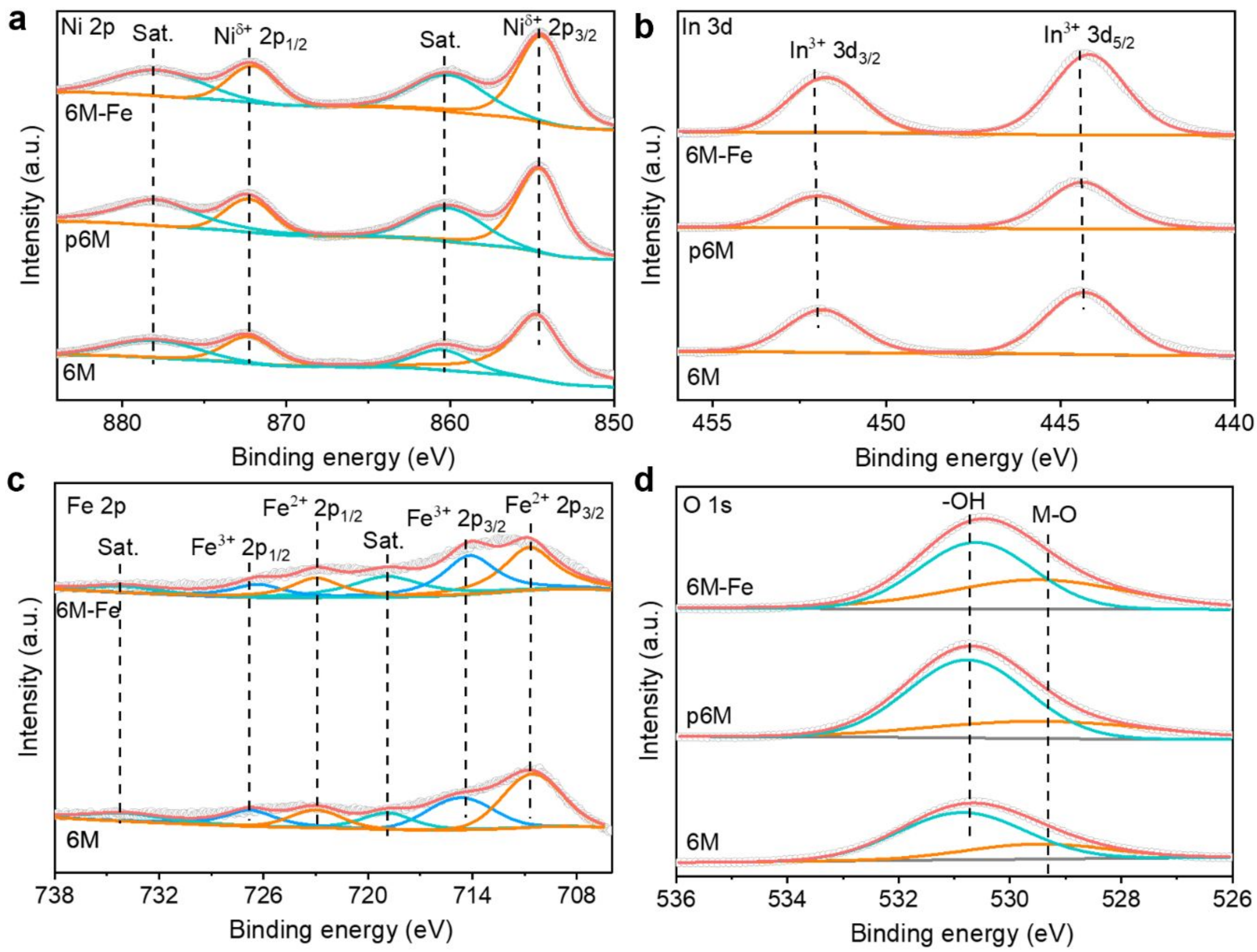

Figure S32. The XPS spectra of (a) Ni 2p, (b) In 3d, (c) Fe 2p and (d) O 1s for Ni_2In_3/FTO-6M, Ni_2In_3/FTO-p6M and Ni_2In_3/FTO-6M-Fe, respectively. In the three samples, the Ni XPS spectra were nearly identical. The peaks located at about 854.7 and 872.2 eV are assigned to $Ni^{\delta+}$, whereas those at about 860.5 and 878.1 eV are attributed to satellite peaks.[4, 15] Indium exists in the +3 oxidation state, with no significant differences observed among the three samples.[16, 19] The Ni_2In_3/FTO-6M data shown here are the same as those in Figure S16. The In^{3+} peak in (b) appears clearer because the Y-axis intensity scale is approximately 8 times that of Figure S16. Although an additional 100 scans were collected for the Fe 2p region compared to 25 scans for other elements among all samples, Fe signals are still very weak, corresponding to Fe^{2+} and Fe^{3+}.[20, 21]

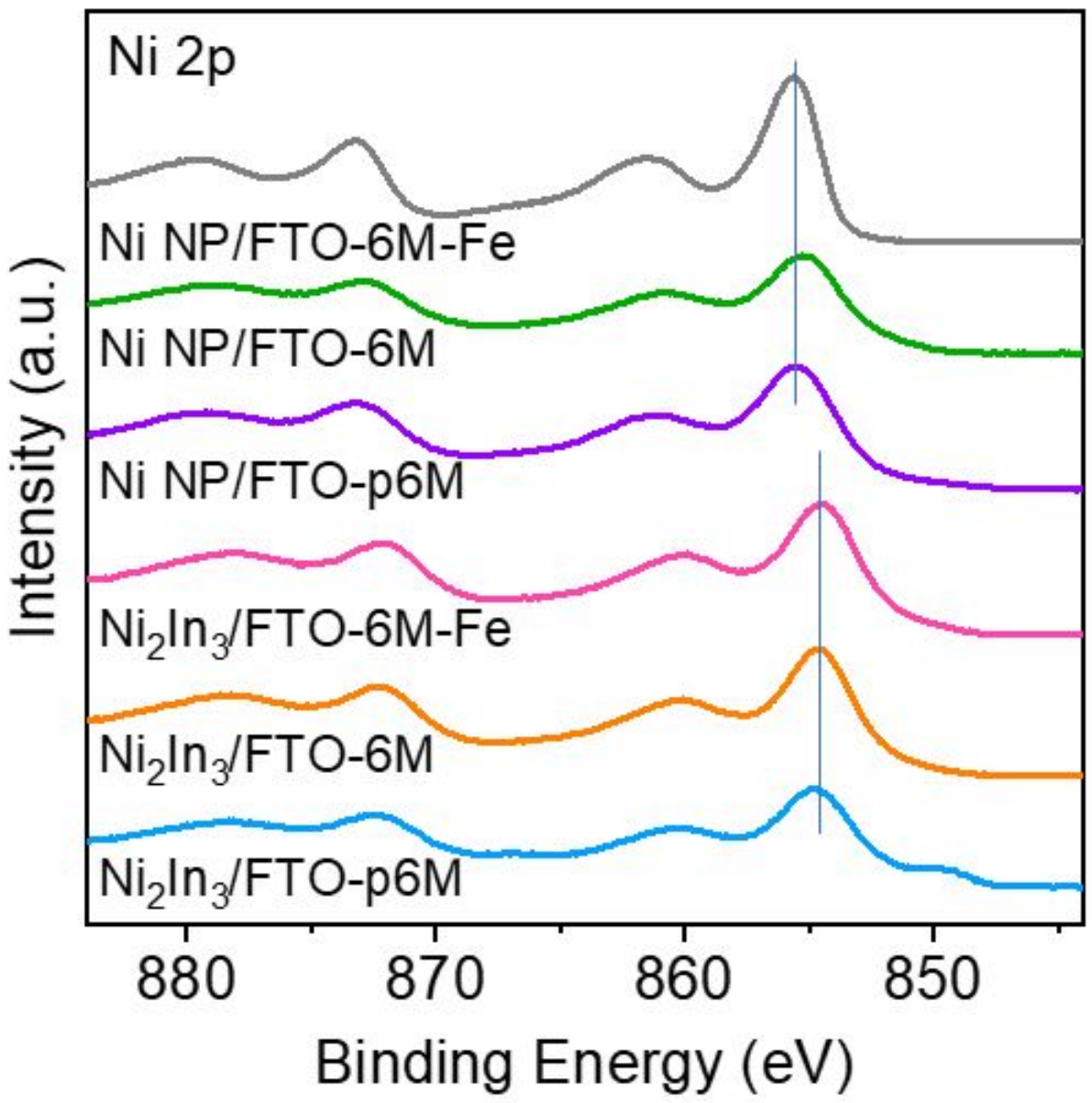

Figure S33. The Ni 2p XPS spectra of Ni NP-derived and the Ni_2In_3-derived catalysts. This indicates that Ni existed in a δ^+ oxidation state,[23] which is slightly higher in the Ni NP-derived catalysts than in the Ni_2In_3-derived ones.

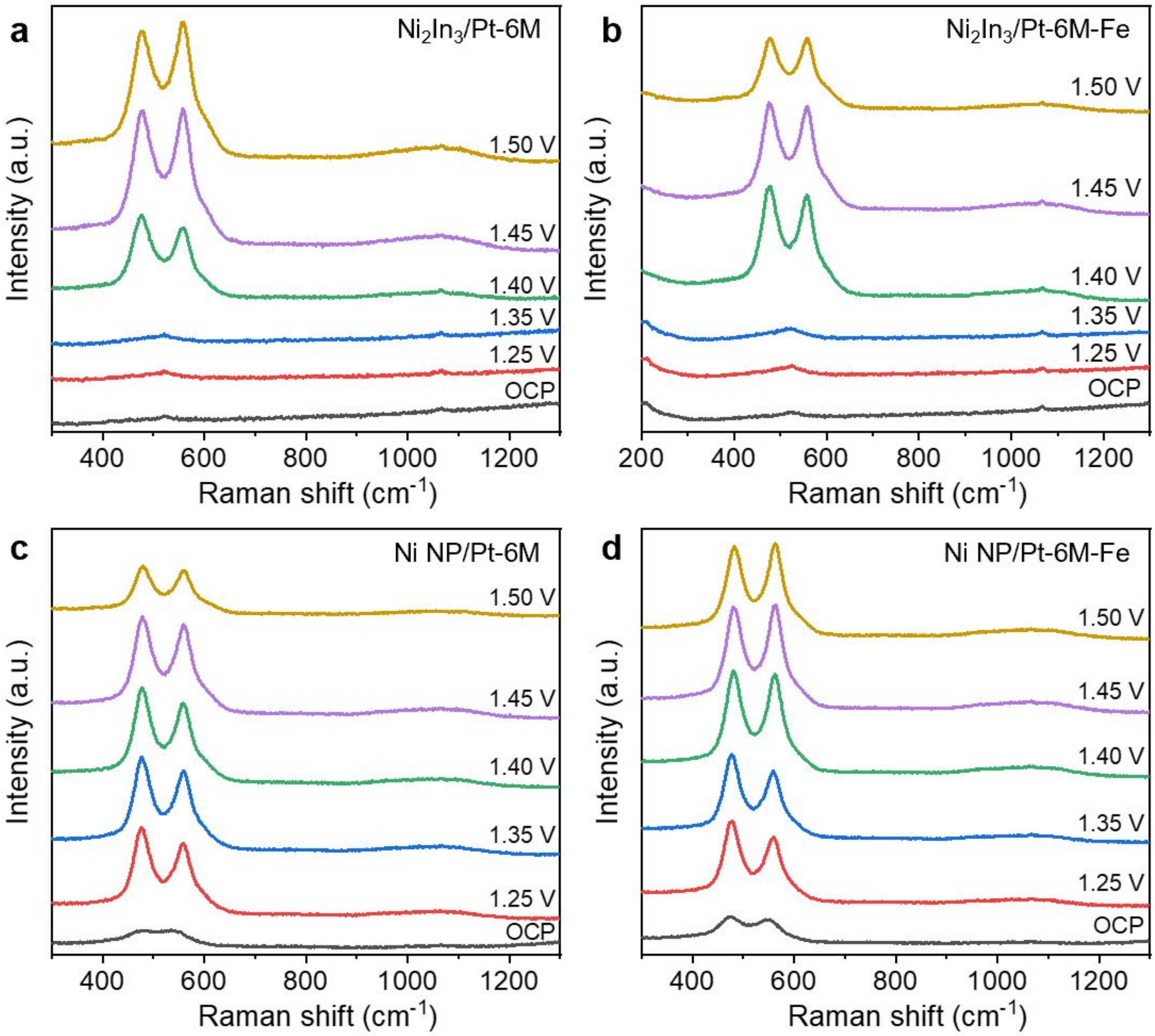

Figure S34. The *in situ* Raman spectra of (a) Ni_2In_3/Pt-6M, (b) Ni_2In_3/Pt-6M-Fe, (c) Ni NP/Pt-6M, and (d) Ni NP/Pt-6M-Fe. Raman Spectra were collected in chronoamperometry (CA) mode at OCP, 1.25 V, 1.35 V, 1.40 V, 1.45 V, and 1.50 V vs. RHE. Before each measurement, the samples had been activated under their respective conditions (See section 1.6), and the potential was held for 5 min at each step prior to spectral acquisition. All tested samples exhibited characteristic peaks of NiOOH in the OER potential region, located at approximately 478 and 560 cm^{-1}, corresponding to δ(Ni^{3+}–O) and ν(Ni^{3+}–O), respectively.[4, 5] However, for Ni_2In_3-derived catalysts, these characteristic peaks only began to appear when the potential reached 1.40 V_{RHE}. In contrast, Ni NP-derived catalysts already displayed NiOOH features under OCP conditions, with these peaks becoming prominent starting from 1.25 V_{RHE}, indicating that the oxidation of Ni occurs more easily.

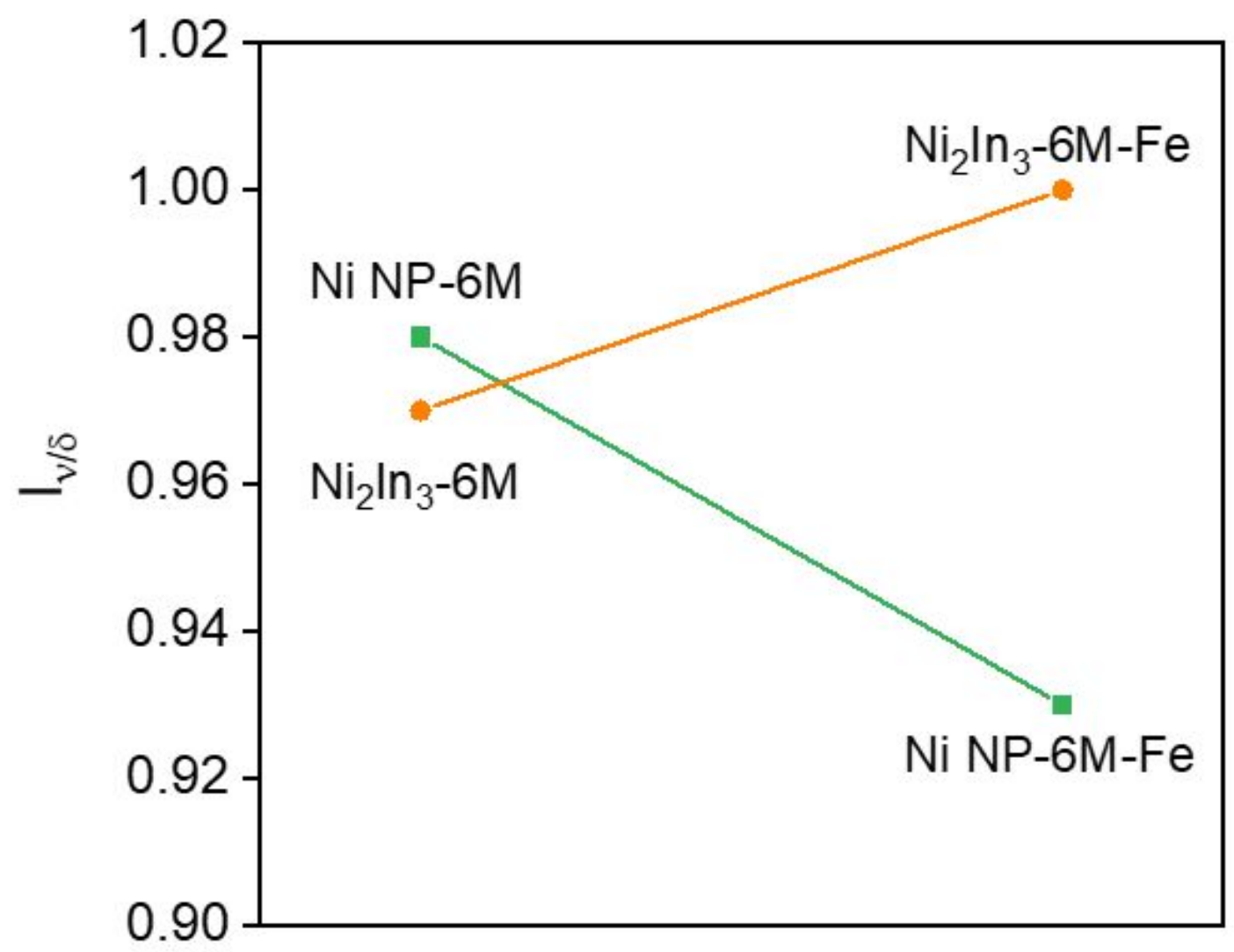

Figure S35. The two Raman characteristic peaks intensity ratio of ν(Ni^{3+}–O) to δ(Ni^{3+}–O) for Ni_2In_3/Pt-6M, Ni_2In_3/Pt-6M-Fe, Ni NP/Pt-6M, and Ni NP/Pt-6M-Fe.

A closer examination of the *in situ* Raman spectra revealed a slight variation in the relative intensities of the two characteristic peaks. The intensity ratio of ν(Ni^{3+}–O) to δ(Ni^{3+}–O), denoted as $I_{\nu/\delta}$, is 0.98 for Ni NP-6M-Fe, higher than the 0.93 observed for Ni NP-6M (**Figure 4c**), which aligns with previous reports that Fe incorporation modulates the local environment of Ni–O.[24] However, when comparing Ni_2In_3-6M-Fe with Ni_2In_3-6M, Fe incorporation does not lead to a noticeable increase in $I_{\nu/\delta}$, suggesting that perhaps Fe is primarily incorporated at the interface and has a limited effect on the local environment of Ni–O.

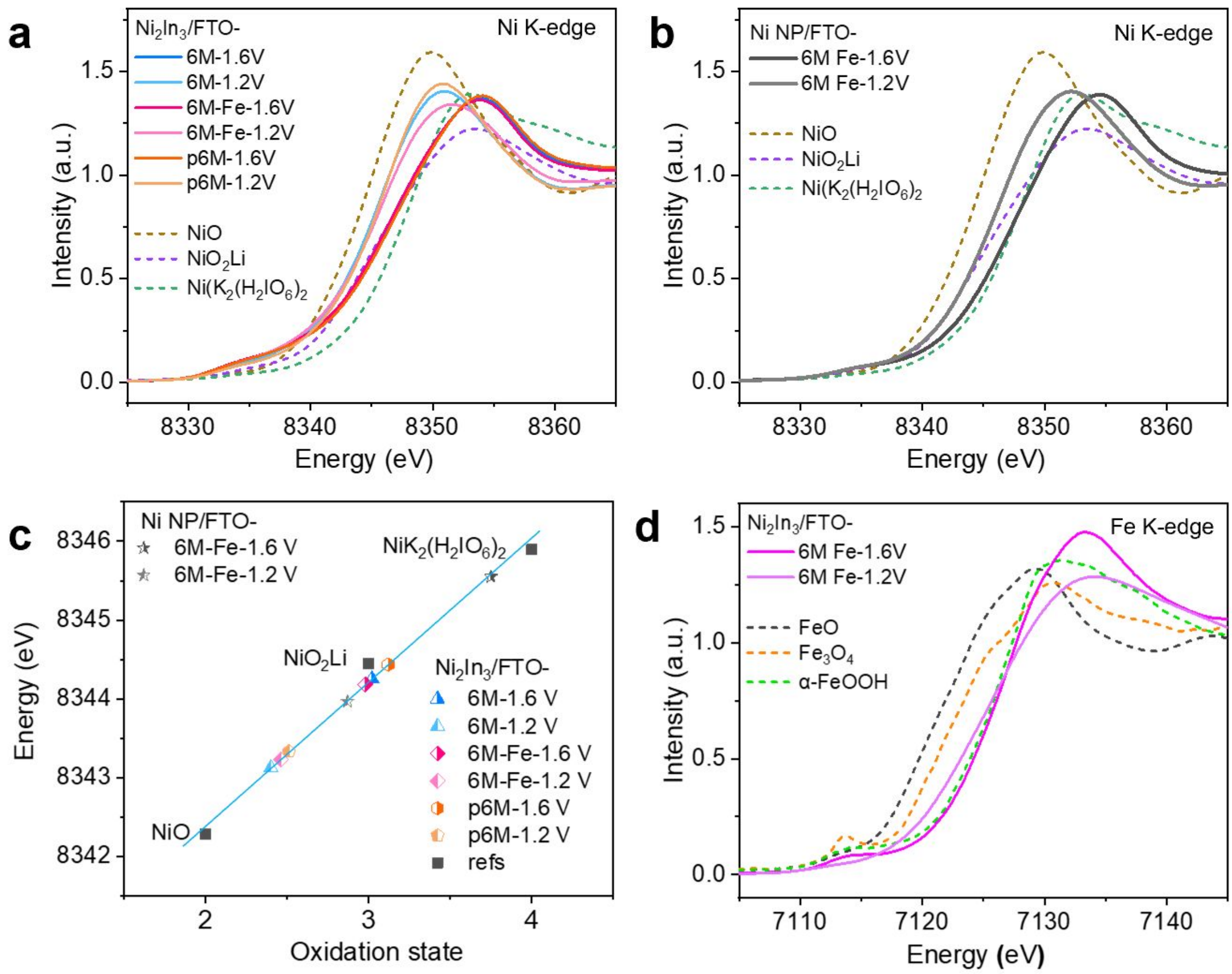

Figure S36. (a) Quasi-in-situ XANES spectra at the Ni K-edge for Ni_2In_3-derived catalysts and Ni reference compounds. (b) Quasi-in-situ XANES spectra at the Ni K-edge for Ni NP/FTO-6M-Fe and Ni reference compounds. (c) The obtained values of Ni edge positions were plotted and converted to oxidation states in a linear regression (blue line) based on the reference compounds. (d) Quasi-in-situ XANES spectra at the Fe K-edge for Ni_2In_3/FTO-6M-Fe and Fe reference compounds.

Table S3. Bulk oxidation states of Ni in the samples obtained from quasi-in-situ XANES. $\Delta_{1.6\text{-}1.2VRHE}$ represents the difference in oxidation state between 1.6 V_{RHE} and 1.2 V_{RHE}, which should be roughly proportional to the redox activity.

	Ni K-edge XANES oxidation states		
	**1.2 V_{RHE}**	**1.6 V_{RHE}**	**$\Delta_{1.6\text{-}1.2VRHE}$**
Ni_2In_3/FTO-p6M	2.51	3.12	0.61
Ni_2In_3/FTO-6M	2.40	3.02	0.62
Ni_2In_3/FTO-6M-Fe	2.46	2.98	0.52
Ni NP/FTO-6M-Fe	2.87	3.75	0.88

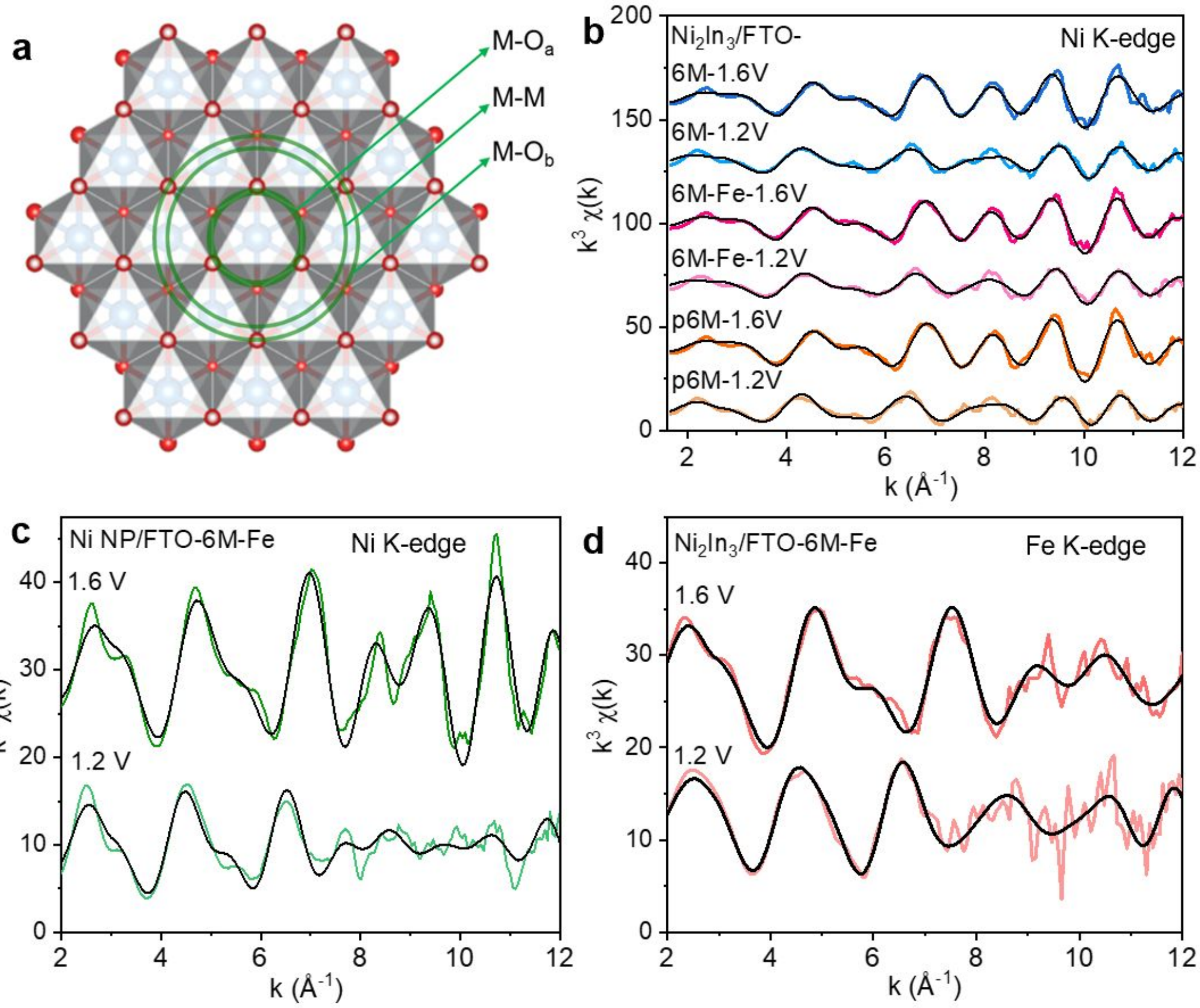

Figure S37. (a) The layered model structure for metal (oxy)hydroxides comprising edge-sharing octahedra. Quasi-in-situ k^3-weighted EXAFS spectra at the Ni K-edges were collected for Ni_2In_3-derived catalysts (b) and Ni NP/FTO-6M-Fe (c) freeze-quenched at 1.2 and 1.6 V_{RHE}. (d) Quasi-in-situ k^3-weighted EXAFS spectra at the Fe K-edges were collected for Ni_2In_3/FTO-6M-Fe freeze-quenched at 1.2 and 1.6 V_{RHE}. The fitted spectra are shown as black lines, and the corresponding fitting parameters are summarized in Tables S4–S7. The Fourier-transformed data are presented in Figure 4e–f of the main text.

Table S4. Parameters used for the Ni K-edge EXAFS simulations of samples freeze-quenched at 1.2 V_{RHE} based on the model of the layered structure for metal (oxy)hydroxides comprising edge-sharing octahedra. The model incorporates a single Ni–In shell for Ni_2In_3-derived catalysts or an additional Ni-Ni shell for Ni NP/FTO-6M-Fe. The fit-range was 1-4 Å. The simulated curves are shown as black lines in Figure 4e and Figure S37b-c. R_f represents the fitting error in %, N is the coordination number (or called population) of the shell at distance R, and σ is the Debye–Waller factor. The Debye–Waller factors were fixed at 0.05 and kept constant across all fits of the nickel–iron (oxy)hydroxides to reduce the number of free parameters. The amplitude reduction factor S_0^2 was set to 0.85. The coordination numbers for the first, third, and fifth shells were constrained to be equal, representing a phase with a high nickel oxidation state and shorter bond distances. Likewise, the coordination numbers for the second, fourth, and sixth shells were constrained to be equal, corresponding to a phase with a lower nickel oxidation state and longer bond distances.

Ni K-edge at 1.2 V_{RHE}	Shell	N	Error	R [Å]	Error [Å]	Shell	N	Error	R [Å]	Error [Å]
Ni_2In_3/FTO-p6M	Ni-O	1.8	0.2	1.94	0.02	Ni-O	2.8	0.4	2.08	0.02
Ni_2In_3/FTO-6M	Ni-O	1.9	0.3	1.89	0.03	Ni-O	2.7	0.3	2.06	0.03
Ni_2In_3/FTO-6M-Fe	Ni-O	2.2	0.3	1.86	0.03	Ni-O	2.1	0.3	2.03	0.04
Ni NP/FTO-6M-Fe	Ni-O	2.5	0.1	1.92	0.01	Ni-O	2.3	0.2	2.09	0.02
Ni_2In_3/FTO-p6M	Ni-Ni	1.8		2.92	0.04	Ni-Ni	2.8		3.08	0.02
Ni_2In_3/FTO-6M	Ni-Ni	1.9		2.90	0.03	Ni-Ni	2.7		3.07	0.02
Ni_2In_3/FTO-6M-Fe	Ni-Ni	2.2		2.89	0.03	Ni-Ni	2.1		3.06	0.02
Ni NP/FTO-6M-Fe	Ni-Ni	2.5		2.86	0.02	Ni-Ni	2.3		3.06	0.02
Ni_2In_3/FTO-p6M	Ni-O	1.8		3.48	0.09	Ni-O	2.8		3.70	0.05
Ni_2In_3/FTO-6M	Ni-O	1.9		3.48	0.06	Ni-O	2.7		3.75	0.04
Ni_2In_3/FTO-6M-Fe	Ni-O	2.2		3.47	0.06	Ni-O	2.1		3.74	0.05
Ni NP/FTO-6M-Fe	Ni-O	2.5		3.45	0.05	Ni-O	2.3		3.60	0.05
Ni_2In_3/FTO-p6M	Ni-In	1.3		2.60	0.01			R_f=23.6		
Ni_2In_3/FTO-6M	Ni-In	1.3		2.60	0.01			R_f=23.7		
Ni_2In_3/FTO-6M-Fe	Ni-In	1.7		2.59	0.01			R_f=19.7		
Ni NP/FTO-6M-Fe	Ni-Ni	1.2		2.66	0.02			R_f=22.8		

Table S5. Parameters used for the Ni K-edge EXAFS simulations of samples freeze-quenched at 1.2 V_{RHE} based on the model of the layered structure for metal (oxy)hydroxides comprising edge-sharing octahedra. The model does not incorporate a single Ni–In shell for Ni_2In_3-derived catalysts or an additional Ni-Ni shell for Ni NP/FTO-6M-Fe. The fit-range was 1-4 Å. Compared with Table S4, excluding the additional Ni-In or Ni-Ni coordination shell from the fitting model results in significantly higher R_f values, highlighting the necessity of including these shells and confirming the reliability and validity of our fitting approach.

**Ni K-edge at 1.2 V_{RHE}**	**Shell**	***N***	**Error**	***R* [Å]**	**Error [Å]**	**Shell**	***N***	**Error**	***R* [Å]**	**Error [Å]**
Ni_2In_3/FTO-p6M	Ni-O	2.6	0.1	1.93	0.02	Ni-O	3.4		2.09	0.01
Ni_2In_3/FTO-6M	Ni-O	2.8	0.1	1.91	0.01	Ni-O	3.2		2.09	0.01
Ni_2In_3/FTO-6M-Fe	Ni-O	3.3	0.1	1.89	0.01	Ni-O	2.7		2.08	0.02
Ni NP/FTO-6M-Fe	Ni-O	1.8	0.2	1.90	0.02	Ni-O	2.5	0.3	2.05	0.02
Ni_2In_3/FTO-p6M	Ni-Ni	2.6		2.85	0.01	Ni-Ni	3.4		3.05	0.01
Ni_2In_3/FTO-6M	Ni-Ni	2.8		2.85	0.01	Ni-Ni	3.2		3.05	0.01
Ni_2In_3/FTO-6M-Fe	Ni-Ni	3.3		2.84	0.01	Ni-Ni	2.7		3.04	0.01
Ni NP/FTO-6M-Fe	Ni-Ni	1.8		2.92	0.02	Ni-Ni	2.5		3.09	0.02
Ni_2In_3/FTO-p6M	Ni-O	2.6		3.51	0.04	Ni-O	3.4		3.75	0.03
Ni_2In_3/FTO-6M	Ni-O	2.8		3.51	0.03	Ni-O	3.2		3.77	0.03
Ni_2In_3/FTO-6M-Fe	Ni-O	3.3		3.49	0.03	Ni-O	2.7		3.92	0.04
Ni NP/FTO-6M-Fe	Ni-O	1.8		3.31	0.09	Ni-O	2.5		3.53	0.06
Ni_2In_3/FTO-p6M			R_f=32.4							
Ni_2In_3/FTO-6M			R_f=34.7							
Ni_2In_3/FTO-6M-Fe			R_f=36.1							
Ni NP/FTO-6M-Fe			R_f=35.3							

Table S6. Parameters used for the Ni K-edge EXAFS simulations of samples freeze-quenched at 1.6 V_{RHE} based on the model of the layered structure for metal (oxy)hydroxides comprising edge-sharing octahedra. The model incorporates a single Ni–In shell for Ni_2In_3-derived catalysts or an additional Ni-Ni shell for Ni NP/FTO-6M-Fe. The fit-range was 1-4 Å. The simulated curves are shown as black lines in Figure 4e and Figure S37b-c. R_f represents the fitting error in %, *N* is the coordination number (or called population) of the shell at distance *R*, and *σ* is the Debye–Waller factor. The Debye–Waller factors were fixed at 0.05 and kept constant across all fits of the nickel–iron (oxy)hydroxides to reduce the number of free parameters. The amplitude reduction factor S_0^2 was set to 0.85. The coordination numbers for the first, third, and fifth shells were constrained to be equal, representing a phase with a high nickel oxidation state and shorter bond distances. Likewise, the coordination numbers for the second, fourth, and sixth shells were constrained to be equal, corresponding to a phase with a lower nickel oxidation state and longer bond distances.

Ni K-edge at 1.6 V_{RHE}	Shell	*N*	Error	*R* [Å]	Error [Å]	Shell	*N*	Error	*R* [Å]	Error [Å]
Ni_2In_3/FTO-p6M	Ni-O	3.2	0.4	1.87	0.01	Ni-O	0.8	0.2	1.94	0.04
Ni_2In_3/FTO-6M	Ni-O	3.1	0.3	1.87	0.01	Ni-O	0.8	0.2	1.96	0.03
Ni_2In_3/FTO-6M-Fe	Ni-O	2.8	0.4	1.86	0.01	Ni-O	1.1	0.3	1.94	0.03
Ni NP/FTO-6M-Fe	Ni-O	3.6	0.6	1.87	0.01	Ni-O	1.2	0.6	1.95	0.03
Ni_2In_3/FTO-p6M	Ni-Ni	3.2		2.86	0.01	Ni-Ni	0.8		3.07	0.04
Ni_2In_3/FTO-6M	Ni-Ni	3.1		2.87	0.01	Ni-Ni	0.8		3.07	0.04
Ni_2In_3/FTO-6M-Fe	Ni-Ni	2.8		2.87	0.01	Ni-Ni	1.1		3.11	0.02
Ni NP/FTO-6M-Fe	Ni-Ni	3.6		2.79	0.01	Ni-Ni	1.2		2.89	0.03
Ni_2In_3/FTO-p6M	Ni-O	3.2		3.46	0.04	Ni-O	0.8		3.71	0.14
Ni_2In_3/FTO-6M	Ni-O	3.1		3.46	0.03	Ni-O	0.8		3.73	0.13
Ni_2In_3/FTO-6M-Fe	Ni-O	2.8		3.39	0.05	Ni-O	1.1		3.64	0.09
Ni NP/FTO-6M-Fe	Ni-O	3.6		3.37	0.03	Ni-O	1.2		3.59	0.10
Ni_2In_3/FTO-p6M	Ni-In	2.0		2.60	0.01			R_f=9.0		
Ni_2In_3/FTO-6M	Ni-In	2.1		2.59	0.01			R_f=11.0		
Ni_2In_3/FTO-6M-Fe	Ni-In	2.1		2.59	0.01			R_f=10.2		
Ni NP/FTO-6M-Fe	Ni-Ni	1.3		2.59	0.01			R_f=10.7		

Table S7. Parameters used for the Ni K-edge EXAFS simulations of samples freeze-quenched at 1.6 V_{RHE} based on the model of the layered structure for metal (oxy)hydroxides comprising edge-sharing octahedra. The model does not incorporate a single Ni–In shell for Ni_2In_3-derived catalysts or an additional Ni-Ni shell for Ni NP/FTO-6M-Fe. The fit-range was 1-4 Å. Compared with Table S6, excluding the additional Ni-In or Ni-Ni coordination shell from the fitting model results in significantly higher R_f values, highlighting the necessity of including these shells and confirming the reliability and validity of our fitting approach.

Ni K-edge at 1.6 V_{RHE}	Shell	*N*	Error	*R* [Å]	Error [Å]	Shell	*N*	Error	*R* [Å]	Error [Å]
Ni_2In_3/FTO-p6M	Ni-O	4.9	0.1	1.88	0.00	Ni-O	1.1		2.12	0.02
Ni_2In_3/FTO-6M	Ni-O	4.5	0.1	1.89	0.01	Ni-O	1.5		2.11	0.02
Ni_2In_3/FTO-6M-Fe	Ni-O	4.6	0.1	1.88	0.01	Ni-O	1.4		2.11	0.02
Ni NP/FTO-6M-Fe	Ni-O	4.3	0.1	1.91	0.01	Ni-O	1.7		2.21	0.02
Ni_2In_3/FTO-p6M	Ni-Ni	4.9		2.83	0.00	Ni-Ni	1.1		3.00	0.02
Ni_2In_3/FTO-6M	Ni-Ni	4.5		2.83	0.01	Ni-Ni	1.5		2.99	0.02
Ni_2In_3/FTO-6M-Fe	Ni-Ni	4.6		2.83	0.00	Ni-Ni	1.4		3.01	0.02
Ni NP/FTO-6M-Fe	Ni-Ni	4.3		2.84	0.01	Ni-Ni	1.7		3.02	0.01
Ni_2In_3/FTO-p6M	Ni-O	4.9		3.45	0.02	Ni-O	1.1		3.67	0.10
Ni_2In_3/FTO-6M	Ni-O	4.5		3.47	0.02	Ni-O	1.5		3.74	0.07
Ni_2In_3/FTO-6M-Fe	Ni-O	4.6		3.44	0.02	Ni-O	1.4		3.64	0.09
Ni NP/FTO-6M-Fe	Ni-O	4.3		3.47	0.03	Ni-O	1.7		3.67	0.07
Ni_2In_3/FTO-p6M			R_f=23.0							
Ni_2In_3/FTO-6M			R_f=25.8							
Ni_2In_3/FTO-6M-Fe			R_f=24.2							
Ni NP/FTO-6M-Fe			R_f=22.3							

Table S8. Parameters used for the Fe K-edge EXAFS simulations of Ni_2In_3/FTO-6M-Fe freeze-quenched at 1.6 V_{RHE} and 1.2 V_{RHE}. The fit-range was 1-4 Å. The simulated curves are shown as black lines in Figure 4f and Figure S37d. R_f represents the fitting error in %, N is the coordination number (or called population) of the shell at distance R, and σ is the Debye–Waller factor. The Debye–Waller factors were fixed at 0.05 and kept constant across all fits of the nickel–iron (oxy)hydroxides to reduce the number of free parameters. The amplitude reduction factor S_0^2 was set to 0.85. In this case, the coordination numbers were not constrained, and all shell populations were freely refined due to the lack of long-range structural order.

**Ni_2In_3/FTO-6M-Fe**	**Shell**	***N***	**Error**	***R* [Å]**	**Error [Å]**	**Shell**	***N***	**Error**	***R* [Å]**	**Error [Å]**
1.2 V_{RHE}	Fe-O	2.3	0.3	1.91	0.02	Fe-O	3.0	0.4	2.03	0.02
1.6 V_{RHE}	Fe-O	3.3	0.3	1.90	0.01	Fe-O	2.8	0.4	2.05	0.02
1.2 V_{RHE}	Fe-Fe	2.3		2.92	0.02	Fe-Fe	3.0		3.10	0.03
1.6 V_{RHE}	Fe-Fe	3.3		2.87	0.01	Fe-Fe	2.8		3.05	0.01
1.2 V_{RHE}	Fe-O	2.3		3.22	0.14	Fe-O	3.0		3.63	0.04
1.6 V_{RHE}	Fe-O	3.3		3.28	0.03	Fe-O	2.8		3.53	0.04
1.2 V_{RHE}			R_f=18.4							
1.6 V_{RHE}			R_f=14.1							

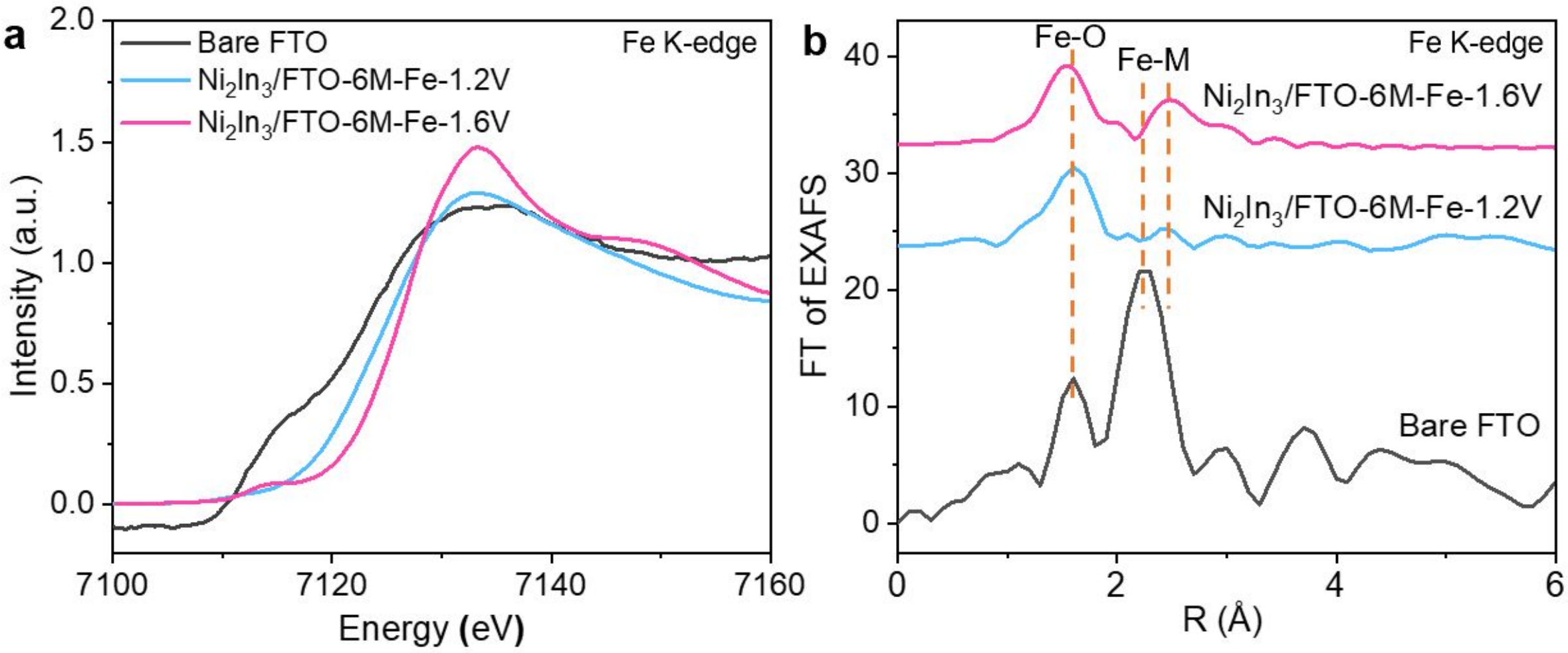

Figure S38. (a) Quasi-in-situ XANES spectra and (b) quasi-in-situ EXAFS spectra at the Fe K-edge comparing Ni_2In_3/FTO-6M-Fe with bare FTO, highlighting the differences in Fe local coordination environments.

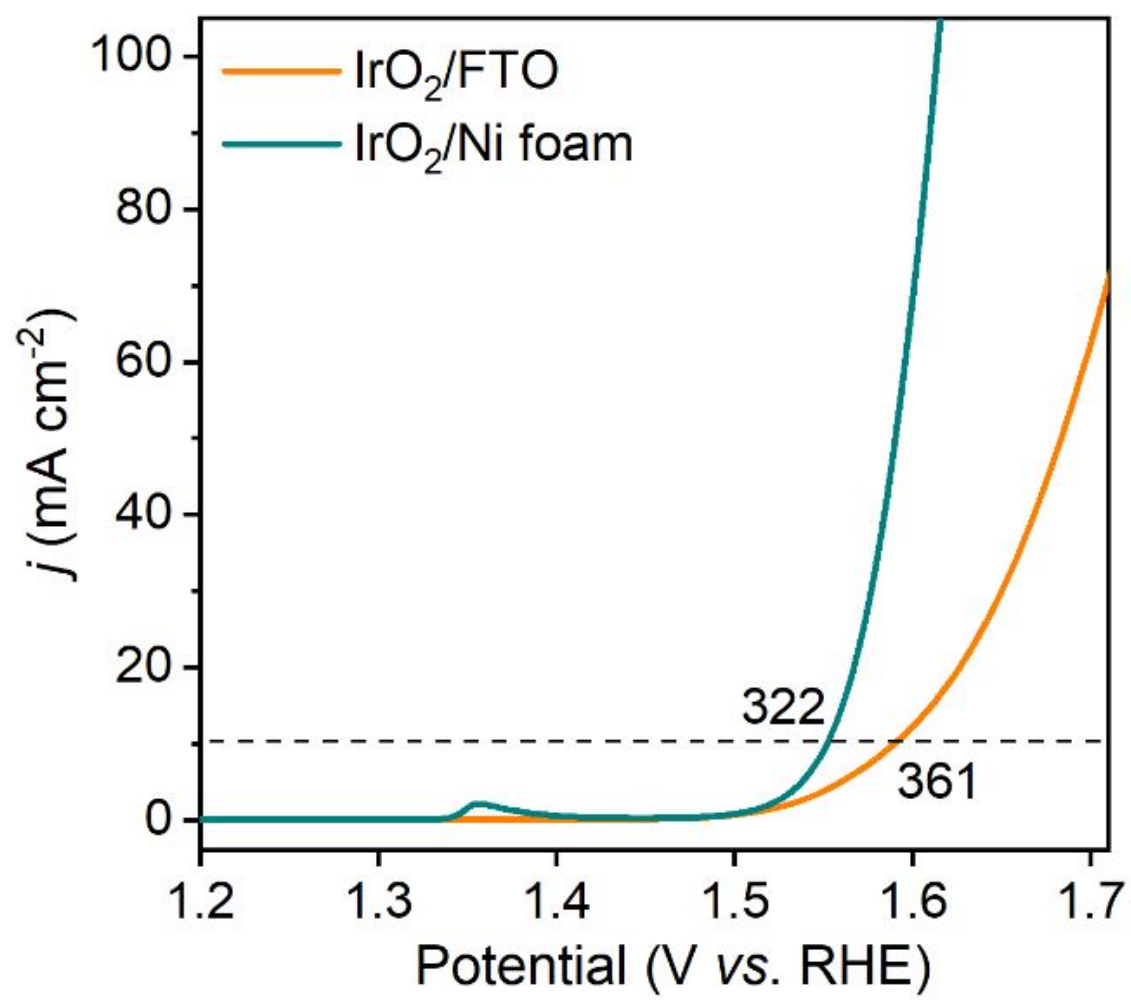

Figure S39. The LSV of commercial IrO_2 on two different substrates (FTO and Ni foam) in 1 M KOH in a three-electrode system with a scan rate of 5 mV s^{-1}. The commercial IrO_2 (Apollo Scientific, Ir 85.7%) was loaded onto two different substrates (FTO and Ni foam) with the same mass loading as the Ni_2In_3 catalyst using Nafion as binder: 0.15 mg on FTO; 1.6 mg on Ni foam.

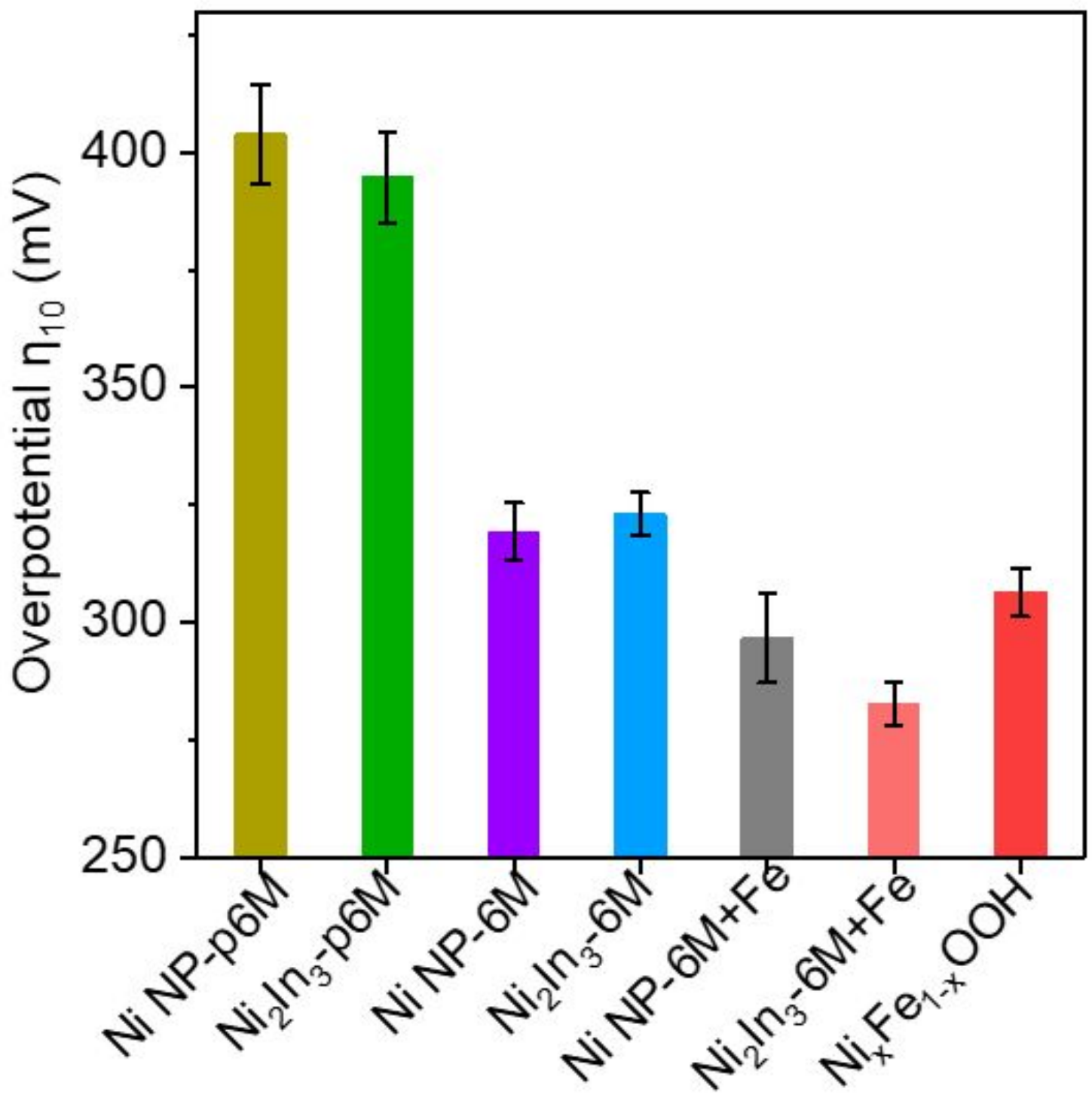

Figure S40. The overpotential with error bars at 10 mA cm^{-2} of seven catalysts tested in 1M KOH. Each sample was tested at least three times using the independently prepared films on FTO, derived from the same synthesis batch. The error bars for the overpotentials were provided based on the above-mentioned results.

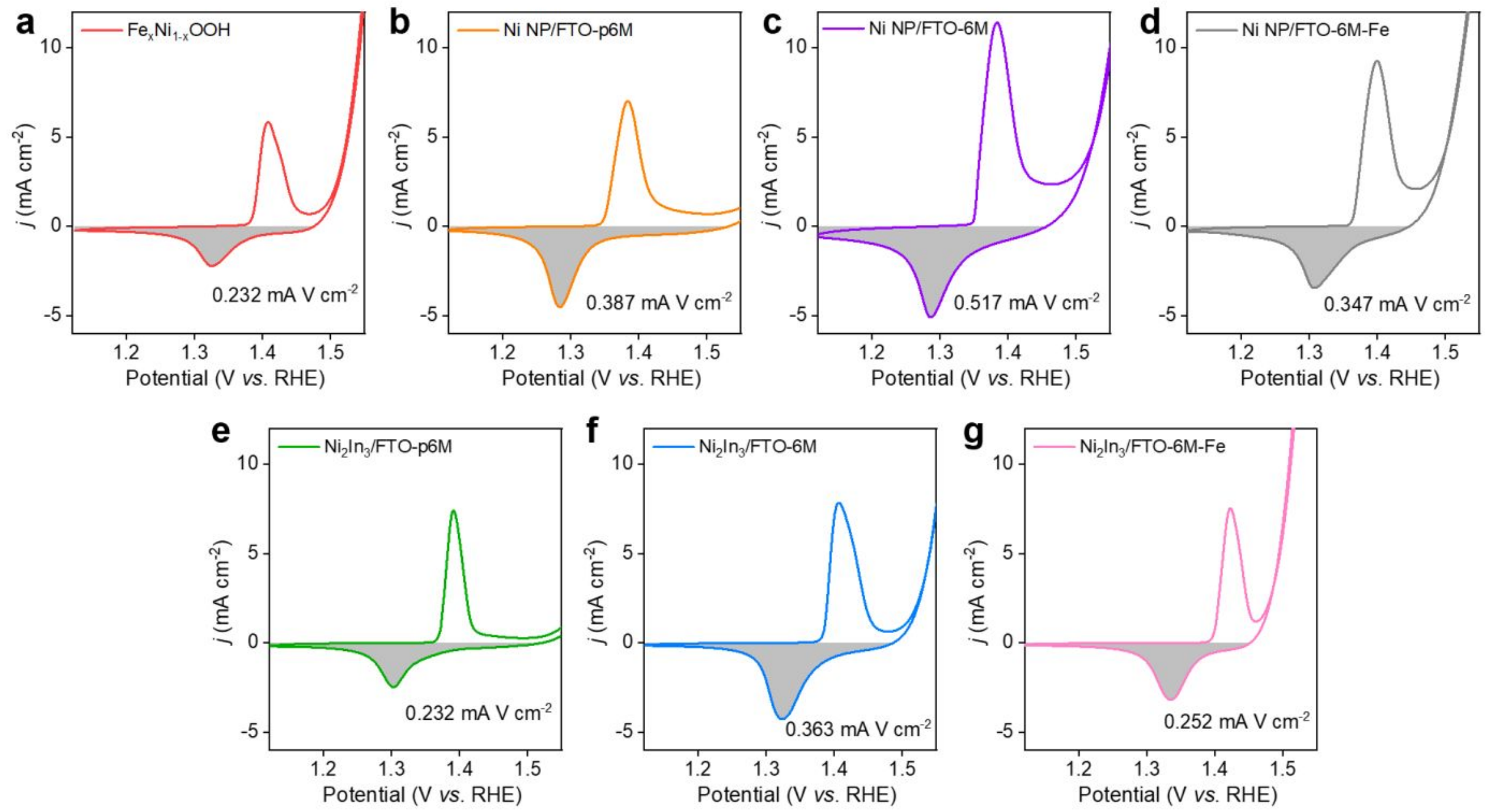

Figure S41. Integration of the reduction peaks of CV curves for (a) $Fe_xNi_{1-x}OOH$/FTO, (b) Ni NP/FTO-p6M, (c) Ni NP/FTO-6M, (d) Ni NP/FTO-6M-Fe, (e) Ni_2In_3/FTO-p6M, (f) Ni_2In_3/FTO-6M, and (g) Ni_2In_3/FTO-6M-Fe tested in 1M KOH.

Table S9. Calculation of the number of moles of electrons transferred during the reaction based on the integrated area of the reduction peak for all samples.

Samples	$\int_{V_1}^{V_2} I(E)\,dE$ (mA · V)	[e⁻] (μmol)
$Fe_xNi_{1-x}OOH$	0.232	0.481
Ni NP/FTO-p6M	0.387	0.802
Ni NP/FTO-6M	0.517	1.072
Ni NP/FTO-6M-Fe	0.347	0.715
Ni_2In_3/FTO-p6M	0.226	0.480
Ni_2In_3/FTO-6M	0.357	0.752
Ni_2In_3/FTO-6M-Fe	0.246	0.522

$$[e^-] = \frac{1}{\text{scan rate} \times F} \times \int_{V_1}^{V_2} I(E)\,dE$$

Scan rate= 5 mV/s, F=96485 s·A/mol

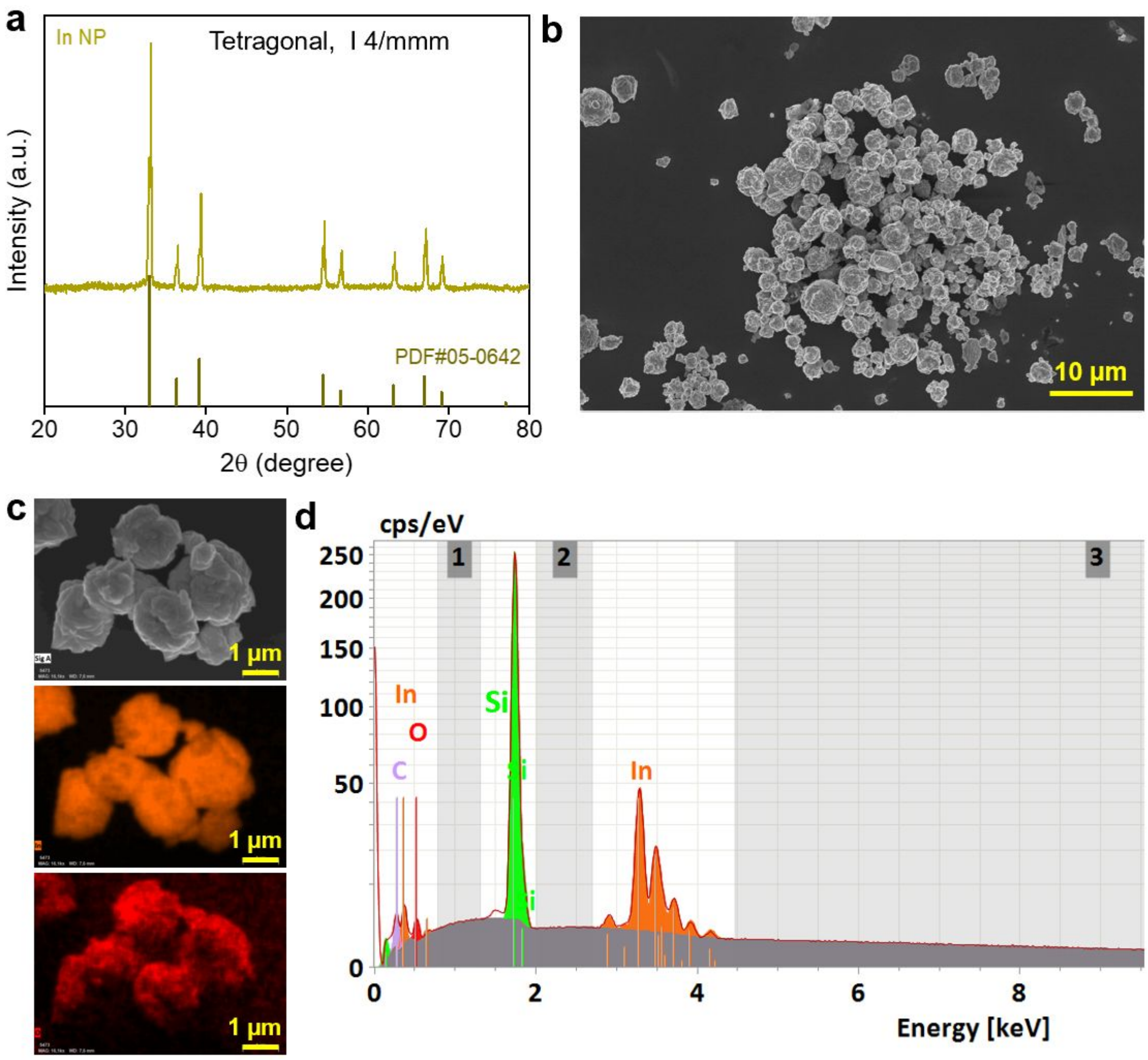

Figure S42. Structural characterization of indium nanoparticles. (a) Powder XRD, (b) SEM image, (c) elemental mapping, and (d) EDX. XRD confirms that the In nanoparticles are of a pure phase, crystallizing in the tetragonal system (PDF No. 05-0642). SEM indicates that the particles are relatively large, with sizes on the micrometer scale. Elemental mapping and EDX further verify the presence of In, with the Si signal originating from the silicon wafer. A small C signal is likely associated with surfactant residues or contamination by carbon-containing compounds, whereas the detected O signal can be attributed to partial surface oxidation of the particles.

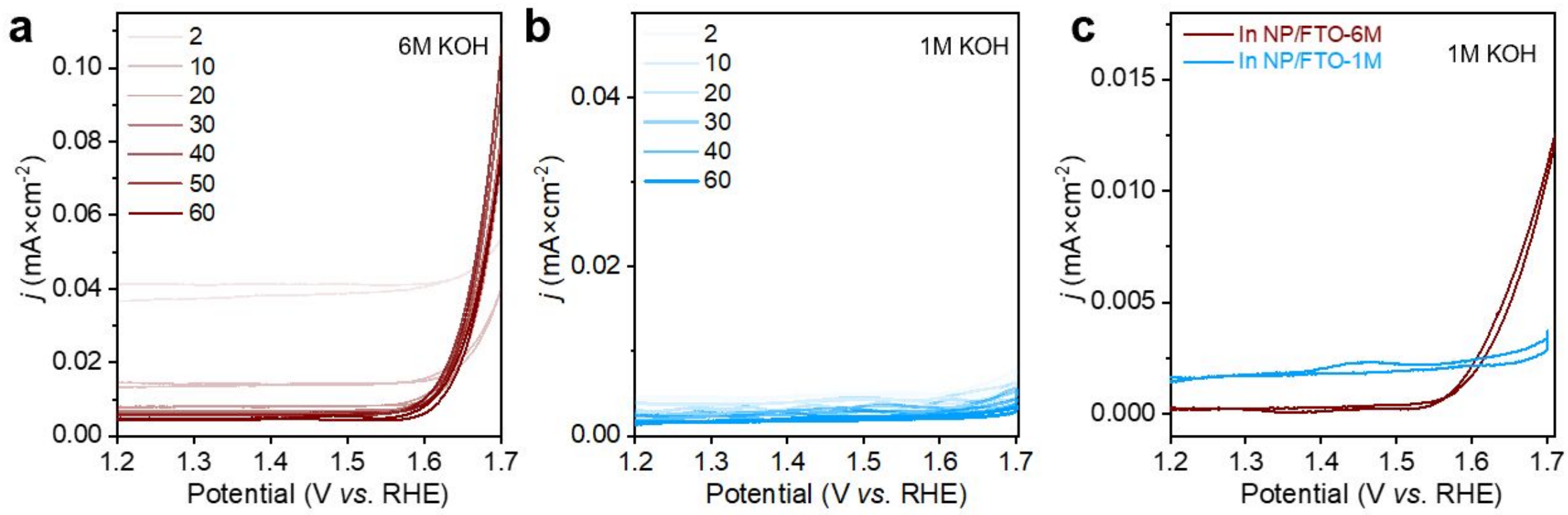

Figure S43. CV curves of In NP/FTO activated in 6M KOH (a), 1M KOH (b), and CV curves tested in 1M KOH after activation (c). The scan rate is 5mV/s.

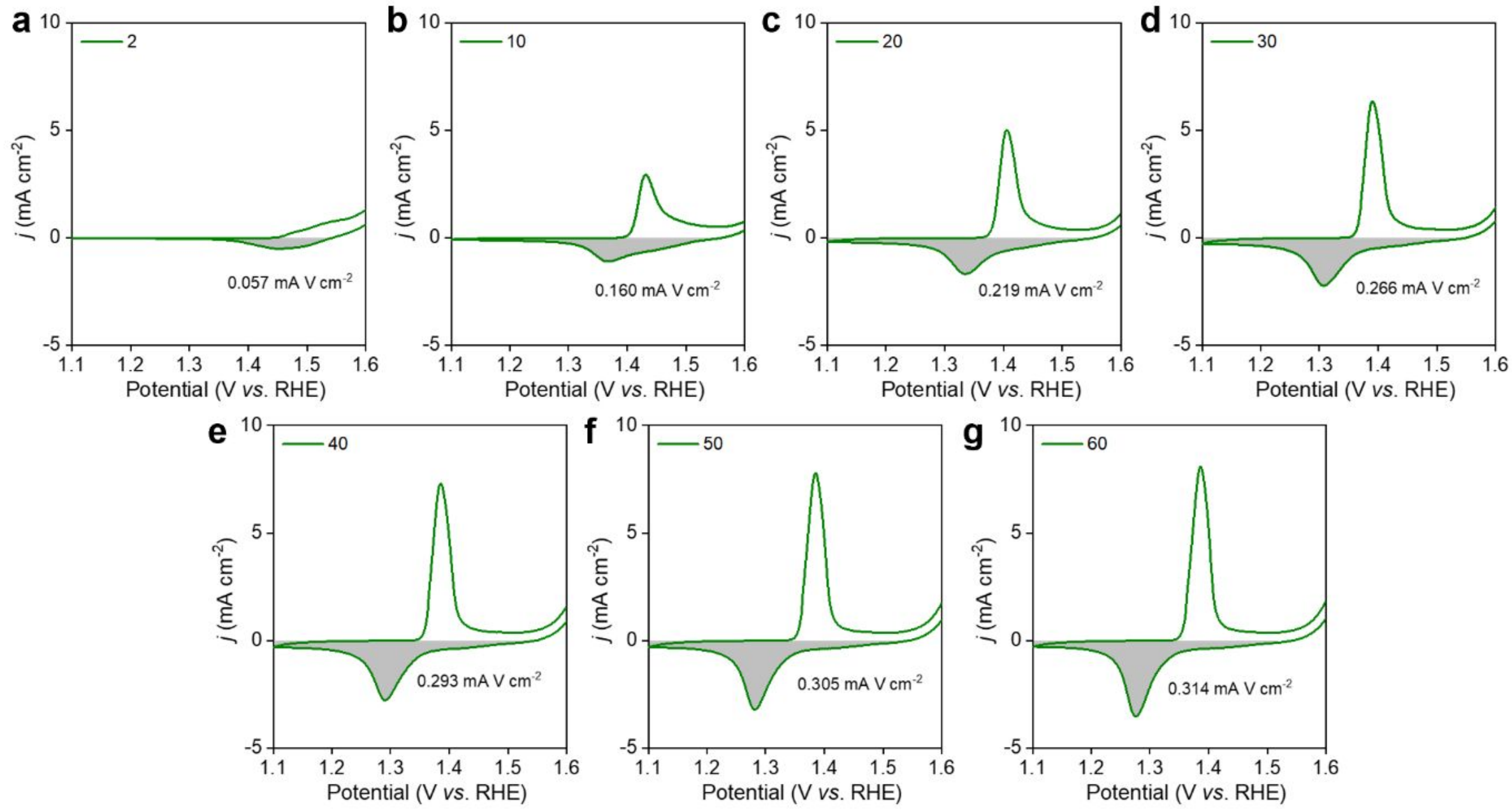

Figure S44. Integration of the reduction peaks in the 2nd, 10th, 20th, 30th, 40th, 50th, and 60th CV curves of Ni_2In_3/FTO activated in purified 6M KOH after activation. The scan rate is 5 mV/s.

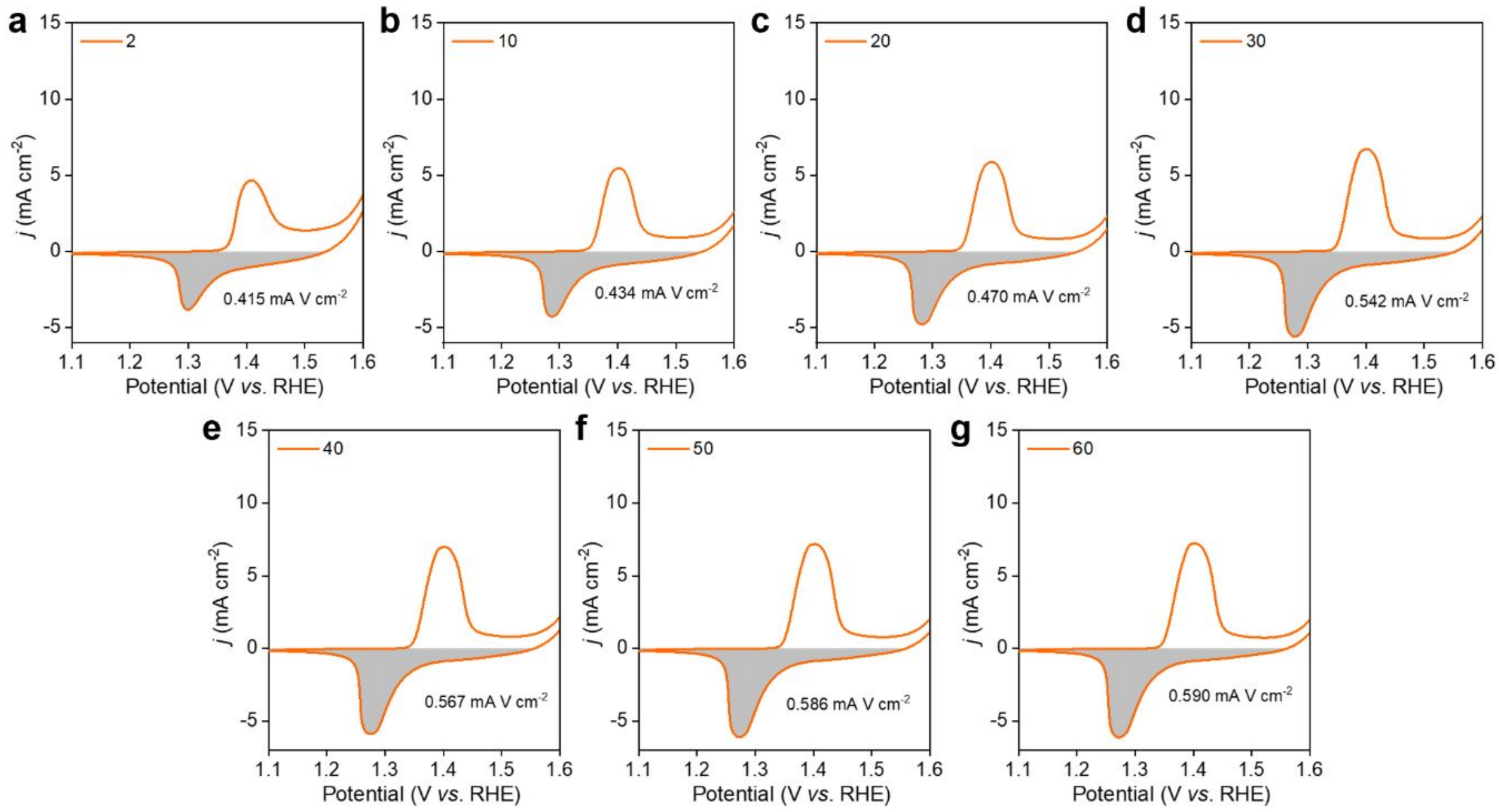

Figure S45. Integration of the reduction peaks in the 2nd, 10th, 20th, 30th, 40th, 50th, and 60th CV curves of Ni NP/FTO activated in purified 6M KOH after activation. The scan rate is 5 mV/s.

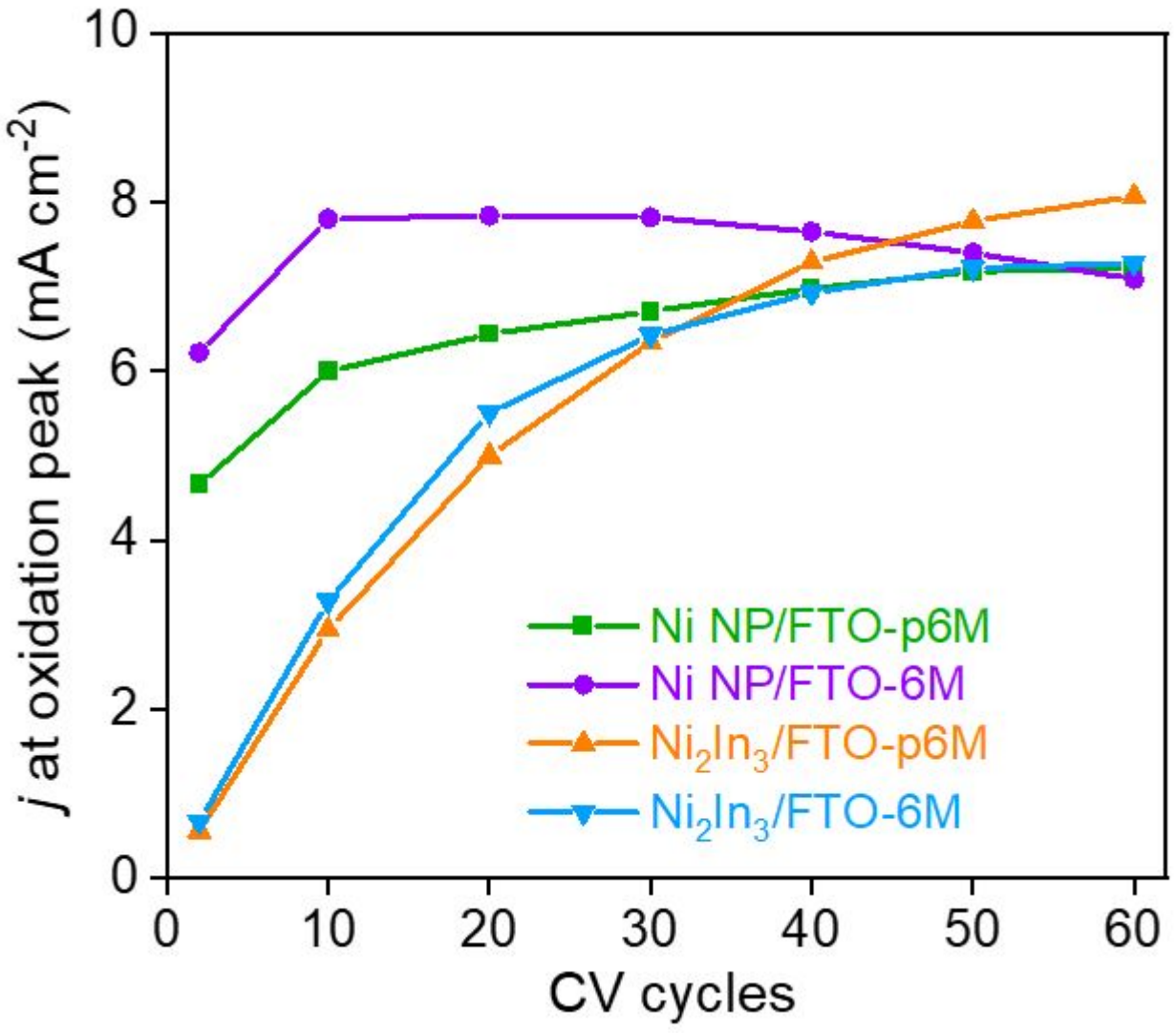

Figure S46. The current densities of Ni(II/III/IV) oxidation peak during CV cycling for Ni_2In_3/FTO and Ni NP/FTO activated in Fe-free and commercial 6M KOH, respectively.

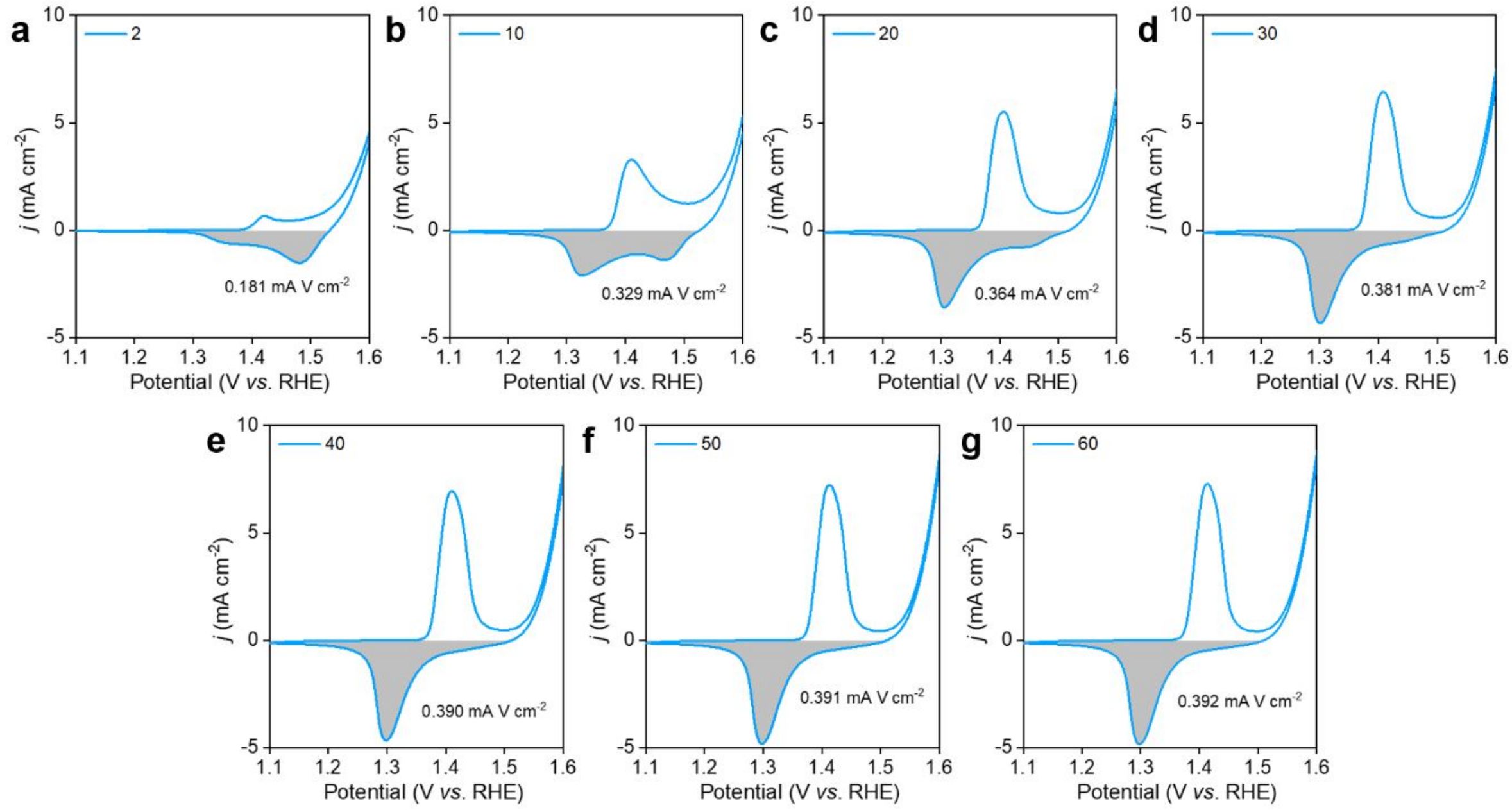

Figure S47. Integration of the reduction peaks in the 2nd, 10th, 20th, 30th, 40th, 50th, and 60th CV curves of Ni_2In_3/FTO activated in 6M KOH. The scan rate is 5 mV/s.

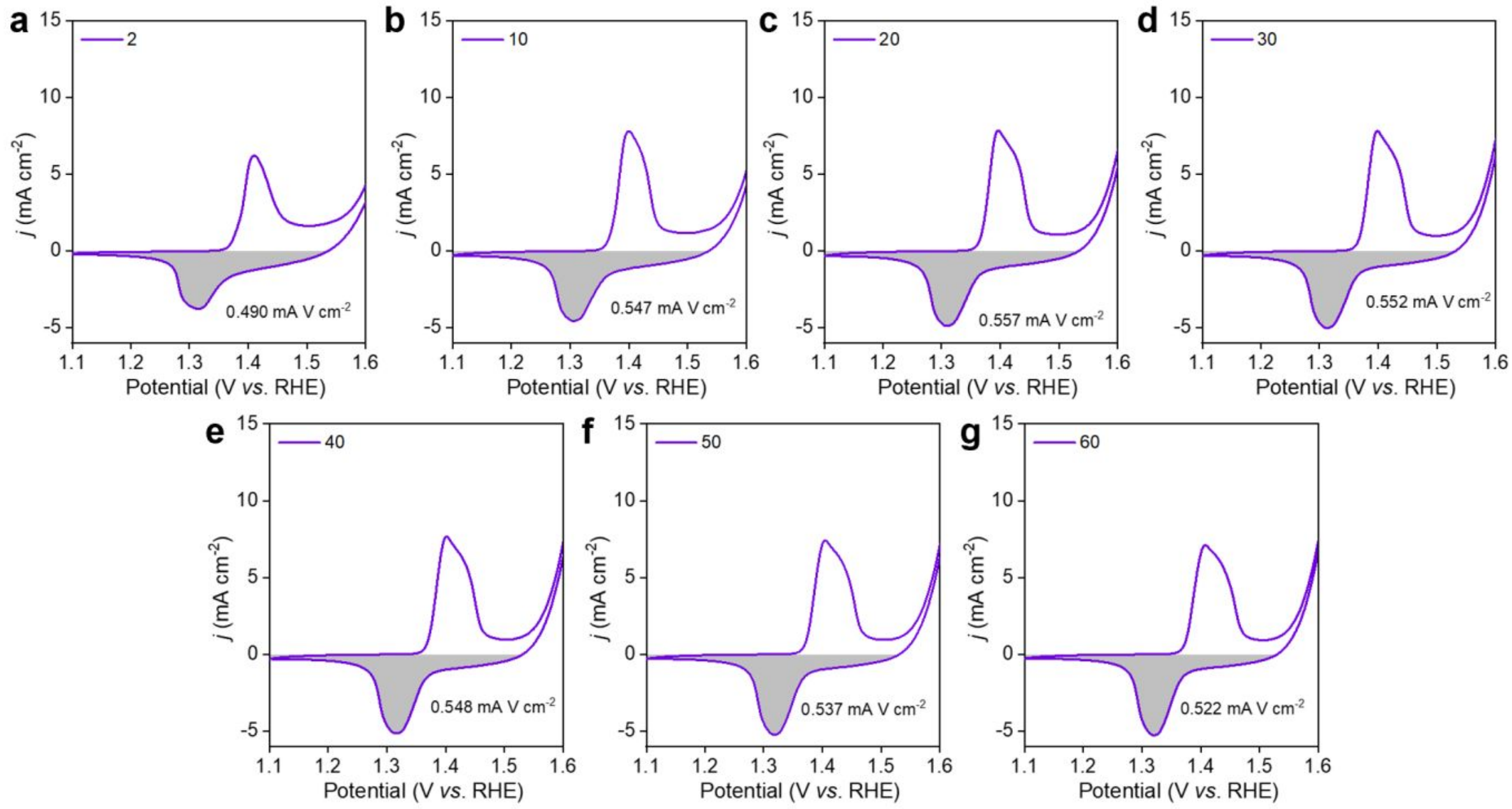

Figure S48. Integration of the reduction peaks in the 2nd, 10th, 20th, 30th, 40th, 50th, and 60th CV curves of Ni NP/FTO activated in 6M KOH after activation. The scan rate is 5 mV/s.

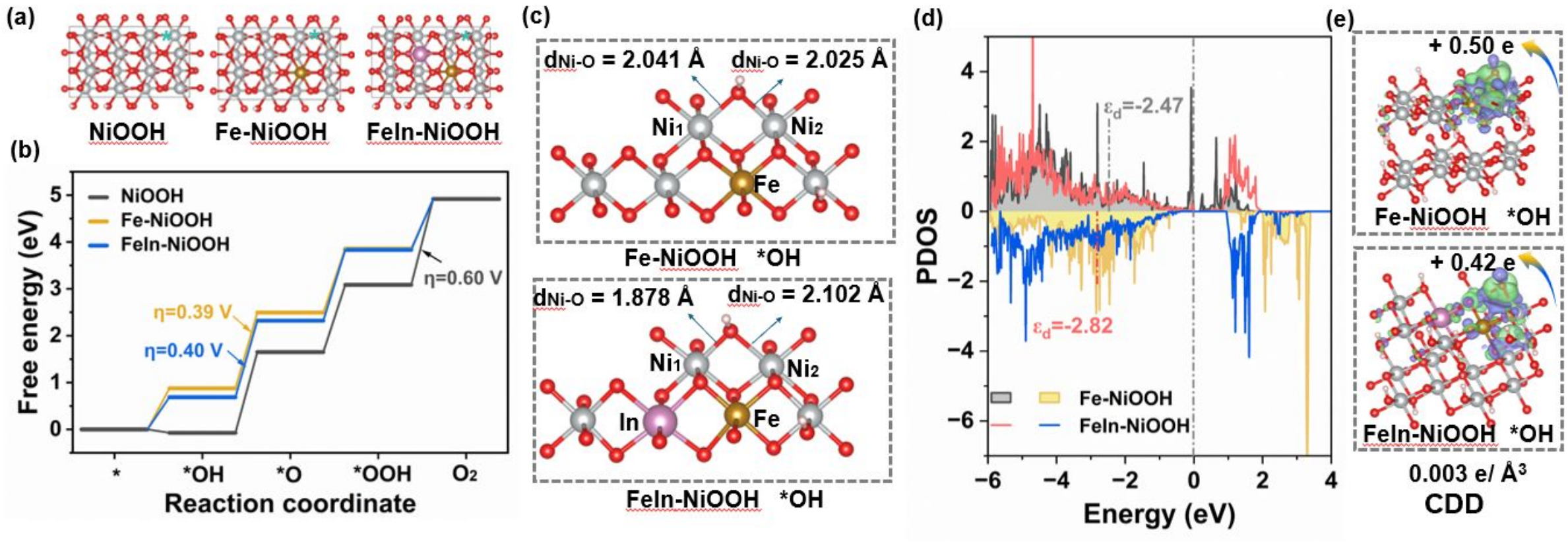

Figure S49. (a) Geometric structures of the constructed NiOOH, Fe doped NiOOH (Fe-NiOOH), and Fe, In co-doped NiOOH (FeIn-NiOOH) catalysts, where the cyan asterisk (*) denotes the active sites. (b) Free energy profiles for the OER process. (c) Local atomic configurations and the corresponding Ni–O bond lengths for *OH adsorption on the Fe-NiOOH and FeIn-NiOOH surfaces. (d) Projected density of states (PDOS) for the 3d orbitals of the Ni atom directly coordinated to the *OH intermediate in the *OH-adsorbed configuration. (e) Charge density difference (CDD) distributions for Fe-NiOOH and FeIn-NiOOH with *OH adsorption. The isosurface contour is set at 0.003 e/Å3, with green and purple regions indicating electron accumulation and depletion, respectively.

Based on the *in situ* Raman and XAS results, the reconstructed active phase predominantly exhibits the characteristic features of a Ni oxyhydroxide-like structure. Accordingly, a γ-NiOOH (0001) surface was adopted as the structural framework for theoretical modeling. A two-layer slab containing 16 Ni atoms was first constructed as the reference model (denoted NiOOH). Considering the structural complexity of the actual catalysts, simplified Fe-doped NiOOH (Fe–NiOOH) and Fe/In co-doped NiOOH (FeIn–NiOOH) models were constructed to represent conventional NiFeOOH and the reconstructed Ni_2In_3/FTO-6M-Fe catalyst, respectively, as depicted in **Figure S49a**.

To further elucidate their difference in catalytic activity, the corresponding Gibbs free energy profiles are illustrated in **Figure S49b**. The theoretical overpotential calculations reveal that pristine NiOOH exhibits a high overpotential of 0.60 V, originating from the excessively strong adsorption of oxygenated intermediates, particularly *OH, which renders the final step (*OOH → O_2) the potential-determining step (PDS). Fe incorporation markedly enhances the OER activity by lowering the overpotential to 0.39 V, with the *OH → *O deprotonation step becoming the PDS. Notably, FeIn–NiOOH exhibits a comparable overpotential of 0.40 V, suggesting that the presence of In preserves the favorable energetics induced by Fe but does not

provide an appreciable additional enhancement in intrinsic OER activity within the present model.

On the Fe-NiOOH surface, the *OH intermediate adopts a highly symmetric bridging coordination, with nearly identical Ni-O bond lengths of 2.041 and 2.045 Å (**Figure S49c**). Upon In incorporation, the Ni_1-O bond undergoes a pronounced local contraction to 1.878 Å, providing direct geometric evidence of the strengthened interaction between the active site and the OH adsorbate. To uncover the electronic mechanism driving this geometric behavior, the projected density of states (PDOS) and charge density difference (CDD) were analyzed (**Figure S49d and S49e**). The incorporation of In modifies the local electronic structure of the neighboring Ni atoms, which downshifts the Ni d-band center from −2.47 eV in Fe-NiOOH to −2.82 eV in FeIn-NiOOH, accompanied by a reduced total charge transfer (0.40 e) to the *OH adsorbate. Although the downshifted Ni d-band center and reduced charge transfer might conventionally suggest weakened adsorption, the pronounced Ni_1–O bond contraction indicates that In incorporation instead redistributes the metal–adsorbate interaction into a more localized coordination environment. This localized orbital coupling strengthens the Ni_1–O bond, thereby reconciling the geometric and electronic characteristics of the FeIn-NiOOH surface.

Therefore, these calculations, along with other characterizations, suggest that In leaching during reconstruction modifies the Ni electronic structure and exposes additional interfacial sites for Fe incorporation, which enhances the intrinsic OER activity. Meanwhile, the residual In may continue to regulate the local electronic environment of Ni but does not appear to provide any further enhancement in intrinsic activity.

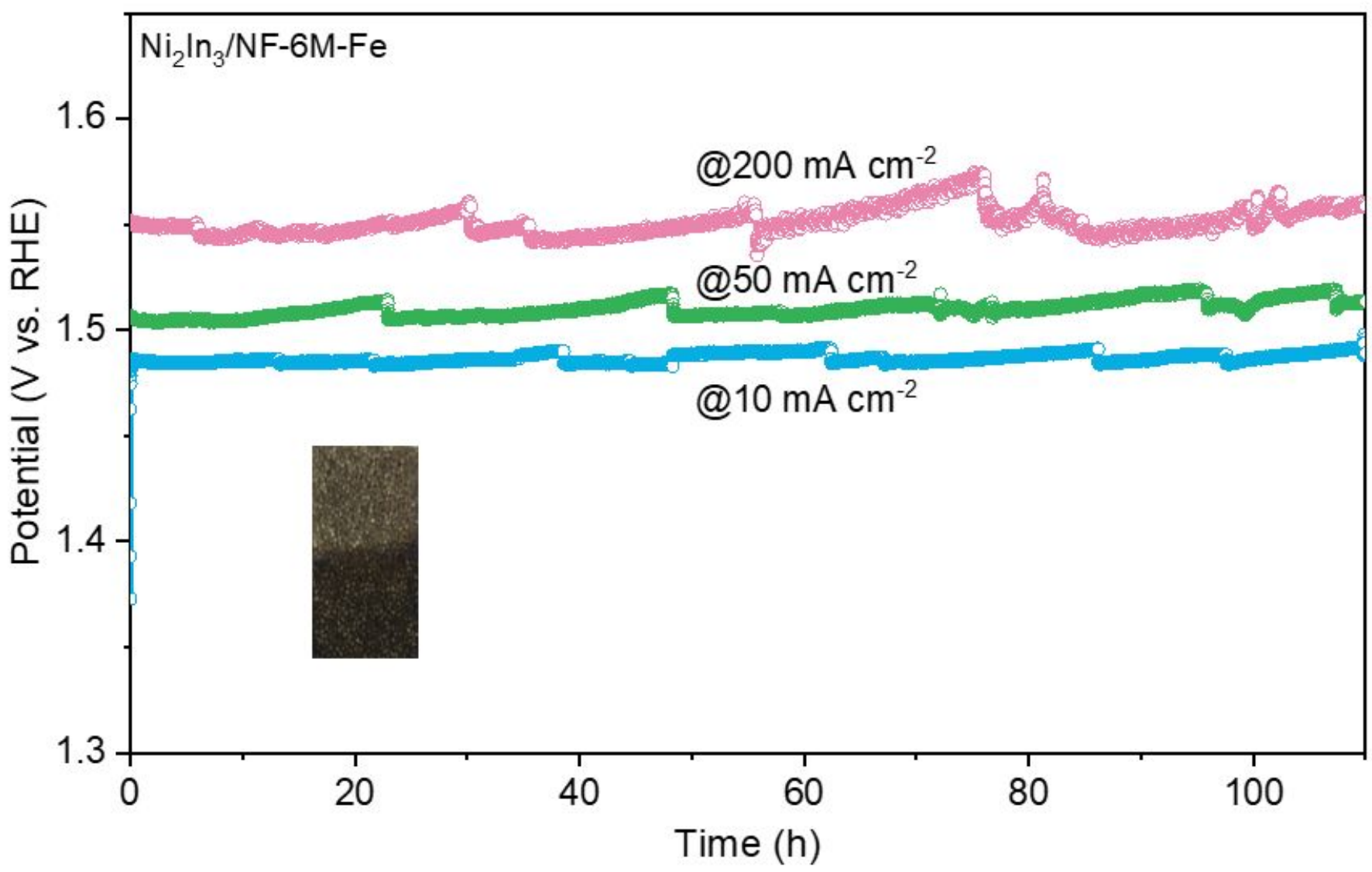

Figure S50. The stability test of Ni_2In_3/NF-6M-Fe via chronopotentiometry in a three-electrode system in 1M KOH at 25 ^oC at 10, 50, and 200 mA cm^{-2}, respectively.

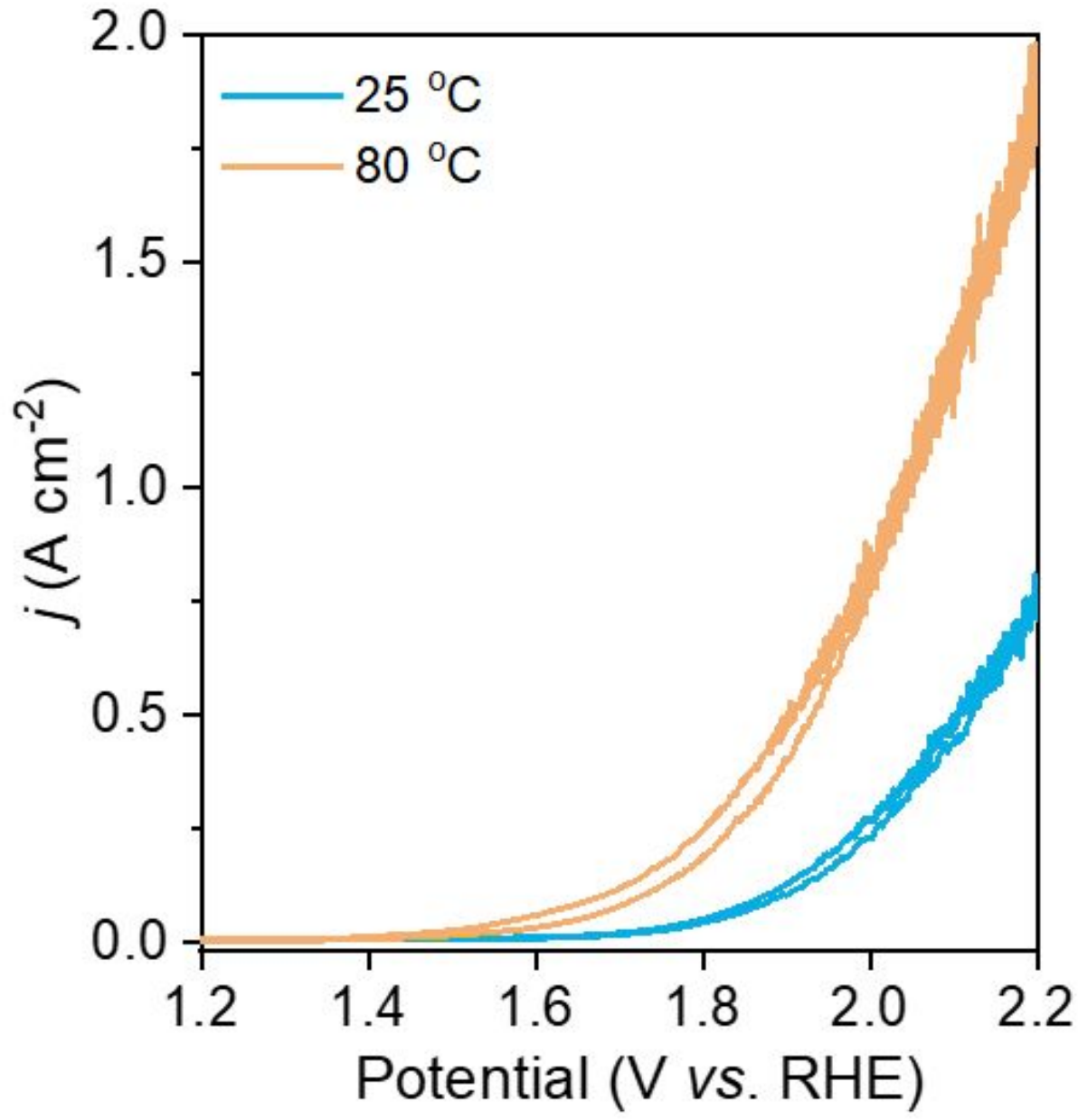

Figure S51. The CV curves at different temperatures in the AEMWE cell with Ni_2In_3/NF-6M-Fe as anode and Ni_4Mo/MoO_2@NF as cathode.

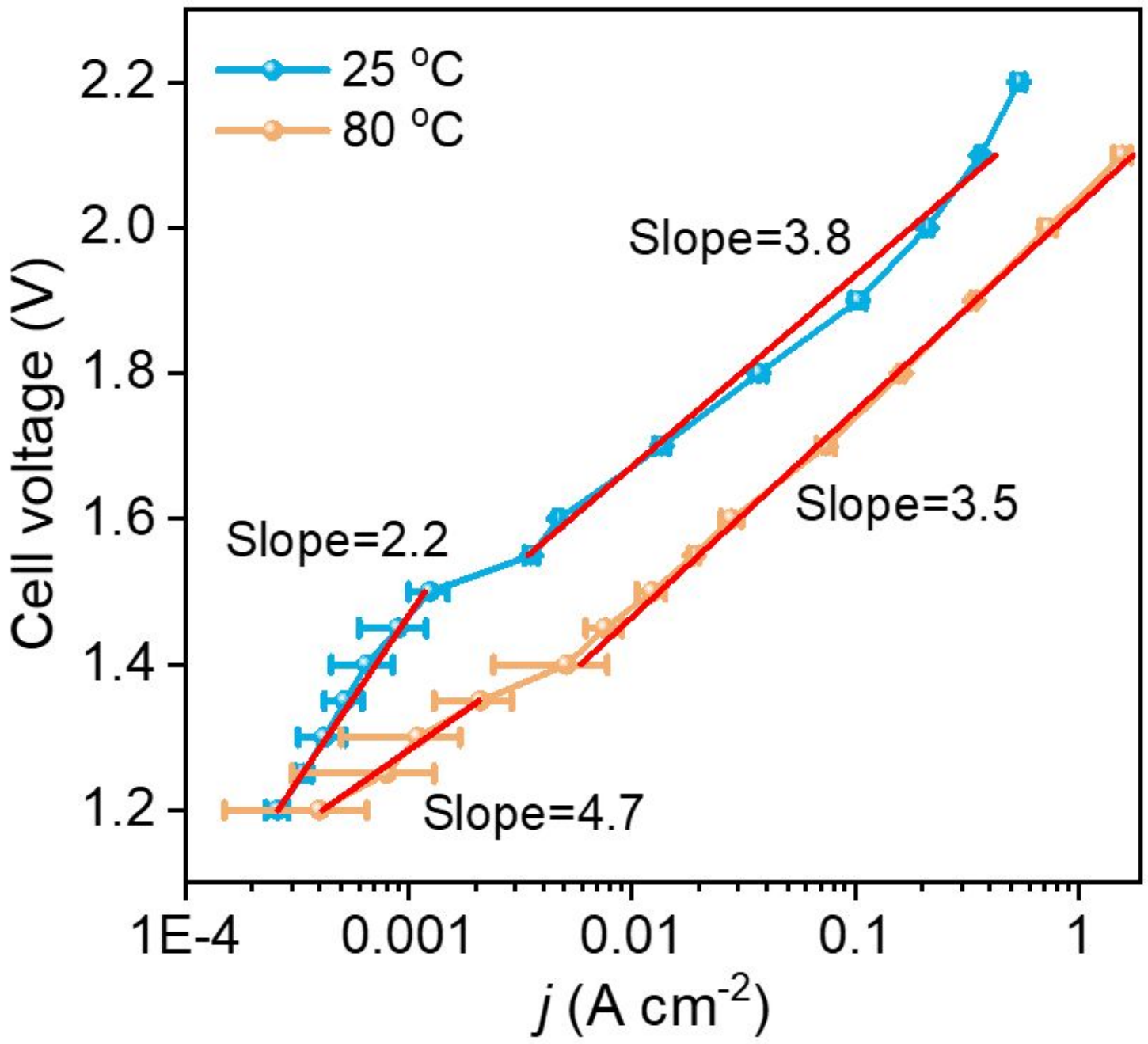

Figure S52. The Polarization curves with error bars based on step-wise chronoamperometry at different temperatures. The initial slopes of the polarization curves exhibit a noticeable difference between 25 °C (slope = 2.2) and 80 °C (slope = 4.7), indicating that the initial part is temperature-dependent. In contrast, the slopes of different temperatures at high current densities (3.8 and 3.5) remain nearly unchanged, suggesting that the intrinsic catalytic kinetics are only weakly affected by temperature.

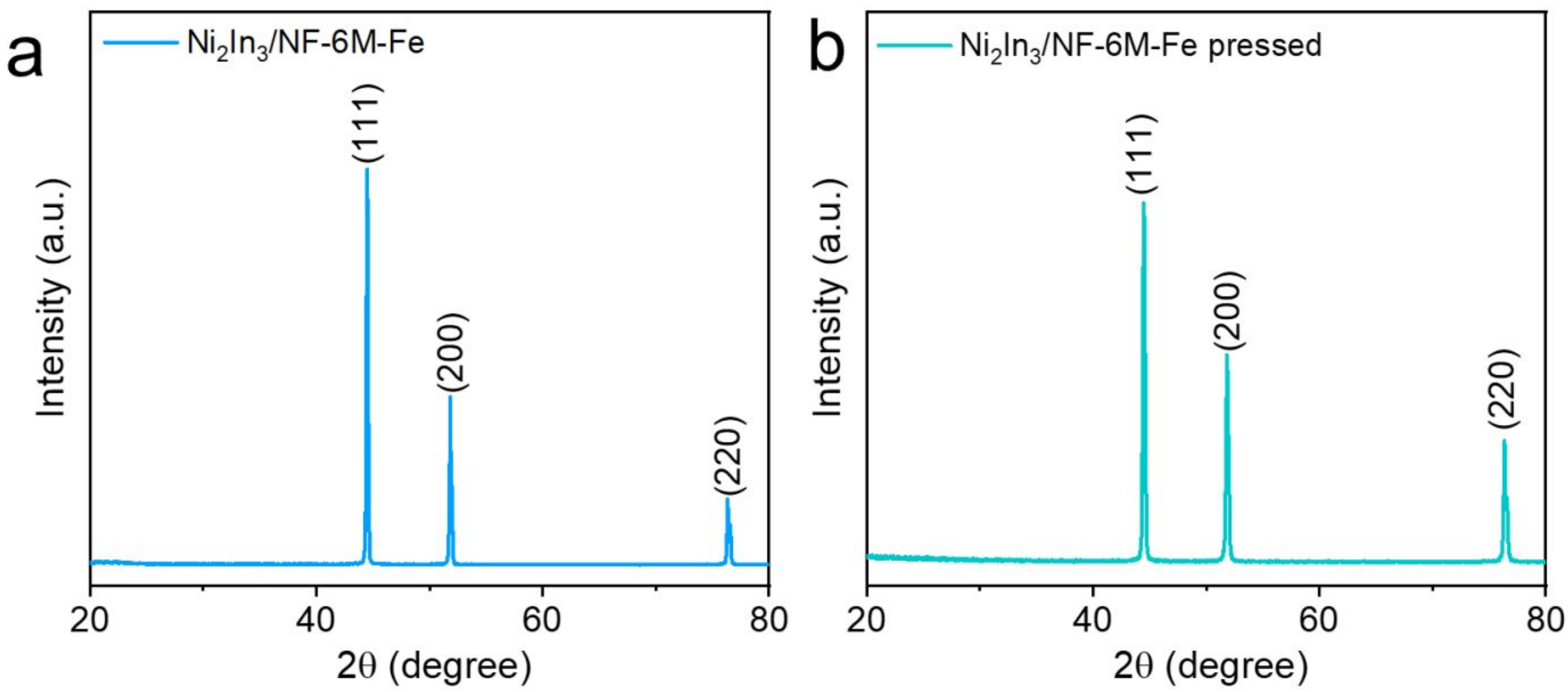

Figure S53. The GIXRD of Ni_2In_3/NF-6M-Fe after the long-term stability test in the AEMWE cell. (a) Only three distinct diffraction peaks corresponding to metallic Ni foam were observed, which can be indexed to the (111), (200), and (220) planes (PDF No. 65-0830). The highly rough and porous surface of the Ni foam hinders signal collection in GIXRD measurements. (b) To minimize this effect, the Ni foam was mechanically flattened prior to measurement. However, no additional diffraction peaks were detected.

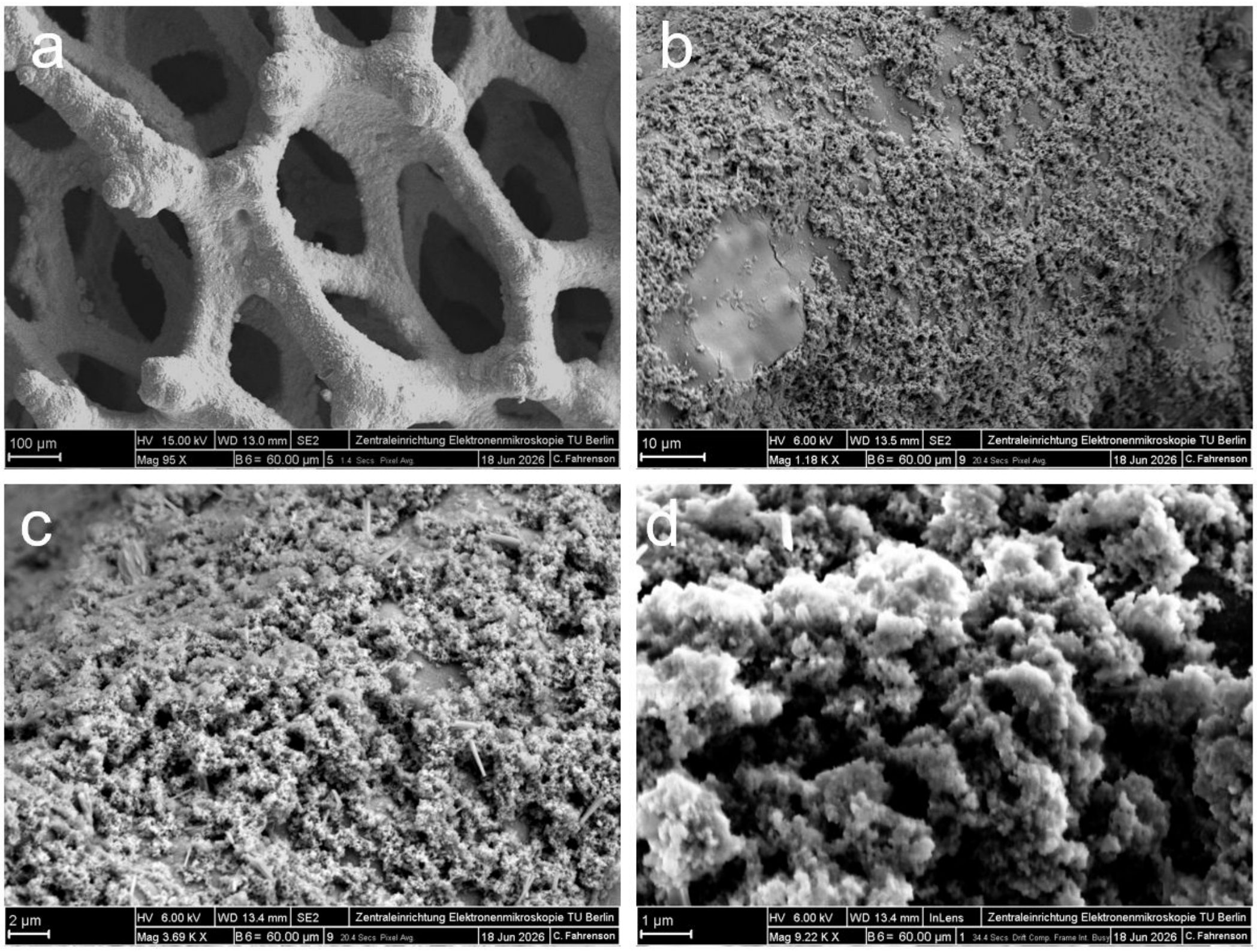

Figure S54. (a-d) The SEM images of Ni_2In_3/NF-6M-Fe after the long-term OER stability test in the AEMWE cell. It can be seen that a catalyst layer still remained on the surface of the Ni foam after the long-term stability test, exhibiting a morphology similar to that before the test (Figure S27). This implies that the catalyst possesses favorable structural and mechanical stability under long-term OER stability testing.

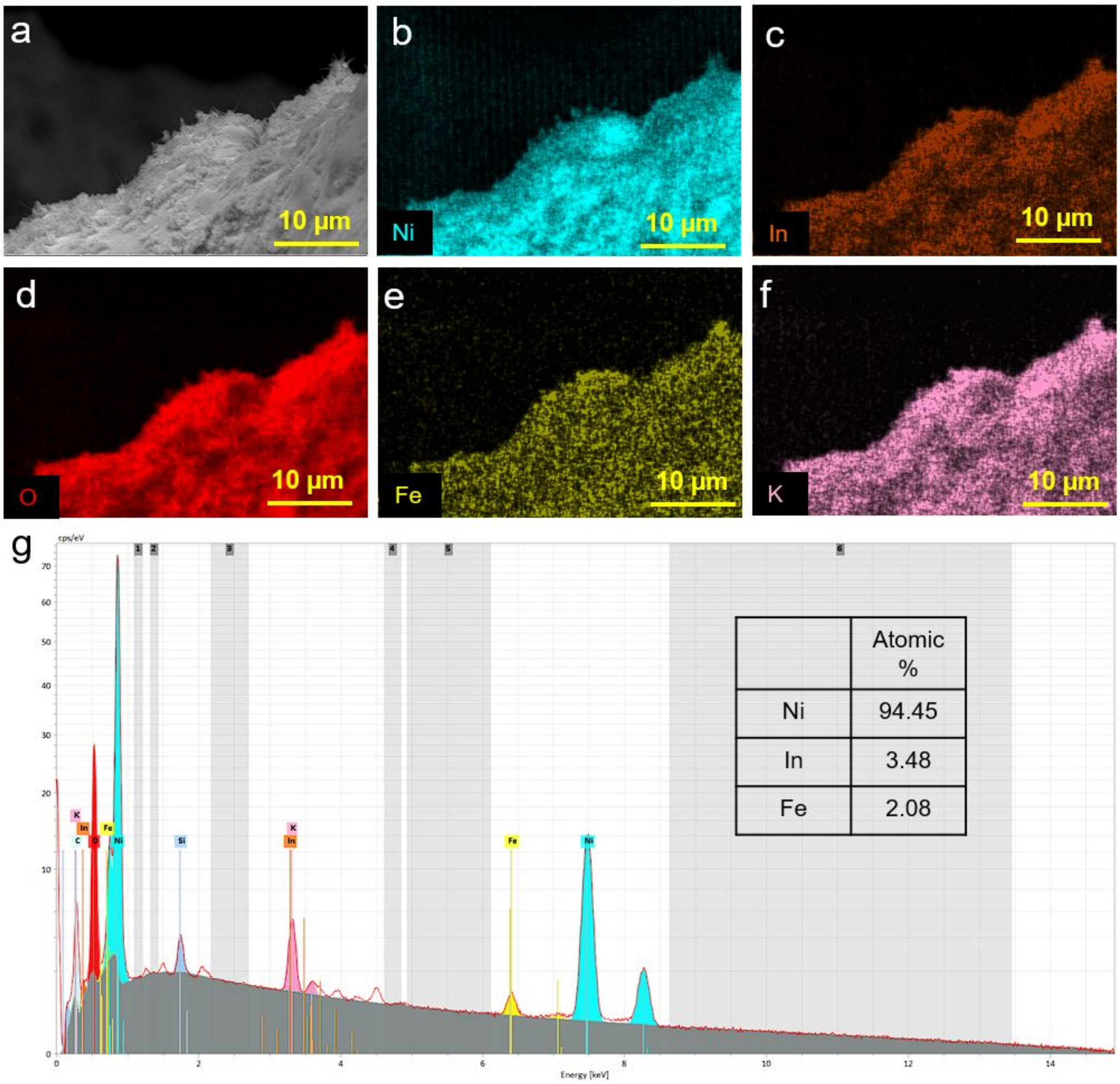

Figure S55. (a-f) elemental mapping and (g) EDX of Ni_2In_3/NF-6M-Fe after the long-term stability test in the AEMWE cell. The EDX spectra confirm the presence of Ni, Fe, In, and O. The K signal is from the residual electrolyte (KOH). The Si signal is from the silicon wafer.

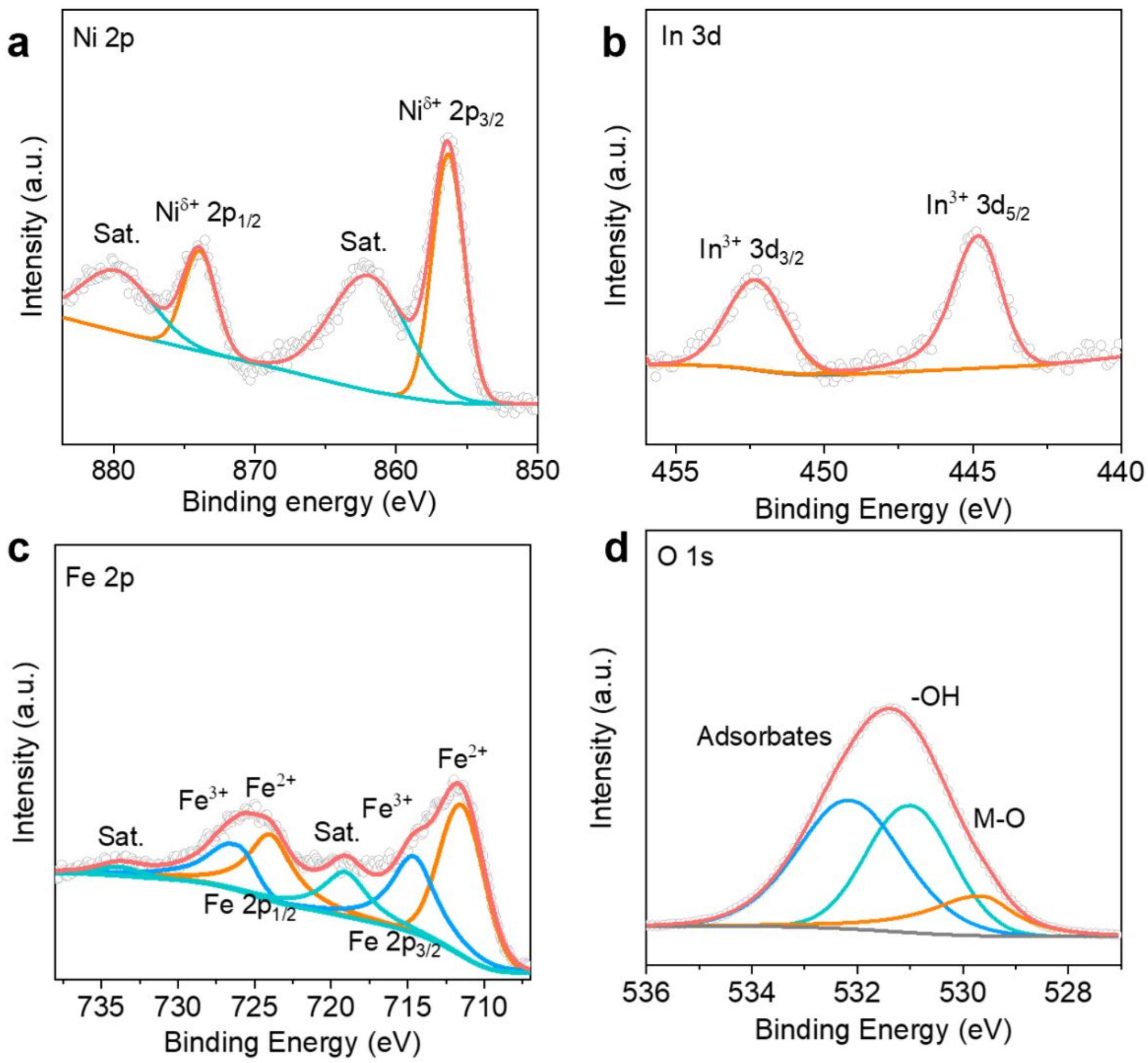

Figure S56. The XPS spectra of (a) Ni 2p, (b) In 3d, (c) Fe 2p and (d) O 1s for Ni_2In_3/NF-6M-Fe after the long-term stability test in the AEMWE cell. The peaks located at about 856.3 and 873.8 eV are assigned to $Ni^{\delta+}$, whereas those at about 862.0and 878.8 eV are attributed to satellite peaks.[4, 15] Of note, part of the detected Ni signal may originate from the Ni foam substrate. Importantly, the In 3d signals remain clearly detectable and are assigned to In^{3+}, demonstrating that In is not completely leached during long-term operation.[16, 19] An additional 100 scans were collected for the Fe 2p region compared to 25 scans for other elements among all samples; Fe signals correspond to Fe^{2+} and Fe^{3+}.[20, 21]

References

(1) Corrigan, D. A.; Bendert, R. M. Effect of Coprecipitated Metal Ions on the Electrochemistry of Nickel Hydroxide Thin Films: Cyclic Voltammetry in 1M KOH. *J. Electrochem. Soc.* **1989**, *136*, 723–728.

(2) Mondal, I.; Hausmann, J. N.; Mebs, S.; Kalra, S.; Vijaykumar, G.; Laun, K.; Zebger, I.; Selve, S.; Dau, H.; Driess, M.; Menezes, P. W. The (In)Stability of Heterostructures During the Oxygen Evolution Reaction. *Adv. Energy Mater.* **2024**, *14*, 2400809.

(3) Risch, M.; Ringleb, F.; Kohlhoff, M.; Bogdanoff, P.; Chernev, P.; Zaharieva, I.; Dau, H. Water Oxidation by Amorphous Cobalt-Based Oxides: In Situ Tracking of Redox Transitions and Mode of Catalysis. *Energy Environ. Sci.* **2015**, *8*, 661–674.

(4) Dasgupta, B.; Yao, S.; Mondal, I.; Mebs, S.; Schmidt, J.; Laun, K.; Zebger, I.; Dau, H.; Driess, M.; Menezes, P. W. A Knowledge-Based Molecular Single-Source Precursor Approach to Nickel Chalcogenide Precatalysts for Electrocatalytic Water, Alcohol, and Aldehyde Oxidations. *ACS Nano* **2024**, *18*, 33964–33976.

(5) Menezes, P. W.; Yao, S.; Beltrán-Suito, R.; Hausmann, J. N.; Menezes, P. V.; Driess, M. Facile Access to an Active γ-NiOOH Electrocatalyst for Durable Water Oxidation Derived from an Intermetallic Nickel Germanide Precursor. *Angew. Chem. Int. Ed.* **2021**, *60*, 4640–4647.

(6) Das, K.; Hausmann, J. N.; Driess, M.; Menezes, P. W. Toward Reliable Reference Electrode Calibration in Alkaline Solution. *ACS Energy Lett.* **2025**, *10*, 3269–3274.

(7) Zhang, J.; Wang, T.; Liu, P.; Liao, Z.; Liu, S.; Zhuang, X.; Chen, M.; Zschech, E.; Feng, X. Efficient Hydrogen Production on $MoNi_4$ Electrocatalysts with Fast Water Dissociation Kinetics. *Nat. Commun.* **2017**, *8*, 15437.

(8) Chung, D. Y.; Lopes, P. P.; Farinazzo Bergamo Dias Martins, P.; He, H.; Kawaguchi, T.; Zapol, P.; You, H.; Tripkovic, D.; Strmcnik, D.; Zhu, Y.; Seifert, S.; Lee, S.; Stamenkovic, V. R.; Markovic, N. M. Dynamic Stability of Active Sites in Hydr(Oxy)Oxides for the Oxygen Evolution Reaction. *Nat. Energy* **2020**, *5*, 222–230.

(9) Kresse, G.; Furthmüller, J. Efficient Iterative Schemes for *Ab Initio* Total-Energy Calculations Using a Plane-Wave Basis Set. *Phys. Rev. B* **1996**, *54*, 11169–11186.

(10) Perdew, J. P.; Burke, K.; Ernzerhof, M. Generalized Gradient Approximation Made Simple. *Phys. Rev. Lett.* **1996**, *77*, 3865–3868.

(11) Grimme, S.; Antony, J.; Ehrlich, S.; Krieg, H. A Consistent and Accurate *Ab Initio* Parametrization of Density Functional Dispersion Correction (DFT-D) for the 94 Elements H–Pu. *J. Chem. Phys.* **2010**, *132*, 154104.

(12) Grimme, S.; Ehrlich, S.; Goerigk, L. Effect of the Damping Function in Dispersion-Corrected Density Functional Theory. *J. Comput. Chem.* **2011**, *32*, 1456–1465.

(13) Chen, D.; Perilli, D.; Dronskowski, R.; Selloni, A.; Di Valentin, C. Enhanced Oxygen Evolution on NiOOH through Fe-Promoted Transformation of β to γ Phase. *Acta Mater.* **2025**, *299*, 121434.

(14) Hausmann, J. N.; Ashton, M.; Mebs, S.; Walter, C.; Selve, S.; Haumann, M.; Sontheimer, T.; Dau, H.; Driess, M.; Menezes, P. W. Intermetallic Cobalt Indium Nanoparticles as Oxygen Evolution Reaction Precatalyst: A Non-Leaching p-Block Element. *Small* **2024**, *20*, 2309749.

(15) Hengne, A. M.; Samal, A. K.; Enakonda, L. R.; Harb, M.; Gevers, L. E.; Anjum, D. H.; Hedhili, M. N.; Saih, Y.; Huang, K. W.; Basset, J. M. Ni–Sn-Supported ZrO_2 Catalysts Modified by Indium for Selective CO_2 Hydrogenation to Methanol. *ACS Omega* **2018**, *3*, 3688–3701.

(16) Henderson, J. D.; Pearson, L.; Nie, H. Y.; Biesinger, M. C. X-Ray Photoelectron Spectroscopy Analysis of Indium and Indium-Containing Compounds. *Surf. Interface Anal.* **2025**, *57*, 81–97.

(17) Thacharon, A.; Jang, W. S.; Kim, J.; Kang, J.; Kim, Y. M.; Kim, S. W. Non-Oxidized Bare Metal Nanoparticles in Air: A Rational Approach for Large-Scale Synthesis via Wet Chemical Process. *Adv. Sci.* **2022**, *9*, 2201756.

(18) Schweitzer, G. K.; Pesterfield, L. L. *The Aqueous Chemistry of the Elements*. Oxford University Press: New York, 2010; pp 163–164.

(19) Fu, G.; Wang, J.; Chen, Y.; Liu, Y.; Tang, Y.; Goodenough, J. B.; Lee, J. M. Exploring Indium-Based Ternary Thiospinel as Conceivable High-Potential Air-Cathode for Rechargeable Zn–Air Batteries. *Adv. Energy Mater.* **2018**, *8*, 1802263.

(20) Li, L.; Ma, P.; Hussain, S.; Jia, L.; Lin, D.; Yin, X.; Lin, Y.; Cheng, Z.; Wang, L. FeS_2/Carbon Hybrids on Carbon Cloth: A Highly Efficient and Stable Counter Electrode for Dye-Sensitized Solar Cells. *Sustain. Energy Fuels* **2019**, *3*, 1749–1756.

(21) Zhang, G.; Zeng, J.; Yin, J.; Zuo, C.; Wen, P.; Chen, H.; Qiu, Y. Iron-Facilitated Surface Reconstruction to In Situ Generate Nickel–Iron Oxyhydroxide on Self-Supported FeNi Alloy

Fiber Paper for Efficient Oxygen Evolution Reaction. *Appl. Catal. B Environ.* **2021**, *286*, 119902.

(22) Li, D.; Tu, W.; Jin, Y.; Wang, C.; Jin, S.; Liu, W.; Song, E.; Peng, Z.; Xu, J.; Feng, L. Mechanism from Catalyst to Catalysis on Ir-Segregated $SrIrO_3$ for Hydrogen Evolution Reaction. *Appl. Catal. B Environ.* **2025**, *365*, 124961.

(23) Mattinen, M.; Schröder, J.; D'Acunto, G.; Ritala, M.; Jaramillo, T. F.; Stevens, M. B.; Bent, S. F. Dynamics of Precatalyst Conversion and Iron Incorporation in Nickel-Based Alkaline Oxygen Evolution Reaction Catalysts. *Cell Rep. Phys. Sci.* **2024**, *5*, 102284.

(24) Louie, M. W.; Bell, A. T. An Investigation of Thin-Film Ni–Fe Oxide Catalysts for the Electrochemical Evolution of Oxygen. *J. Am. Chem. Soc.* **2013**, *135*, 12329–12337.
